# Rapid Photoracemization of Chiral Alkyl Aryl Sulfoxides

**DOI:** 10.1021/acs.joc.1c02320

**Published:** 2021-11-20

**Authors:** Kosho Makino, Kumi Tozawa, Yuki Tanaka, Akiko Inagaki, Hidetsugu Tabata, Tetsuta Oshitari, Hideaki Natsugari, Hideyo Takahashi

**Affiliations:** † Faculty of Pharmaceutical Sciences, Tokyo University of Science, 2641 Yamazaki, Noda-shi, Chiba 278-8510, Japan; ‡ Department of Chemistry, Tokyo Metropolitan University, 1-1 Minami-Osawa, Hachioji, Tokyo 192-0397, Japan; § Faculty of Pharma Sciences, Teikyo University, 2-11-1 Kaga, Itabashi-ku, Tokyo 173-8605, Japan; ∥ Graduate School of Pharmaceutical Science, The University of Tokyo, 7-3-1 Hongo, Bunkyo-ku, Tokyo 113-0033, Japan

## Abstract

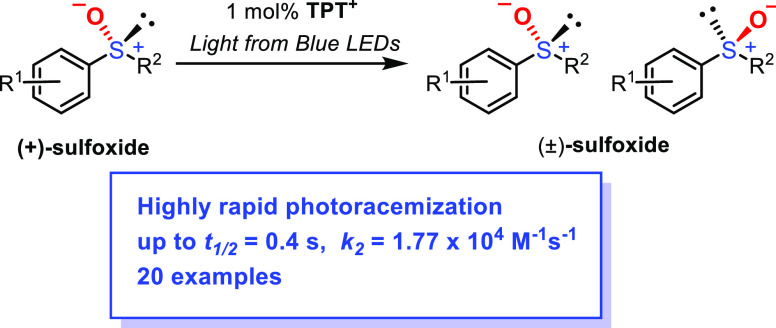

The photoracemization of chiral alkyl aryl sulfoxides with a photosensitizer has not been sufficiently investigated thus far. Therefore, in this study, a rapid photoracemization reaction of enantiopure alkyl aryl sulfoxides using 1 mol % 2,4,6-triphenylpyrylium tetrafluoroborate (TPT^+^) was developed. Various substitution patterns were tolerated and every racemization reaction proceeded extremely fast (*k*_2_ = 1.77 × 10^4^–6.08 × 10^1^ M^–1^ s^–1^, *t*_1/2_ = 0.4–114 s). Some chiral sulfoxides with easily oxidizable functional groups are not appropriate for this photoisomerization. The electrochemical potentials of the functional groups, determined via cyclic voltammetry, are useful for predicting the reactive or nonreactive groups in this photoracemization reaction. A theoretical study was conducted to clarify the sp^2^-like nature of S of the sulfoxide cation radical, which makes photoracemization easier.

## Introduction

Chiral sulfoxides are important bioactive compounds and intermediates in chemical reactions.^1^ The thermal stability of chiral sulfoxides is one of their characteristic properties. It has been elucidated that the pyramidal inversion of a sulfur center requires 159.1–171.7 kJ/mol, which entails substantially extreme conditions (temperatures of around 200 °C).^2^ However, the racemization of chiral sulfoxides by photoirradiation is also possible.^3^ The envisaged reaction mechanism is the inversion of the pyramidal center of sulfur or α-cleavage and recombination of the radical fragments.^4^ Since the initial reports by Mislow et al.,^5^ the pyramidal inversion of some alkyl aryl sulfoxides in the presence of a photosensitizer has been investigated.^6^ In that case, it was concluded that racemization occurs in an exciplex between the photosensitizer and sulfoxide.^7^ Recently, Lanzalunga’s group has reported that the use of *N*-methyl quinolinium tetrafluoroborate (NMQ^+^) enabled partial racemization up to 33% ee via 5 min of irradiation^8^ (*k*_2_ = 3.60 × 10^–1^ M^–1^ s^–1^, *t*_1/2_ = 24.5 min);^9^ they showed electron transfer processes involving the reversible formation of sulfoxide radical cations. These results prompted us to investigate the photoracemization reaction in the presence of a photosensitizer. We anticipated that quick racemization of sulfoxides should be applicable to technology, such as the dynamic kinetic resolution.^10^ Herein, we report the high-speed photoracemization of chiral alkyl aryl sulfoxides using a photosensitizer 2,4,6-triphenylpyrylium tetrafluoroborate (TPT^+^). Some sulfoxides with specific functional groups resist photoracemization, and this is appropriately assessed based on cyclic voltammograms.

## Results and Discussion

### Preparation of Alkyl Aryl Sulfoxides

**Scheme 1:**
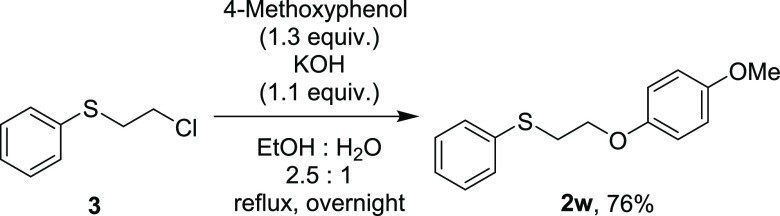
Preparation of **2w** from **3**

For the preparation of alkyl aryl sulfoxides **1**, the corresponding alkyl aryl sulfides **2** were oxidized. Although most of sulfides **2** were purchased, sulfide **2w** was prepared from commercially available alkyl chloride **3** using Williamson etherification (Scheme 1).

**Scheme 2:**
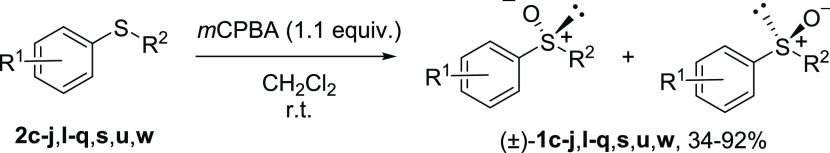
Preparation of **1** from **2**

As shown in Scheme 2, except for commercially available **1a**,^11−13^**1b**,^11−14^**1k**,^11,15^ and **1t**,^15^ oxidation of sulfides **2c**–**2j**, **2l**–**2q**, **2s**, **2u**, and **2w** proceeds smoothly to provide corresponding sulfoxides **1** as racemates (34–92%).

**Scheme 3:**
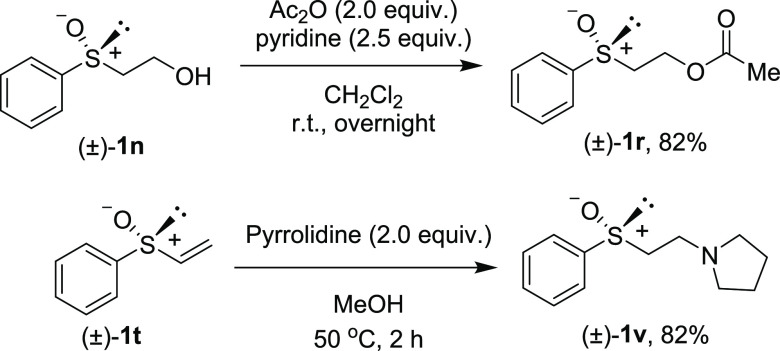
Preparation of **1r** and **1v**

Furthermore, sulfoxides with the functionalized alkyl moiety **1r**(16) and **1v** were synthesized from sulfoxides **1n** and **1t**, respectively (Scheme 3).

Each chiral sulfoxide (+)-**1a–w** was obtained by chiral high-performance liquid chromatography (HPLC) separation of the corresponding racemates **1a–w** (see the Supporting Information).

### Photoracemization of Alkyl Aryl Sulfoxides

First, we evaluated catalysts (**A**–**H**) for the photoisomerization of enantiopure methyl *p*-tolyl sulfoxide{(+)-**1a**} (>98% ee) in MeCN (Table 1). The enantiomeric ratio was determined by chiral HPLC. Upon irradiation of a 10 mM solution of (+)-**1a** using an 18 W blue light-emitting diode (LED) (λ = 425 nm) for 10 min, no reaction was observed without the photosensitizer (entry 1). The widely used photosensitizers perylene diimide (**A**), 9-cyano anthracene (**B**), thioxanthone (**C**), and fluorescein (**D**) were ineffective at the maximum absorption wavelength of the photosensitizer (entries 2–5). However, the addition of Mes-Acr-Me^+^ (**E**), DDQ (**F**), or 6-MeO-NMQ^+^ (**G**) at 1 mol % induced racemization at the optimal wavelength (entries 6–8). HPLC and ^1^H NMR analyses confirmed that no residual product, except for the racemate of **1a**, was obtained. Notably, TPT^+^ (**H**) gave the most desirable results (entries 9 and 10). Upon irradiation with 365 nm light, racemization occurred very quickly (*k*_2_ = 1.36 × 10^3^ M^–1^ s^–1^, *t*_1/2_ = 5 s) (entry 9).

**Table 1:**
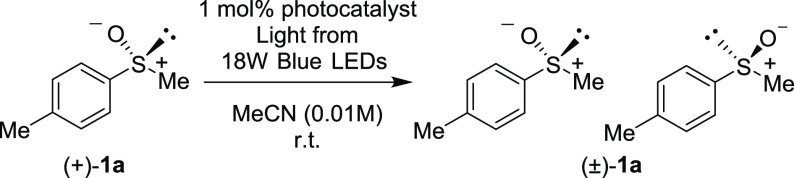
Screening of Reaction Conditions for Racemization of 1a^11−13^

entry	catalyst	wavelength (nm)	*t*_1/2_ (s)	*k*_2_ (M^–1^ s^–1^)
1		425		
2	A	525–530		
3	B	365		
4	C	380		
5	D	450–455		
6	E	425	106	6.57 × 10^1^
7	F	380	71	9.82 × 10^1^
8	G	365	30	2.28 × 10^2^
9	H	365	5	1.36 × 10^3^
10	H	425	2	3.19 × 10^3^

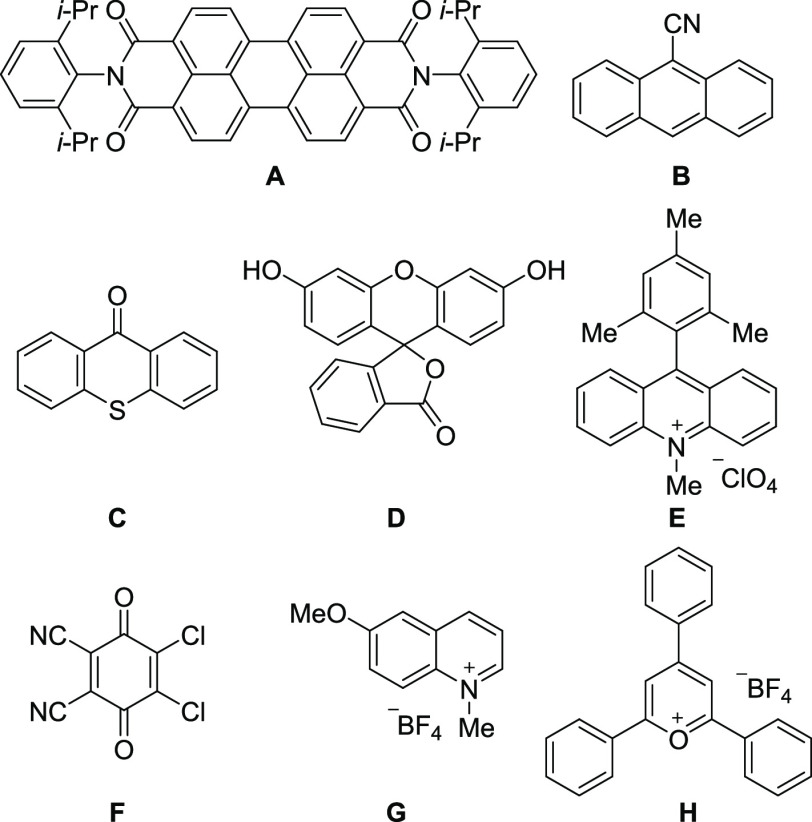

**Scheme 4:**
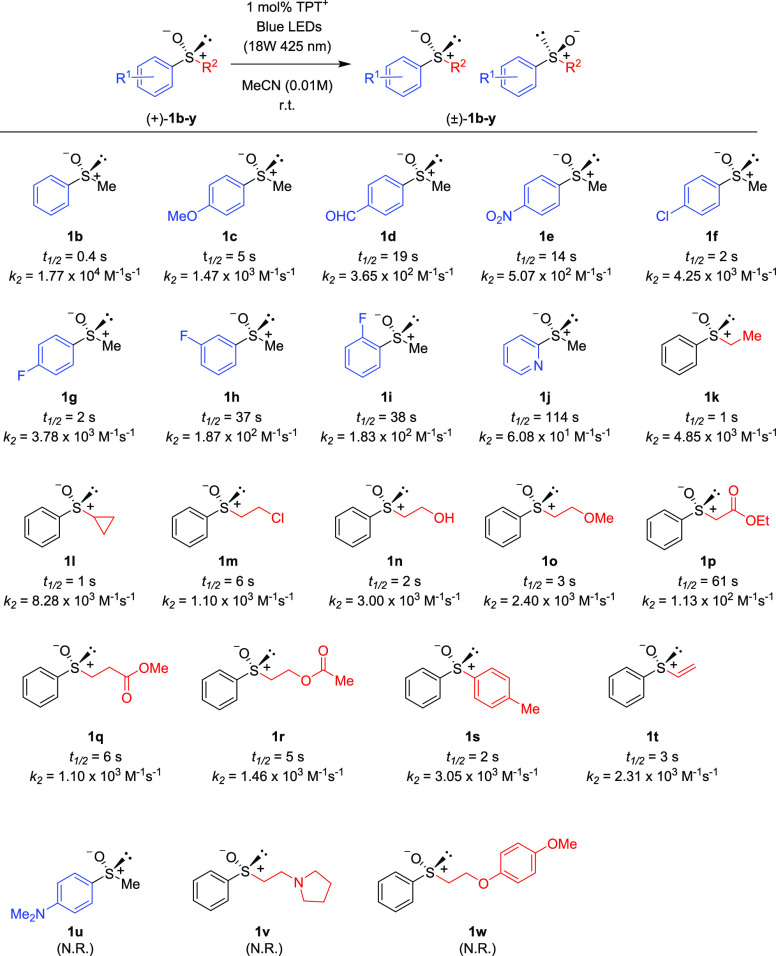
Substrate Scope of Racemization of Sulfoxides

Furthermore, irradiation with 425 nm light induced the most rapid interconversion of the sulfoxides (*k*_2_ = 3.19 × 10^3^ M^–1^ s^–1^, *t*_1/2_ = 2 s) (entry 10). The reaction rates of racemization were examined, and the reactions were shown to follow first-order kinetics. Thus, the second-order rate constants, *k*_2_, were calculated based on the pseudo-first-order rate constants, *k*_obs_ (see the Supporting Information). Having determined the optimized conditions, we investigated the substrate scope (Scheme 4).

In addition to methyl phenyl sulfoxide (**1b**),^11−14^ aromatic moieties containing various functional groups were tolerated (**1c**,^11−13^**1d**,^17^**1e**,^11,12,14^**1f**,^11,12^**1g**,^12^**1h**,^12^**1i**,^12^**1j**,^12^). Additionally, alkyl groups attached to the sulfur atom (**1k**,^11,15^**1l**^14^) and functionalized alkyl (**1m**,^11^**1n**,^12^**1o**,^18^**1p**,^17^**1q**,^19^**1r**,^16^), tolyl (**1s**^20^), and vinyl (**1t**^15^) groups were also rapidly racemized. Investigation of the reaction scope revealed that various substitution patterns were tolerated and every racemization reaction proceeded extremely fast (*k*_2_ = 1.77 × 10^4^–6.08 × 10^1^ M^–1^ s^–1^, *t*_1/2_ = 0.4–114 s). Although most functional groups were tolerated, no photoracemization was observed in several sulfoxides (**1u**–**w**). HPLC and ^1^H NMR analyses confirmed quantitatively that the starting chiral materials were recovered after 10 min of irradiation. Certain functional groups (dimethylamino, pyrrolidyl, anisyloxy) appeared to hinder photoracemization. The observed drastic effect of the functional groups on the reactivity prompted us to examine the electrochemical potentials of sulfoxides via cyclic voltammetry.

### Cyclic Voltammograms of Alkyl Aryl Sulfoxides

All alkyl aryl sulfoxides exhibited irreversible cyclic voltammograms (see the Supporting Information). It was found that the cyclic voltammograms of **1a**–**t** were similar, whereas those of **1u**–**w** were different.

**Figure 1:**
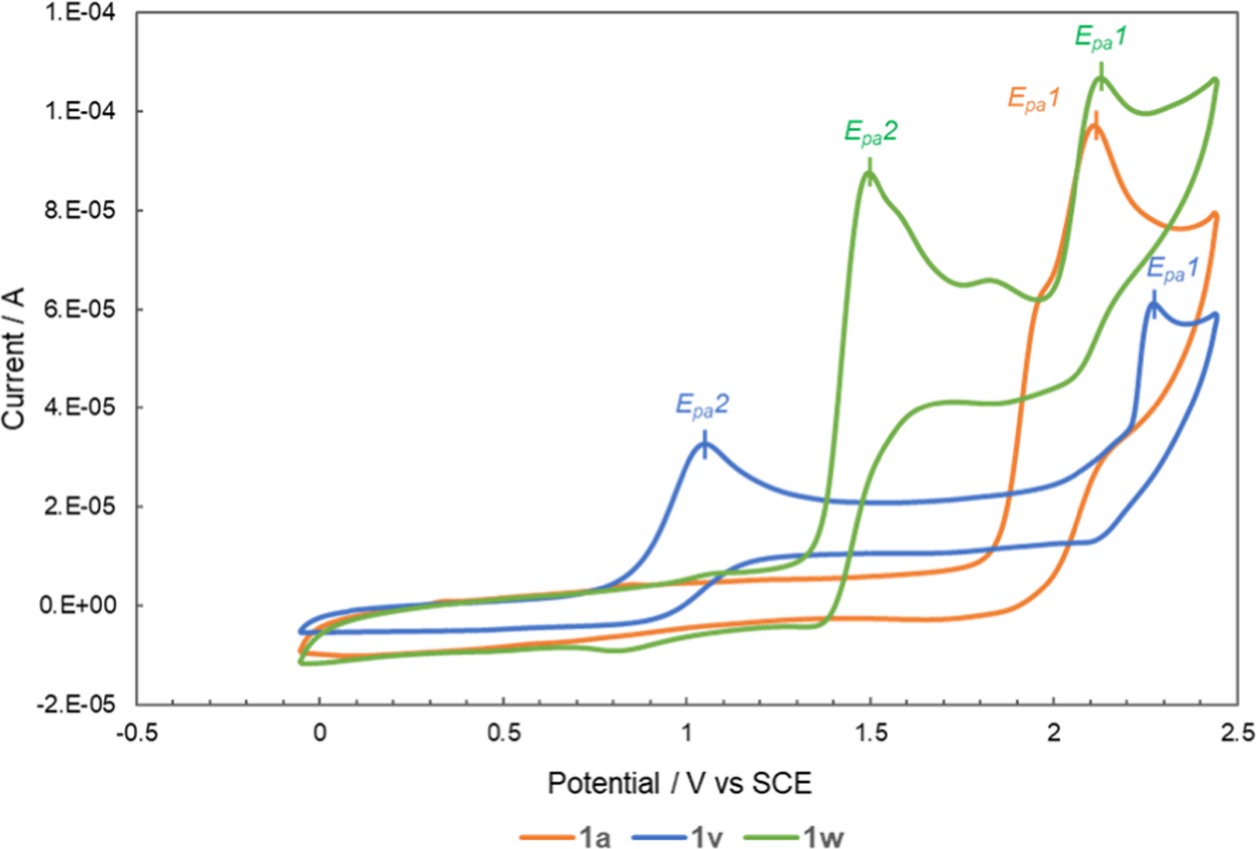
Cyclic voltammograms of **1a**, **1v**, and **1w** in MeCN.

**Scheme 5:**
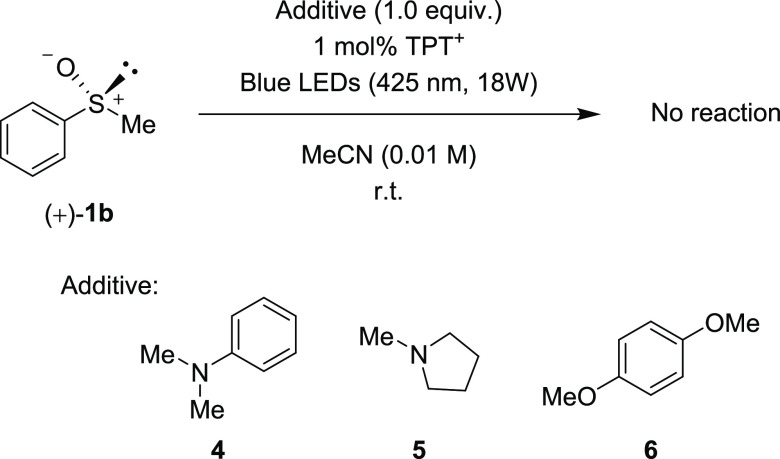
Photoreaction of (+)-**1b** in the Presence of Compounds **4**–**6**

The cyclic voltammograms of **1a** are shown in Figure 1. In **1a**–**t**, the highest peak (*E*_pa_1) was observed around +1.94 to +2.25 V against the saturated calomel electrode (SCE). This common peak (*E*_pa_1) was attributed to the sulfoxide. By contrast, **1v** and **1w** exhibited additional peaks at lower potentials. In the cyclic voltammograms of **1v**, the peak observed at a lower potential (*E*_pa_2 = +1.05 V vs SCE) was comparable to that of the pyrrolidinyl group. Similarly, in the cyclic voltammograms of **1w**, the peak observed at a lower potential (*E*_pa_2 = +1.51 V vs SCE) was comparable to that of the 4-methoxyphenoxyl group. For a rough analysis of the electrochemical potential of each functional group above, the data reported by Nicewicz’s group^21^ were used as a reference. It should be noted that the additional oxidation occurring at other functional groups is observed at a lower potential (*E*_pa_2) compared with that of the sulfoxide group (*E*_pa_1). Hence, they were more susceptible to oxidation. In accordance with this interpretation, when the photoreaction was performed in the alkyl aryl sulfoxides **1u**–**w**, the more easily oxidized groups should be oxidized first. Therefore, the sulfoxide that is less susceptible to oxidation might evade photoracemization. To confirm this hypothesis, the photoracemization of (+)-**1b** was examined in the presence of the additives **4** (*N*,*N*-dimethylaniline), **5** (1-methyl pyrrolidine), and **6** (1,4-dimethoxybenzene), which are contained in **1u**–**w** as functional groups (Scheme 5). When 1 equivalent of compound **4** was added to the MeCN solution of chiral methyl phenyl sulfoxide (+)-**1b**, irradiation (425 nm, 18 W, 1 mol % TPT^+^) for 10 min caused no reaction, and (+)-**1b** was recovered.^22^ The addition of compounds **5** and **6** produced the same results.

In the cyclic voltammograms of compounds **4**–**6**, a lower peak (*E*_pa_) than that of sulfoxide was observed (compound **4**: +0.88 V vs SCE, compound **5**: +1.01 V vs SCE, compound **6**: +1.46 V vs SCE) (see the Supporting Information). It was clarified that the presence of easily oxidizable compounds inhibited photoracemization of chiral sulfoxide intermolecularly. Therefore, compounds **1u**–**w**, in which compounds **4**–**6** were contained as functional groups, have correspondingly lower potential peaks (*E*_pa_2) in cyclic voltammograms. Thus, it was elucidated that chiral alkyl aryl sulfoxides with easily oxidizable functional groups are not appropriate for such photoisomerization. It was also suggested that the electrochemical potentials of the functional groups, determined via cyclic voltammetry, are useful for predicting the reactive or nonreactive groups in this photoracemization reaction.

### Calculation Study

**Figure 2:**
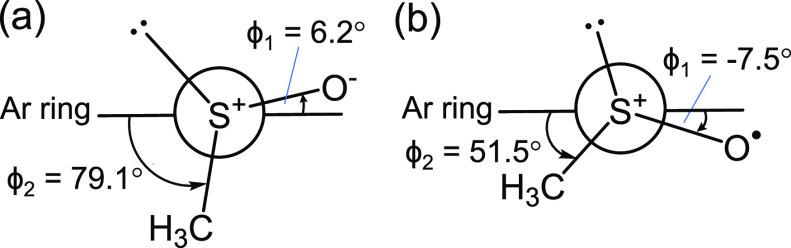
Selected dihedral angles of (a) (+)-**1a** and (b) (+)-**1a**^•+^.

**Figure 3:**
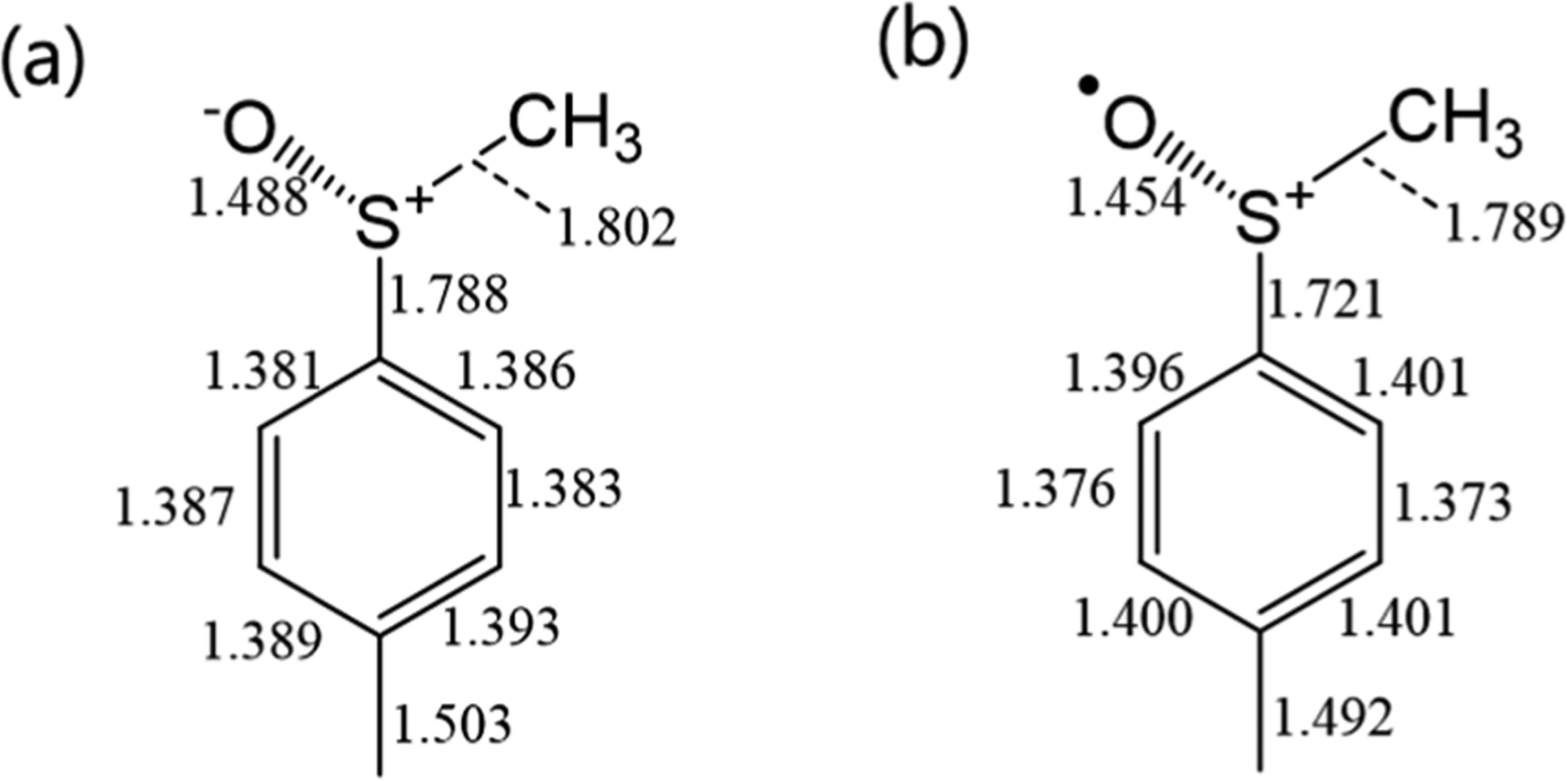
Selected bond lengths of (a) (+)-**1a** and (b) (+)-**1a**^•+^.

**Figure 4:**
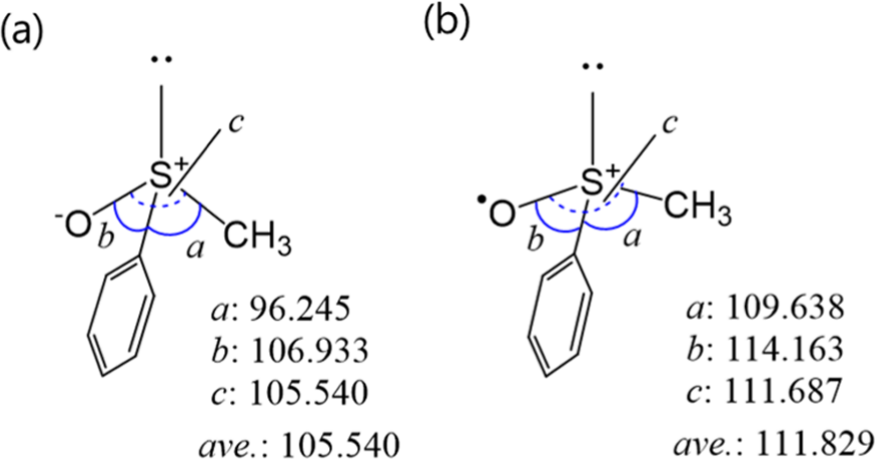
Selected angles of (a) (+)-**1a** and (b) (+)-**1a**^•+^.

**Figure 5:**
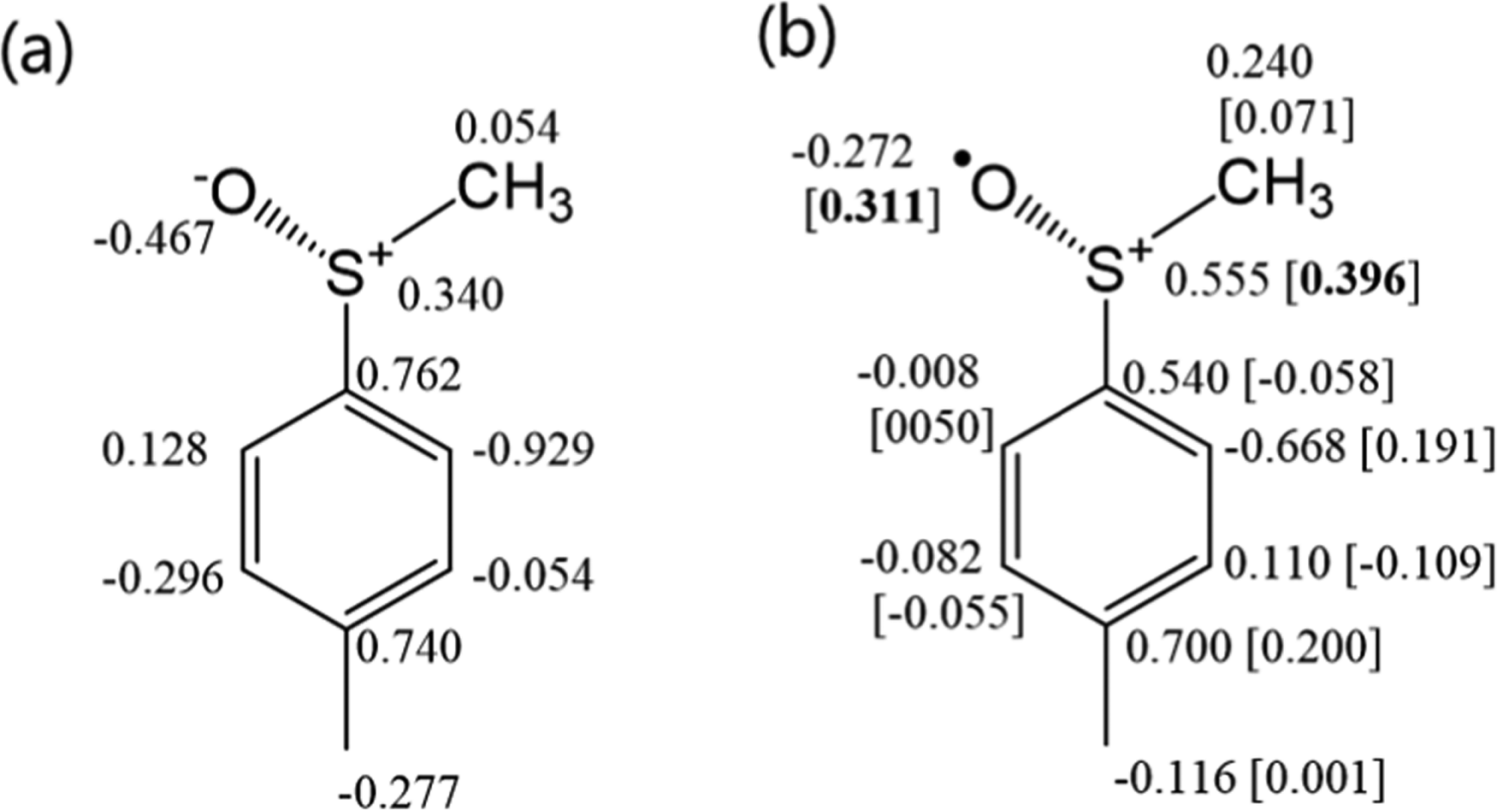
Mulliken charges and spin densities (in square brackets) with hydrogens summed into heavy atoms for (a) (+)-**1a** and (b) (+)-**1a**^•+^.

We hypothesized a reaction mechanism based on the proposal of Lanzalunga’s group;^8^ the oxidation of chiral sulfoxide (+)-**1a** by excited TPT (*TPT^+^) would form a sulfoxide radical cation, which is a key intermediate in the racemization process. Thus, the geometry of the sulfoxide radical cation of **1a** was optimized by density functional theory (DFT) calculations. Computations were performed with Gaussian 16,A.03,^23^ and the geometries of (+)-1a* and its 1e-oxidized cation radicals ((+)-**1a**^•+^) were optimized using the M05-2X functional^24^ with the 6-311+G(3df,2p) basis set.^25^ The structures and selected bond lengths and angles are depicted in Figures 2–5 and the Supporting Information (Figures S2–S3 and Tables S2–S3). The method and the basis set were chosen according to prior reports of calculations on various sulfoxides that gave reliable structural and thermodynamic parameters.^26^ Additionally, among the methods and basis sets tested,^27^ the S–O bond lengths of the optimized structure provided reasonable results with the structural parameters of aryl sulfoxides reported in the CCDC database.^28^ In the structures of (+)-**1a** and [(+)-**1a**]^•+^, the sulfur atom constituted a pyramidal geometry, with S–O being nearly coplanar to the Ar ring; ϕ_1_: 6.2 and −7.5°, respectively. The defined torsion angle ϕ_2_ for (+)-**1a** was 79.1°, whereas that of the corresponding radical cation was 51.5°, which was significantly lower than that of the neutral one (Figure 2). The structural change indicates that the geometry of S changed from an sp^3^-like to sp^2^-like nature (Figures 3 and 4), resulting in a more planar placement of CAr, S, O, and CCH_3_ than that of the neutral species. These structural changes should lower the inversion barrier of the R_2_S–O group to make the racemization process much easier for the radical cation than for the neutral sulfoxide. Additionally, the increase in positive charges at the S–R fragment in [(+)-**1a**]^•+^ from the parent neutral substrate and the localization of spin densities on the S and O atoms support the CV data, indicating that 1e oxidation occurs at the sulfoxide unit (Figure 5).

## Conclusions

In conclusion, we achieved the rapid photoracemization of chiral alkyl aryl sulfoxides in the presence of 1 mol % TPT^+^. The acceleration effect of TPT^+^ was extremely high, and a wide substrate scope was elucidated. However, some sulfoxides with functional groups (dimethylamino, pyrrolidyl, anisolyloxy) resisted racemization. It was revealed that the electrochemical potentials of these functional groups determined by cyclic voltammograms are lower than those of sulfoxides. The presence of such easily oxidizable functional groups hindered photoracemization of sulfoxides since they should be oxidized in advance of sulfoxides. It is suggested that the electrochemical potentials of the functional groups, determined via cyclic voltammetry, are useful for predicting the reactive or nonreactive nature of this photoracemization reaction. Furthermore, DFT calculations of the geometry of the sulfoxide radical cation were performed to clarify the sp^2^-like nature of S of the sulfoxide, which supported the reaction mechanism proposed by Lanzalunga’s group. The rapid photoracemization of chiral sulfoxides should be applied to a novel dynamic kinetic resolution to provide the desired optical isomers efficiently, which is now under investigation.

## Experimental Section

### General Experimental Procedure

All reagents were purchased from commercial suppliers and used as received. Compounds **1a**, **1b**, **1k**, **1t**, **4**, **5**, and **6** are commercially available. Reaction mixtures were stirred magnetically, and the reactions were monitored by thin-layer chromatography (TLC) on precoated silica gel plates. For the reactions that require heating, an oil bath was used. Column chromatography was performed using silica gel (45–60 μm). Extracted solutions were dried over anhydrous MgSO_4_ or Na_2_SO_4_. Solvents were evaporated under reduced pressure. NMR spectra were recorded on a spectrometer at 400 MHz for ^1^H NMR and 100 MHz for ^13^C NMR at 296 K unless otherwise stated. Chemical shifts are given in parts per million (ppm) downfield from tetramethylsilane as an internal standard, and coupling constants (*J*) are reported in hertz (Hz). Splitting patterns are abbreviated as follows: singlet (s), doublet (d), triplet (t), quartet (q), multiplet (m), and broad (br). The high-resolution mass spectra (HRMS) were recorded using an ESI/TOF mass spectrometer. IR spectra were recorded on an FTIR spectrometer equipped with ATR (diamond). Melting points were recorded on a melting point apparatus and were uncorrected. For irradiation with LEDs, an optical irradiation device (EvoluChemTM PhotoRedOx Box) and chemistry screening kits (HepatoChem Inc., Massachusetts) were used. Chiral sulfoxides were irradiated at rt with blue LED light (28 mW/cm^2^) at a distance of 5 cm from the light source.

### General Procedure for Preparation of Chiral Sulfoxides **1a–w**

For chiral HPLC charts of **1a–w** and their optical properties, see the Supporting Information. The optical purity of each chiral sulfoxide was determined by chiral HPLC analysis. For chiral HPLC charts of the chiral sulfoxides, see the Supporting Information.

### General Procedure for Racemization of **1a–w**

A piece of vial tube containing a solution of TPT^+^ (0.08 mg, 0.0002 mmol, 1.0 mol %) and (*R*)**-**(+)-**1a** (3.08 mg, 0.02 mmol) in MeCN was irradiated in a photoreactor equipped with blue LEDs (420 nm; 18 W) using PhotoRedOx Box EvoluChem at 25 °C. The extent of racemization was determined by HPLC analysis on a CHIRALPAK IG column using 100% acetonitrile as the mobile phase (flow rate = 0.5 mL/min) (retention times of 15.9 and 18.5 min for (*R*)-(+)-**1a** and (*S*)-(−)**-1a**, respectively).

Since compounds **1a–u** and sulfides except for **2w** are known compounds and purchased from commercial suppliers, their ^1^ H NMR and ^13^C NMR characterization data are omitted.

### General Procedure for the Preparation of **1a–w**

To a stirred solution of methyl *p*-tolyl sulfide (1.0 mL, 7.5 mmol) in CH_2_Cl_2_ (25 mL) was added *m*CPBA (8.25 mmol, 1.1 equiv). After the mixture was stirred at rt for 1 h, the residue was diluted with aq. NaHCO_3_ and extracted with CH_2_Cl_2_. The extract was washed with brine, dried, and concentrated in vacuo. The residue was purified by column chromatography to afford ***rac*****-1a** as a colorless oil (888 mg, 5.75 mmol, 77% yield).

### Preparation and Characterization of Sulfoxides **1a–u**

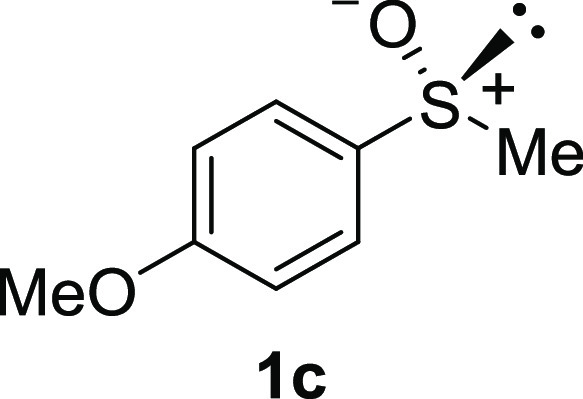



#### 4-Methoxyphenyl Methyl Sulfoxide (***rac*****-1c**)

The general procedure was followed starting from 4-methoxyphenyl methyl sulfide (0.5 mL, 3.60 mmol) to give the crude product, which was purified by column chromatography on silica gel (hexane/ethyl acetate 1:3) to yield ***rac*****-1c** as a colorless oil (415 mg, 2.44 mmol, 69% yield).
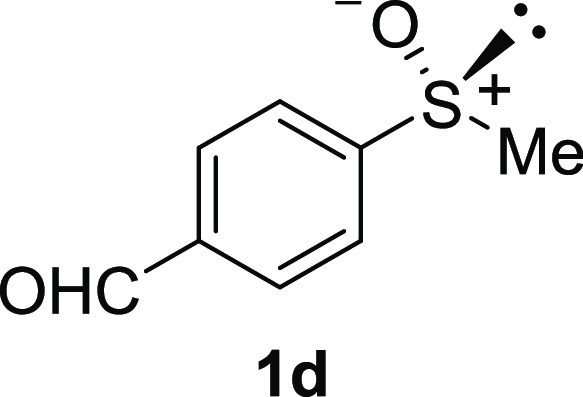


#### 4-Formylphenyl Methyl Sulfoxide (***rac*****-1d**)

The general procedure was followed starting from 4-(methylthio)benzaldehyde (1.30 mL, 10.0 mmol) to give the crude product, which was purified by column chromatography on silica gel (hexane/ethyl acetate 2:1) to yield the desired *rac*-**1d** as a white solid (1.15 g, 6.82 mmol, 68% yield, mp 88–90 °C).
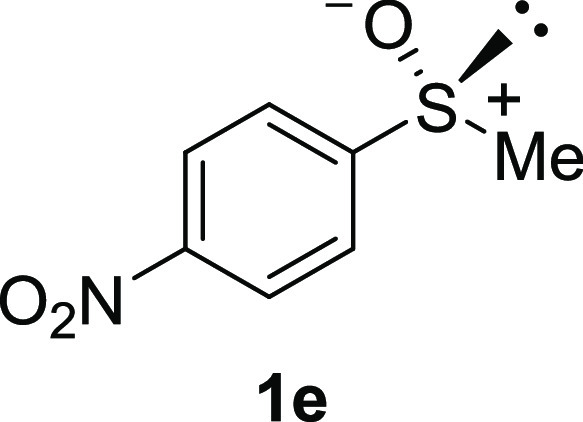


#### 4-Nitrophenyl Methyl Sulfoxide (***rac*****-1e**)

The general procedure was followed starting from 2-chloroethyl phenyl sulfide (1.69 g, 10 mmol) to give the crude product, which was purified by column chromatography on silica gel (hexane/ethyl acetate 2:1) to yield the desired *rac*-**1e** as a white solid (0.64 g, 3.43 mmol, 34% yield, mp 153–155 °C).
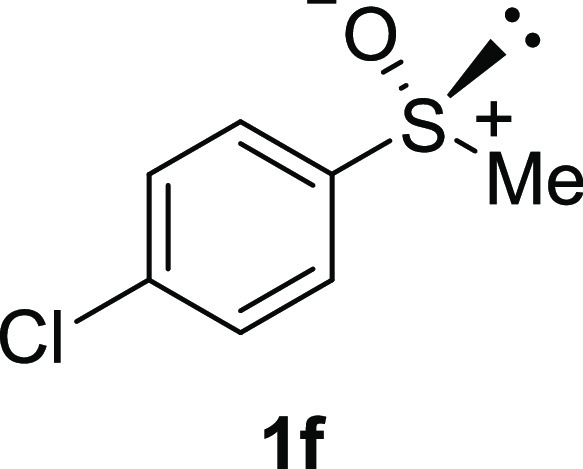


#### 4-Chlorophenyl Methyl Sulfoxide (***rac*****-1f**)

The general procedure was followed starting from 2-chloroethyl phenyl sulfide (0.50 g, 3.85 mmol) to give the crude product, which was purified by column chromatography on silica gel (hexane/ethyl acetate 1:3) to yield the desired *rac*-**1f** as a colorless oil (0.527 g, 3.02 mmol, 78% yield).
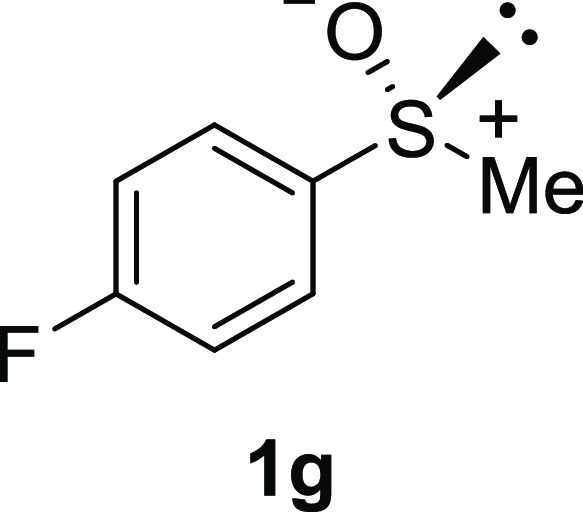


#### 4-Fluorophenyl Methyl Sulfoxide (***rac*****-1g**)

The general procedure was followed starting from 4-fluorophenyl methyl sulfide (0.50 mL, 4.08 mmol) to give the crude product, which was purified by column chromatography on silica gel (hexane/ethyl acetate 1:3) to yield the desired *rac*-**1g** as a colorless oil (376 mg, 2.38 mmol, 58% yield).
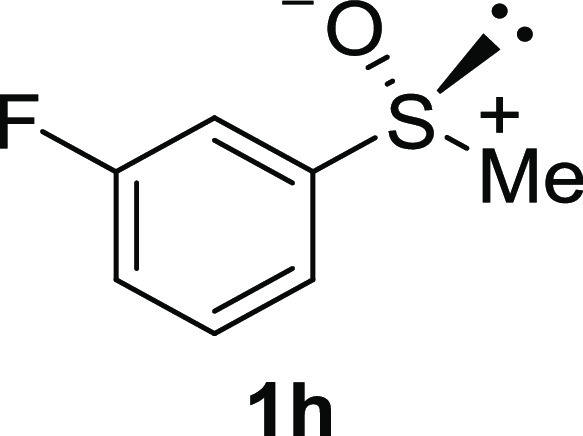


#### 3-Fluorophenyl Methyl Sulfoxide (***rac*****-1h**)

The general procedure was followed starting from 3-fluorophenyl methyl sulfide^29^ (1.22 mL, 10 mmol) to give the crude product, which was purified by column chromatography on silica gel (hexane/ethyl acetate 2:1) to yield the desired *rac*-**1h** as a colorless oil (1.27 g, 8.03 mmol, 80% yield).
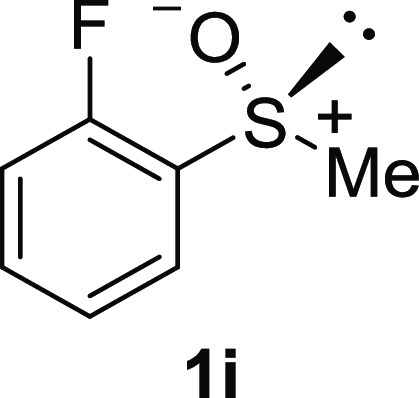


#### 3-Fluorophenyl Methyl Sulfoxide (***rac*****-1i**)

The general procedure was followed starting from 3-fluorophenyl methyl sulfide (1.22 g, 10 mmol) to give the crude product, which was purified by column chromatography on silica gel (hexane/ethyl acetate 2:1) to yield the desired *rac*-**1i** as a colorless oil (0.95 g, 5.98 mmol, 60% yield).
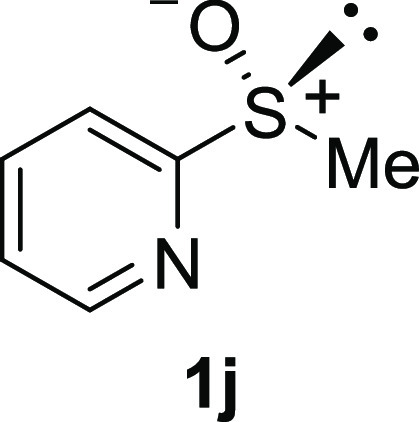


#### 2-Pyridyl Methyl Sulfoxide (***rac*****-1j**)

The general procedure was followed starting from methyl 2-pyridyl sulfide^29^ (2.23 mL, 20.0 mmol) to give the crude product, which was purified by column chromatography on silica gel (hexane/ethyl acetate 5:1) to yield the desired *rac*-**1j** as a colorless oil (1.86 g, 13.2 mmol, 66% yield).
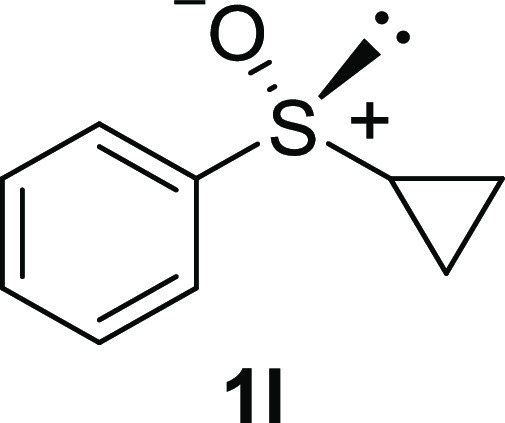


#### Cyclopropyl Phenyl Sulfoxide (***rac*****-1l**)

The general procedure was followed starting from cyclopropyl phenyl sulfide (1.0 mL, 7.00 mmol) to give the crude product, which was purified by column chromatography on silica gel (hexane/ethyl acetate 1:3) to yield the desired *rac*-**1l** as a colorless oil (1.03 g, 6.21 mmol, 80% yield).
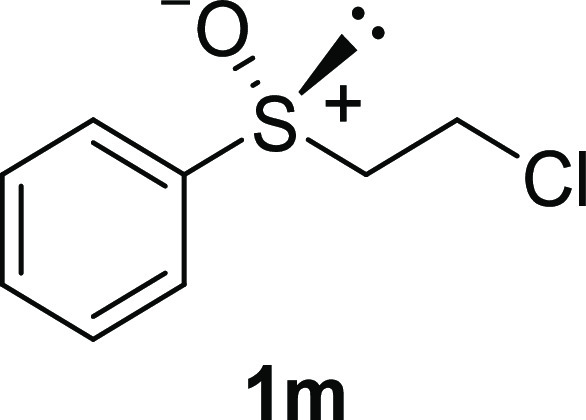


#### 2-Chloroethyl Phenyl Sulfoxide (***rac*****-1m**)

The general procedure was followed starting from 2-chloroethyl phenyl sulfide (1.46 mL, 10 mmol) to give the crude product, which was purified by column chromatography on silica gel (hexane/ethyl acetate 2:1) to yield the desired *rac*-**1m** as a colorless oil (1.65 g, 8.76 mmol, 88% yield).
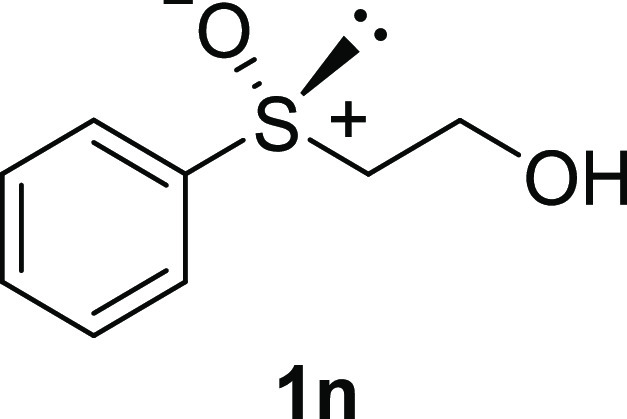


#### 2-Hydroxyethyl Phenyl Sulfoxide (***rac*****-1n**)

The general procedure was followed starting from **2-**hydroxyethyl phenyl sulfide (2.68 mL, 20 mmol) to give the crude product, which was purified by column chromatography on silica gel (dichloromethane/methanol 19:1) to yield the desired *rac*-**1n** as a colorless oil (1.20 g, 7.05 mmol, 35% yield).
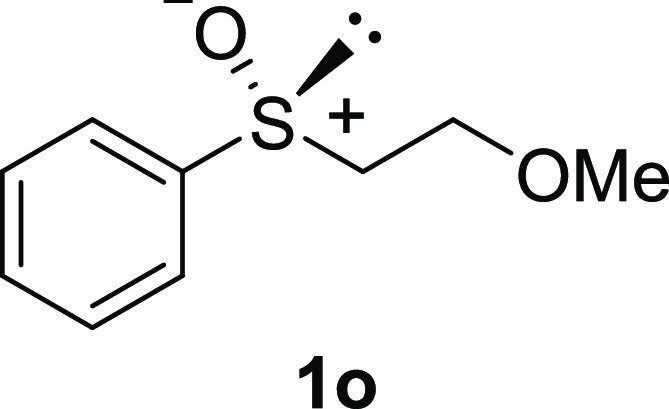


#### 2-Methoxyethyl Phenyl Sulfoxide (***rac*****-1o**)

The general procedure was followed starting from 2-methoxyethyl phenyl sulfide (968 mg, 5.75 mmol) to give the crude product, which was purified by column chromatography on silica gel (dichloromethane/methanol 9:1) to yield the desired *rac*-**1o** as a colorless oil (893 mg, 4.85 mmol, 84% yield).
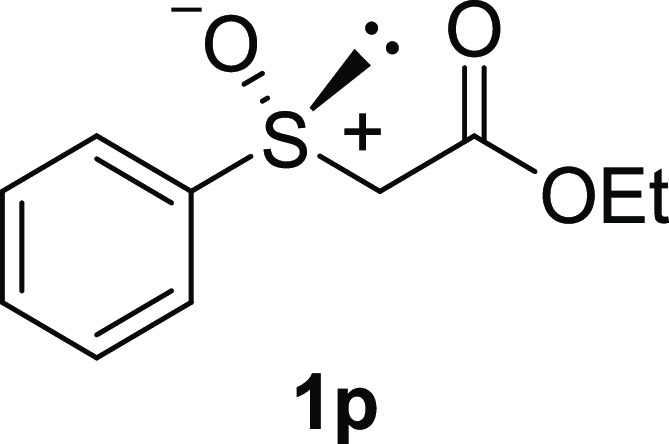


#### Ethyl Phenylsulfinyl Acetate (***rac*****-1p**)

The general procedure was followed starting from ethyl (phenylthio)acetate^30^ (0.97 mL, 5.6 mmol) to give the crude product, which was purified by column chromatography on silica gel (hexane/ethyl acetate 1:2) to yield the desired *rac*-**1p** as a colorless oil (1.00 g, 4.71 mmol, 84% yield).
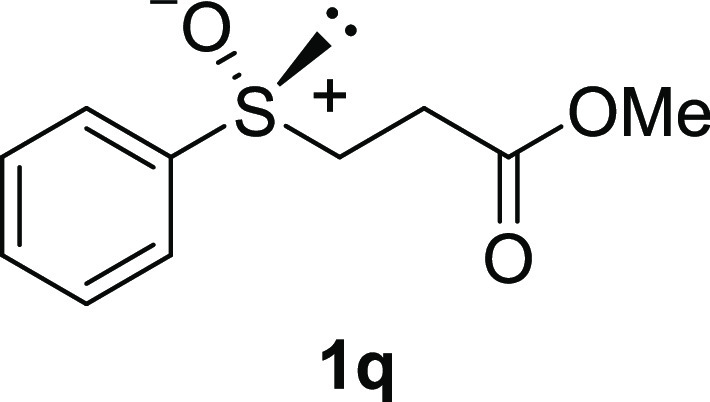


#### Methyl 3-(Phenylsulfinyl)propanoate (***rac*****-1q**)

The general procedure was followed starting from methyl 3-(phenylsulfinyl)propanoate (69.7 mg, 0.355 mmol) to give the crude product, which was purified by column chromatography on silica gel (hexane/ethyl acetate 1:1) to yield the desired *rac*-**1q** as a colorless oil (45.8 mg, 0.216 mmol, 61% yield).
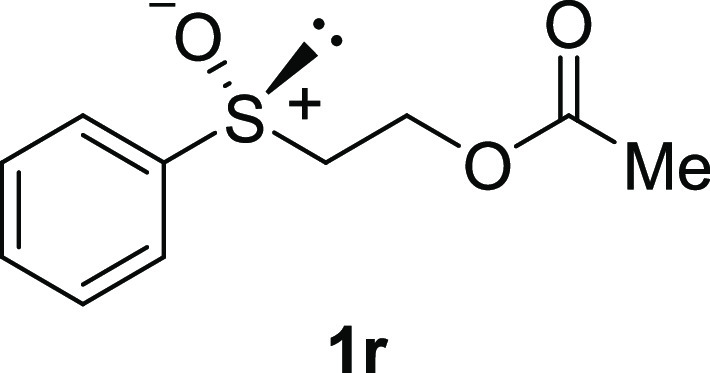


#### 1-Acetate 2-(Phenylsulfinyl)-ethanol (***rac*****-1r**)

To a stirred solution of 2-hydroxyethyl phenyl sulfoxide **1n** (340 mg, 2.0 mmol) in CH_2_Cl_2_ (5 mL) were added acetic anhydride (0.380 mL, 4.0 mmol, 2.0 equiv) and pyridine (0.40 mL, 5.0 mmol, 2.5 equiv). After the mixture was stirred at rt overnight, the mixture was treated with H_2_O and extracted with CH_2_Cl_2_. The extract was washed with brine, dried, and concentrated. The residue was purified by column chromatography (silica gel, hexane/ethyl acetate = 1:1) to afford **1r** (346 mg, 1.63 mmol, 82% yield) as a colorless oil.
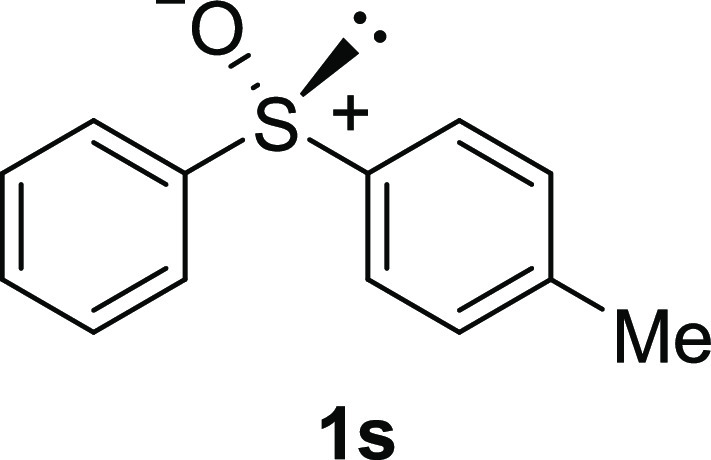


#### 4-Methyl Phenyl Sulfoxide (***rac*****-1s**)

The general procedure was followed starting from phenyl *p*-tolyl sulfide^31^ (4.0 mL, 21.7 mmol) to give the crude product, which was purified by column chromatography on silica gel (hexane/ethyl acetate 4:1) to yield the desired *rac*-**1s** as a white solid (3.07 g, 14.2 mmol, 65% yield, mp 64–66 °C).
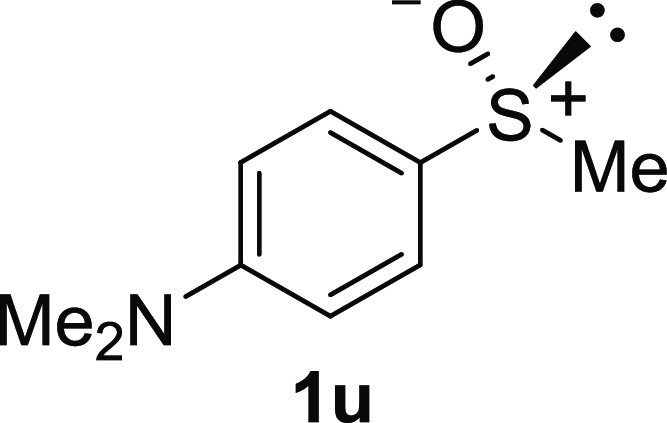


#### *N*,*N*-Dimethyl-4-(methylsulfinyl)benzenamine (***rac*****-1u**)^13^

The general procedure was followed starting from *N*,*N*-dimethyl-4-(methylthio)benzenamine^32^ (488 mg, 2.92 mmol) to give the crude product, which was purified by column chromatography on silica gel (dichloromethane/methanol 19:1) to yield the desired *rac*-**1u** as a white solid (395 mg, 2.16 mmol, 82% yield, mp 65–67 °C).
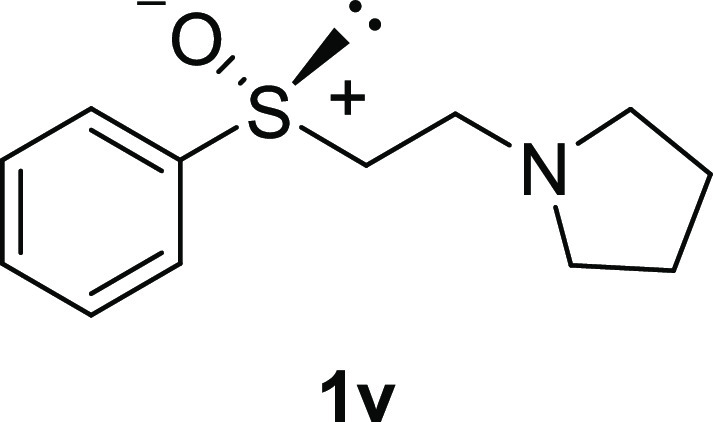


#### 1-[2-(Phenylsulfinyl)ethyl]-pyrrolidine (***rac*****-1v**)

To a stirred solution of pyrrolidine (1.29 mL, 15.6 mmol, 2.0 equiv) in MeOH (7.8 mL) was added phenyl vinyl sulfoxide **1t** (1.02 mL, 7.8 mol). After the mixture was stirred at 50 °C for 2 h, the solvent was evaporated under reduced pressure. The residue was purified by column chromatography (silica gel, CH_2_Cl_2_/MeOH = 20:1) to afford the desired *rac*-**1v** (1.44 g, 6.43 mmol, 82% yield) as a colorless oil: ^1^H NMR (CDCl_3_, 400 MHz) δ 7.61–7.66 (m, 2H), 7.48–7.53 (m, 3H), 2.88–3.05 (m, 3H), 2.59–2.67 (m, 1H), 2.51–2.54 (m, 4H), 1.73–1.82 (m, 4H). ^13^C{^1^H} NMR (CDCl_3_, 100 MHz) δ 144.2, 131.0, 129.3, 129.3, 124.2, 124.2, 56.9, 54.1, 54.1, 49.0, 23.6, 23.6; IR (ART) 2962, 2788, 1444, 997 cm^–1^; HRMS (ESI-TOF) *m*/*z*: [M + Na]^+^. Calcd for C_12_H_17_NOSNa 246.0923; found 246.0925.
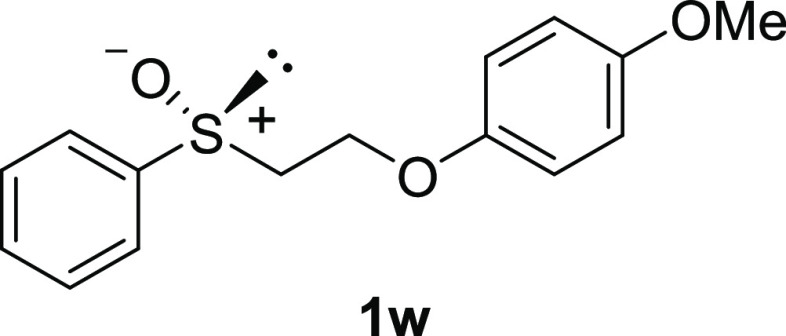


#### 1-Methoxy-4-[2-(phenylsulfinyl)ethoxy]benzene (***rac*****-1w**)

The general procedure was followed starting from 1-methoxy-4-[2-(phenylthio)ethoxy]-benzene (871 mg, 3.00 mmol) to give the crude product, which was purified by column chromatography on silica gel (hexane/ethyl acetate 1:1) to yield the desired *rac*-**1w** as a white solid (850 mg, 2.77 mmol, 92% yield, mp 92–95 °C): ^1^H NMR (CDCl_3_, 400 MHz) δ 7.65–7.69 (m, 2H), 7.52–7.54 (m, 3H), 6.82 (s, 4H), 4.43 (ddd, 1H, *J* = 5.2, 6.0, 12.8 Hz), 4.17 (ddd, 1H, *J* = 5.2, 5.2, 10.8 Hz), 3.77 (s, 3H), 3.12–3.24 (m, 2H). ^13^C{^1^H} NMR (CDCl_3_, 100 MHz) δ 154.4, 152.2, 143.9, 131.2, 131.2, 129.4, 129.4, 124.0, 124.0, 115.9, 115.9, 114.8, 114.8, 61.6, 57.5, 55.8; IR (ART) 1507, 1218, 1034, cm^–1^; HRMS (ESI-TOF) *m*/*z*: [M + Na]^+^. Calcd for C_15_H_16_O_3_SNa 299.0712; found 299.0711.
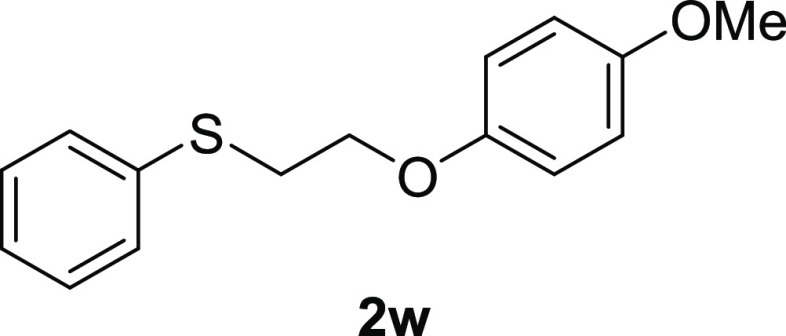


#### 1-Methoxy-4-[2-(phenylthio)ethoxy]-benzene (**2w**)

To a stirred solution of 4-methoxy phenol (1.66 g, 13.4 mmol, 1.34 equiv) in EtOH (25 mL) and H_2_O (10 mL) were added potassium hydroxide (726 mg, 11.0 mmol, 1.1 equiv) and 2-chloroethyl phenyl sulfide (1.46 mL, 10.0 mmol). After the mixture was stirred at reflux overnight, the mixture was filtered and washed with H_2_O. The residue was recrystallized with hot hexane to afford 1-methoxy-4-[2-(phenylthio)ethoxy]-benzene **2w** as a white solid (2.20 g, 7.59 mmol, 76% yield, mp 82–84 °C): ^1^H NMR (CDCl_3_, 400 MHz) δ 7.41 (d, 2H, *J* = 7.2 Hz), 7.30 (t, 2H, *J* = 7.2 Hz), 7.21 (t, 1H, *J* = 7.2 Hz), 6.81 (s, 4H), 4.10 (t, 2H, *J* = 7.6 Hz), 3.76 (s, 3H), 3.27 (t, 2H, *J* = 7.2 Hz). ^13^C{^1^H} NMR (CDCl_3_, 100 MHz) δ 154.2, 152.6, 135.6, 129.9, 129.9, 129.1, 129.1, 126.6, 115.8, 115.8, 114.7, 114.7, 67.5, 55.8, 33.0; IR (ART) 1190, 1021, 817, cm^–1^; HRMS (ESI) *m*/*z*: [M]^+^. Calcd for C_15_H_16_O_2_S 260.0871; found 260.0870.

## Supporting Information Available

Pseudo-first-order rate constants, *k*_obs_, and second-order rate constants, *k*_2_; electrochemical measurements, cyclic voltammograms of **1a**–**w**, **4**–**6**; computational details; chiral HPLC charts of **1a**–**w** and their optical properties; optical purity of (+)-**1a**–**w**; ^1^H-, ^13^C-, and 2D-NMR spectra of **1v**, **2**, **1w**; and recovery of **1u**–**w** after irradiation for 10 min (PDF)
