# Supplementary material for: Rapid Photoracemization of Chiral Alkyl Aryl Sulfoxides
Source: J Org Chem. 2021 Nov 20;86(23):17249–56. doi: 10.1021/acs.joc.1c02320 (PMC8650104; doi:10.1021/acs.joc.1c02320)
Supplement: Supplementary file 1 — jo1c02320_si_001.pdf [file jo1c02320_si_001.pdf]

Rapid Photoracemization of Chiral Alkyl Aryl Sulfoxides

Kosho Makino,[1] Kumi Tozawa,[1] Yuki Tanaka,[1] Akiko Inagaki,[2] Hidetsugu Tabata,[3] Tetsuta Oshitari,[2] Hideaki Natsugari,[3] Hideyo Takahashi*[1]

[1]Faculty of Pharmaceutical Sciences, Tokyo University of Science, 2641 Yamazaki, Noda-shi, Chiba 278-8510, Japan

[2]Department of Chemistry, Tokyo Metropolitan University, 1-1 Minami-Osawa, Hachioji, Tokyo 192-0397, Japan

[3]Faculty of Pharma Sciences, Teikyo University, 2-11-1 Kaga, Itabashi-ku, Tokyo 173-8605, Japan

[4]Graduate School of Pharmaceutical Science, The University of Tokyo, 7-3-1 Hongo, Bunkyo-ku, Tokyo 113-0033, Japan

hide-tak@rs.tus.ac.jp

Contents

1. **Pseudo-first-order rate constants, $k_{obs,}$ and second-order rate constants k_2………...S2**
2. **Electrochemical measurements…………………………………………………….S10**
3. **Cyclic voltammograms of 1a–w, 4–6………………………………………………S11**
4. **Computational details………………...........……………………...……………….…....S21**
5. **Chiral HPLC charts of 1a–w and their optical properties…………………………..S24**
6. **Optical purity of (+)-1a–w…………………………………………………………..S36**
7. **^{1}H-, ^{13}C-, and 2D-NMR spectra of 1v, 2, 1w…..………………..……………….……..S42**

1. Pseudo-first-order rate constants, k_{obs}, and second-order rate constants k_2

Table S1. Racemization rate (k_{obs}) of (+)-**1p** (0.01 M) with TPT^+ at r.t. in MeCN.

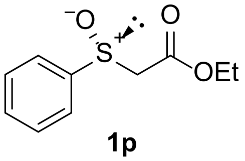

1p

TPT^+ (mol %)	k_{obs} (s^{-1})
0.1	1.03×10^{-3}
0.5	3.94×10^{-3}
0.75	8.90×10^{-3}
1	1.13×10^{-2}
1.25	1.84×10^{-2}

Figure S1. First-order dependence of the rate of racemization on TPT^+ for the reaction of (+)-**1p**

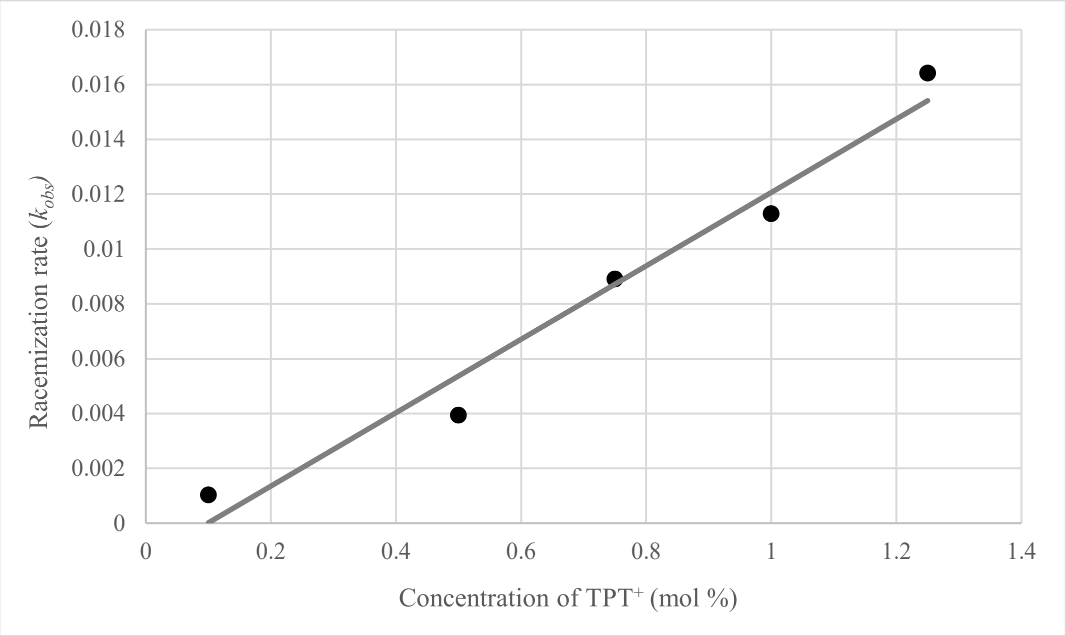

Racemization rates were shown to follow pseudo-first-order rate constants, k_{obs} were determined from the slope of the straight line obtained when -ln(% ee_t/% ee_0) is plotted against time.

$$-\ln (\% \, ee_t / \% \, ee_0) = k_{obs} \, t$$

$$k_{obs} = k_2 \, [TPT^+]_0$$

$$t_{1/2} = \ln (2)/k_{obs}$$

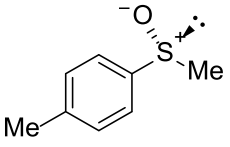

1a

$k_{obs} = 3.19 \times 10^{-1}\ s^{-1}$

$k_2 = 3.19 \times 10^{3}\ M^{-1}s^{-1}$

$t_{1/2} = 2\ s$

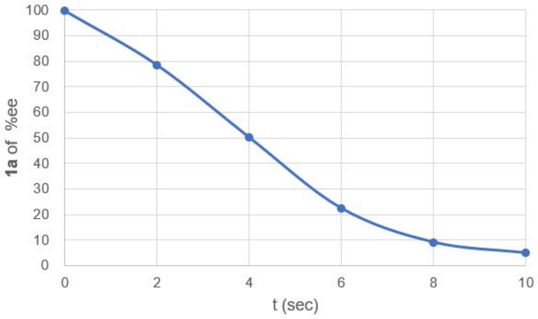

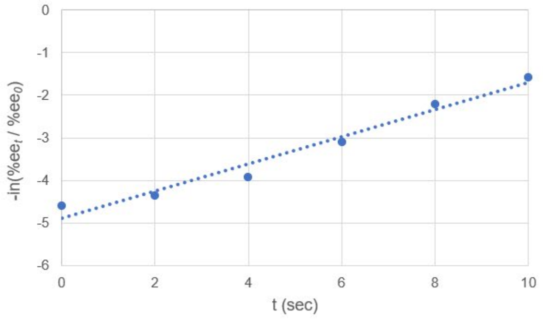

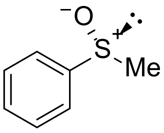

1b

$k_{obs} = 1.77\ s^{-1}$

$k_2 = 1.77 \times 10^{4}\ M^{-1}s^{-1}$

$t_{1/2} = 0.4\ s$

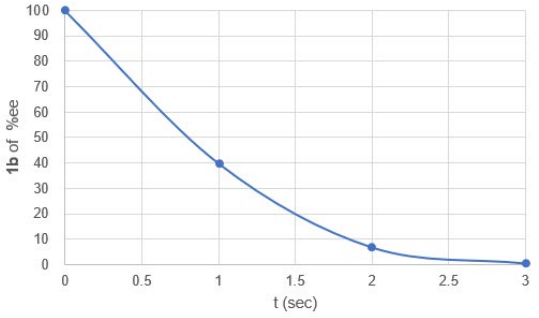

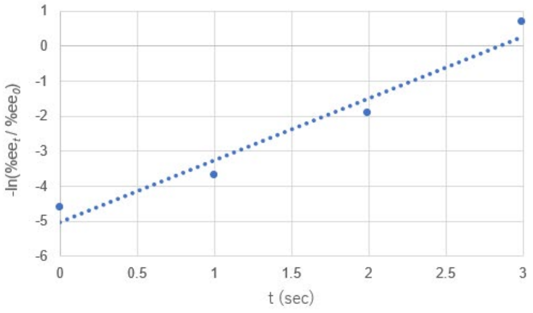

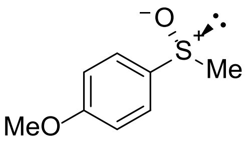

1c

$k_{obs} = 1.47 \times 10^{-1}\ s^{-1}$

$k_2 = 1.47 \times 10^{3}\ M^{-1}s^{-1}$

$t_{1/2} = 5\ s$

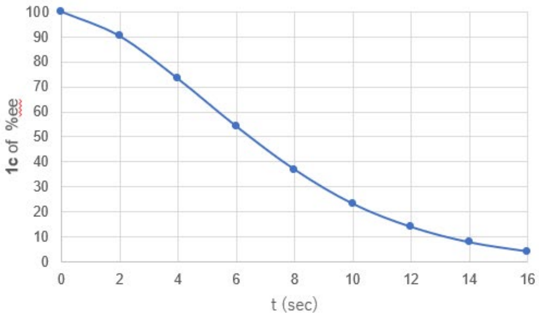

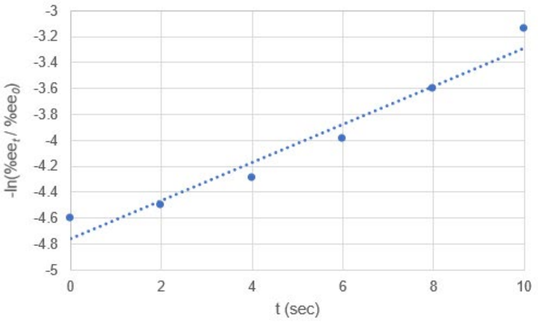

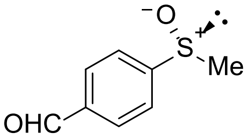

1d

$k_{obs} = 3.64 \times 10^{-2}\ s^{-1}$

$k_2 = 3.65 \times 10^{2}\ M^{-1}s^{-1}$

$t_{1/2} = 19$ s

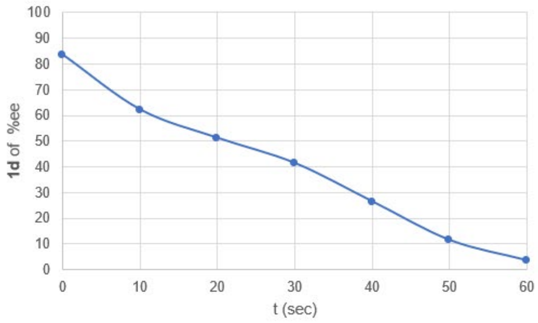

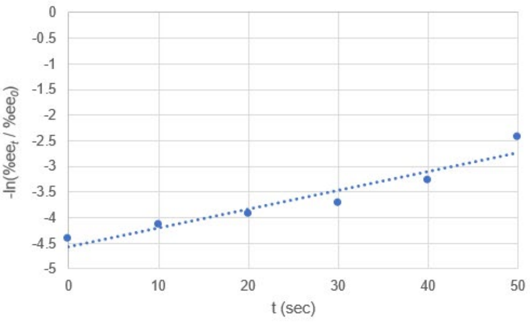

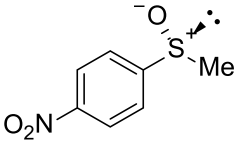

1e

$k_{obs} = 5.07 \times 10^{-2}\ s^{-1}$

$k_2 = 5.07 \times 10^{2}\ M^{-1}s^{-1}$

$t_{1/2} = 14$ s

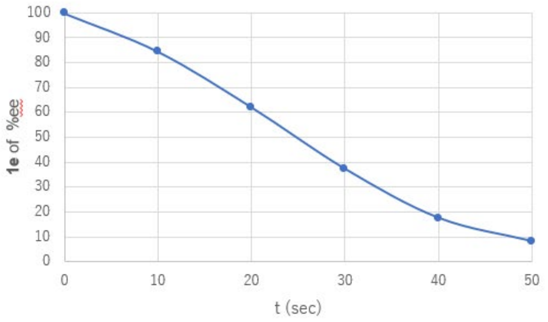

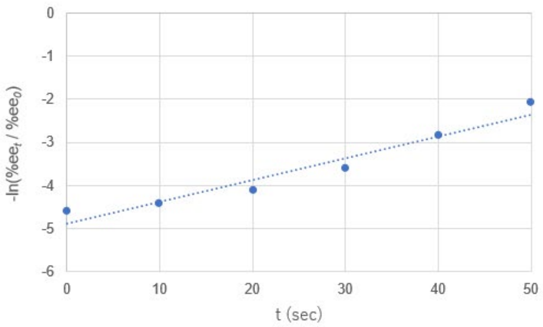

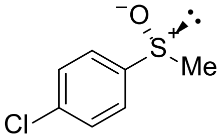

1f

$k_{obs} = 4.24 \times 10^{-1}\ s^{-1}$

$k_2 = 4.25 \times 10^{3}\ M^{-1}s^{-1}$

$t_{1/2} = 2$ s

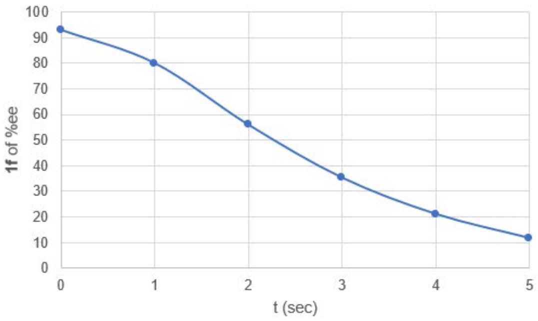

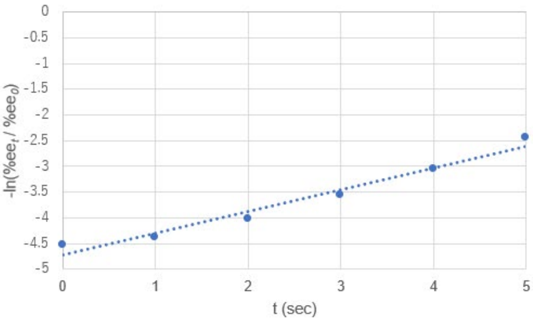

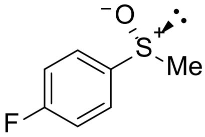

1g

$k_{obs} = 3.78 \times 10^{-1}\ s^{-1}$

$k_2 = 3.78 \times 10^{3}\ M^{-1}s^{-1}$

$t_{1/2} = 2\ s$

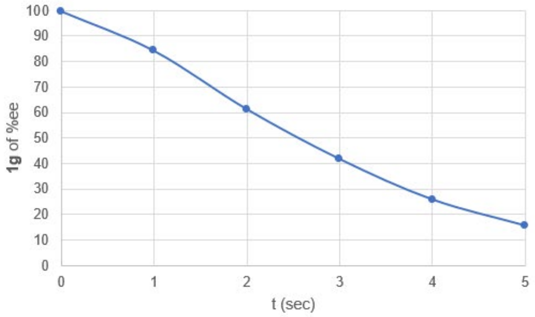

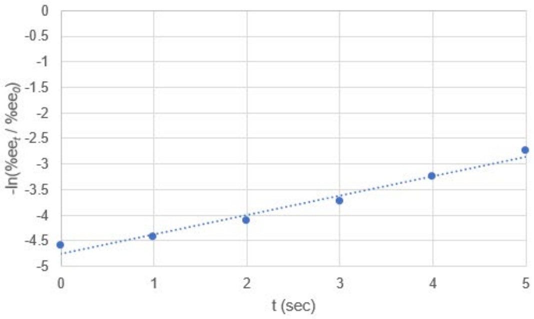

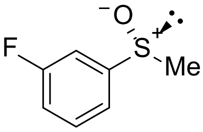

1h

$k_{obs} = 1.87 \times 10^{-2}\ s^{-1}$

$k_2 = 1.87 \times 10^{2}\ M^{-1}s^{-1}$

$t_{1/2} = 37\ s$

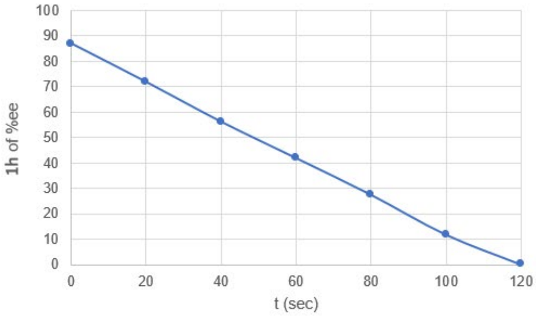

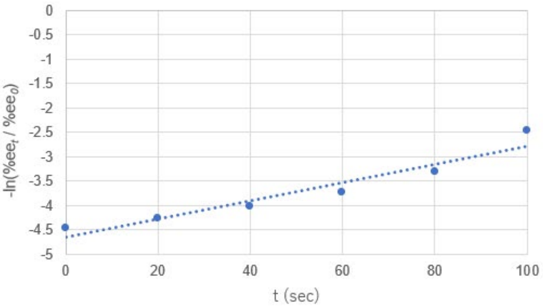

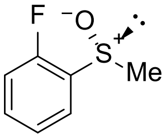

1i

$k_{obs} = 1.83 \times 10^{-2}\ s^{-1}$

$k_2 = 1.83 \times 10^{2}\ M^{-1}s^{-1}$

$t_{1/2} = 38\ s$

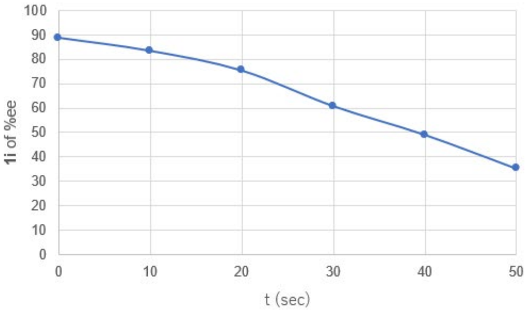

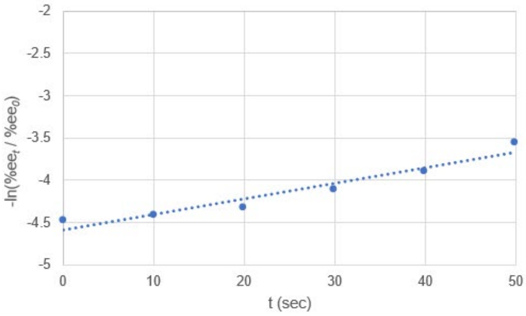

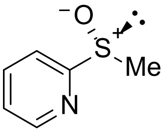

1j

$k_{obs} = 6.08 \times 10^{-3}\ s^{-1}$

$k_2 = 6.08 \times 10^{1}\ M^{-1}s^{-1}$

$t_{1/2} = 114\ s$

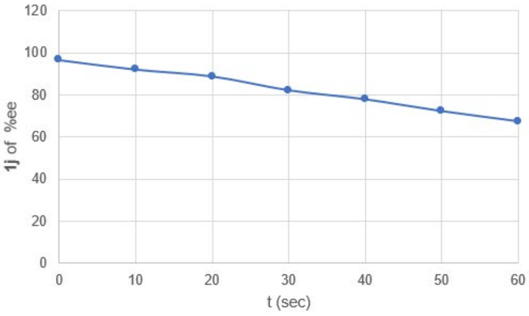

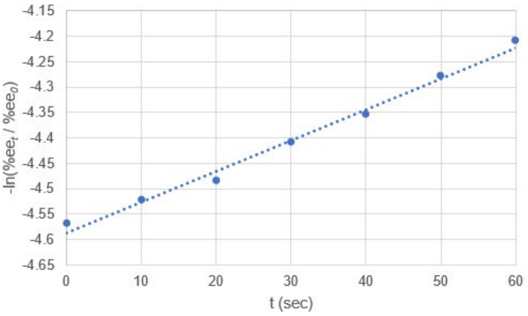

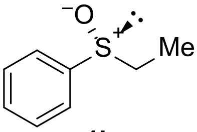

1k

$k_{obs} = 4.85 \times 10^{-1}\ s^{-1}$

$k_2 = 4.85 \times 10^{3}\ M^{-1}s^{-1}$

$t_{1/2} = 1\ s$

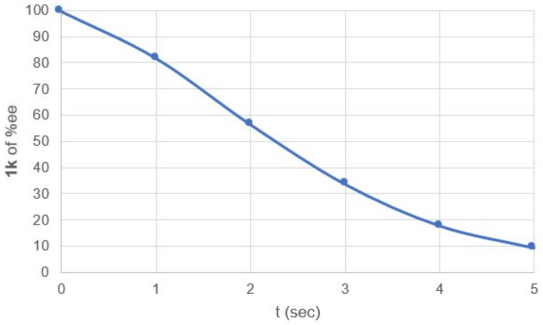

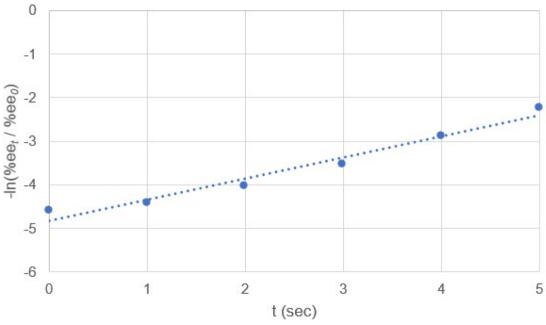

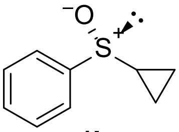

1l

$k_{obs} = 8.28 \times 10^{-1}\ s^{-1}$

$k_2 = 8.28 \times 10^{3}\ M^{-1}s^{-1}$

$t_{1/2} = 1\ s$

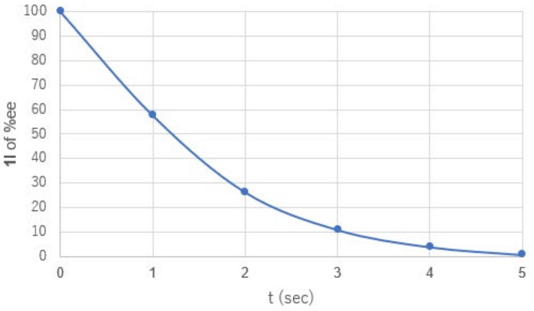

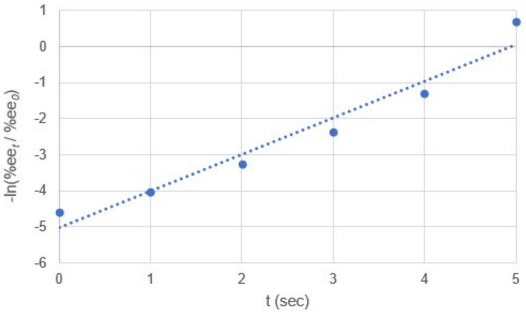

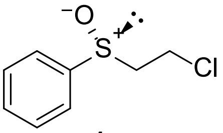

1m

$k_{obs} = 1.10 \times 10^{-1}\ s^{-1}$

$k_2 = 1.10 \times 10^{3}\ M^{-1}s^{-1}$

$t_{1/2} = 6\ s$

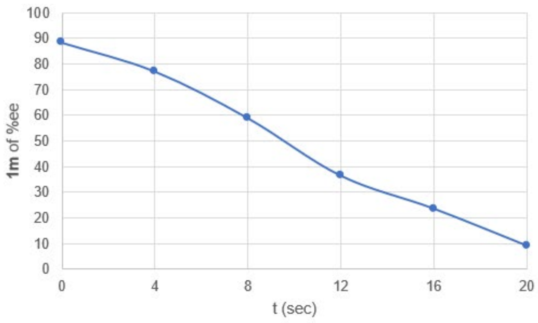

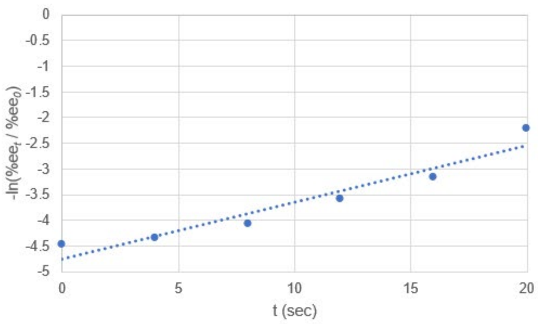

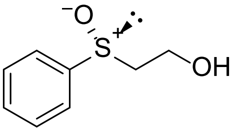

1n

$k_{obs} = 3.00 \times 10^{-1}\ s^{-1}$

$k_2 = 3.00 \times 10^{3}\ M^{-1}s^{-1}$

$t_{1/2} = 2\ s$

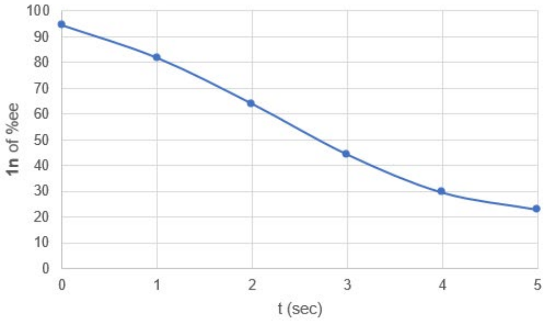

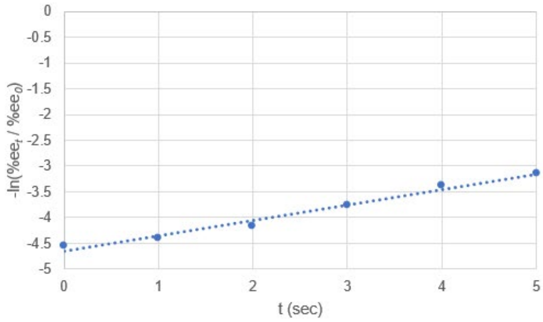

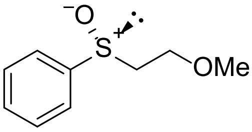

1o

$k_{obs} = 2.40 \times 10^{-1}\ s^{-1}$

$k_2 = 2.40 \times 10^{3}\ M^{-1}s^{-1}$

$t_{1/2} = 3\ s$

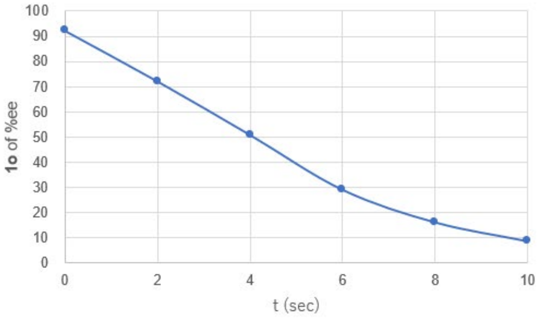

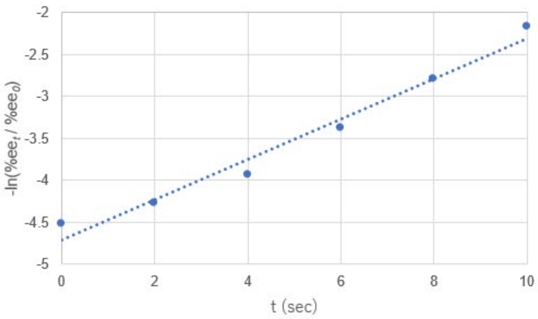

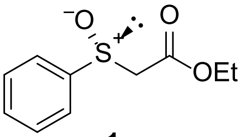

1p

$k_{obs} = 1.13 \times 10^{-2}\ s^{-1}$

$k_2 = 1.13 \times 10^{2}\ M^{-1}s^{-1}$

$t_{1/2} = 61\ s$

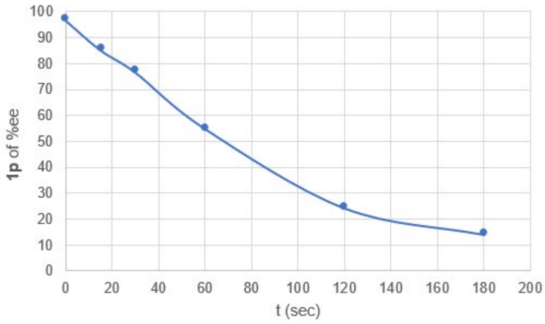

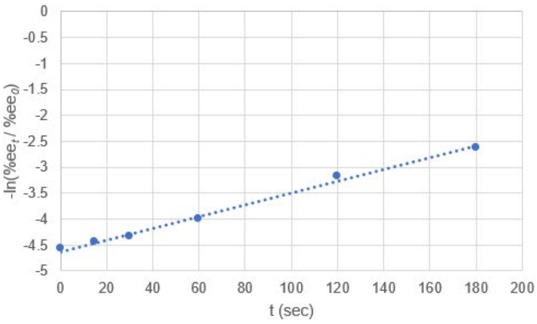

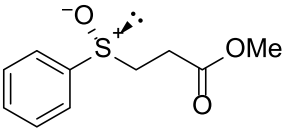

1q

$k_{obs} = 1.10 \times 10^{-1}\ s^{-1}$

$k_2 = 1.10 \times 10^{3}\ M^{-1}s^{-1}$

$t_{1/2} = 6\ s$

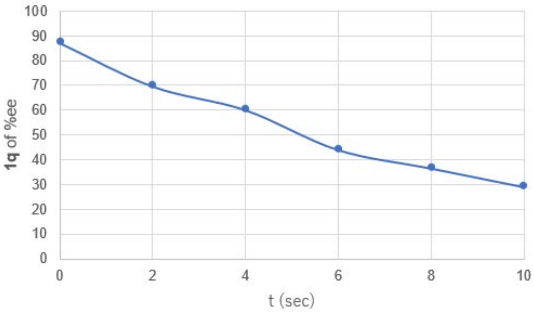

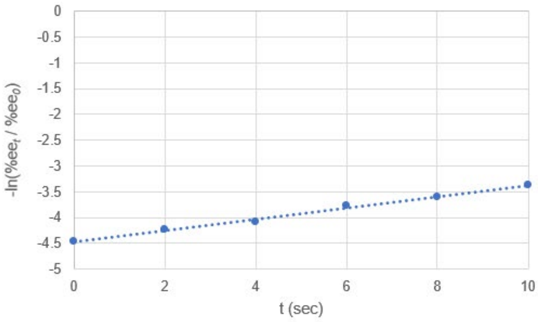

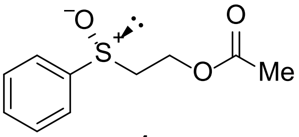

1r

$k_{obs} = 1.46 \times 10^{-1}\ s^{-1}$

$k_2 = 1.46 \times 10^{3}\ M^{-1}s^{-1}$

$t_{1/2} = 5\ s$

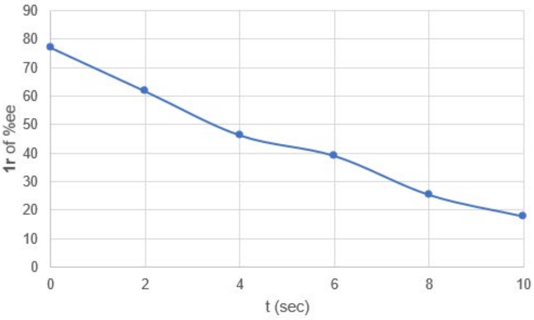

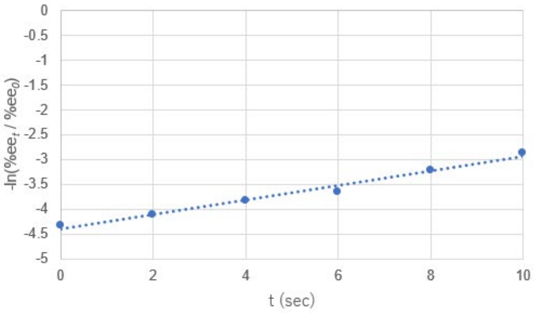

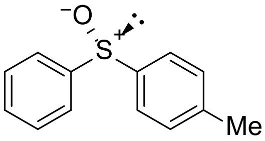

1s

$k_{obs} = 3.05 \times 10^{-1}\ s^{-1}$

$k_2 = 3.05 \times 10^{3}\ M^{-1}s^{-1}$

$t_{1/2} = 2\ s$

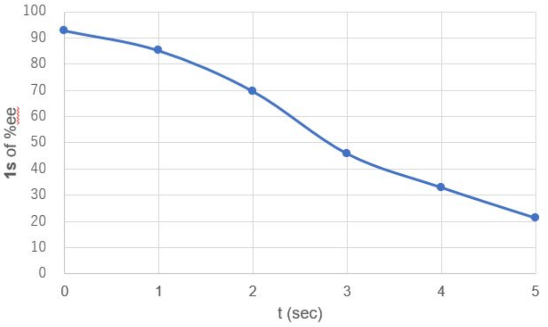

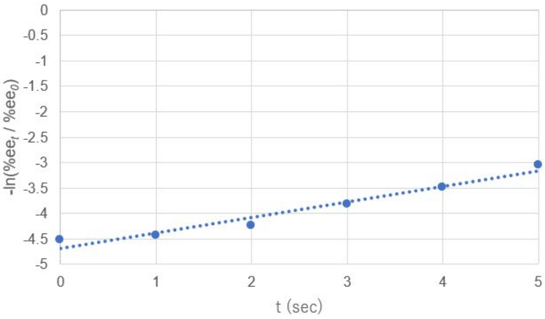

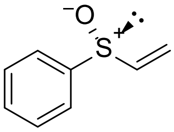

1t

$k_{obs} = 2.31 \times 10^{-1}\ s^{-1}$

$k_2 = 2.31 \times 10^{3}\ M^{-1}s^{-1}$

$t_{1/2} = 3\ s$

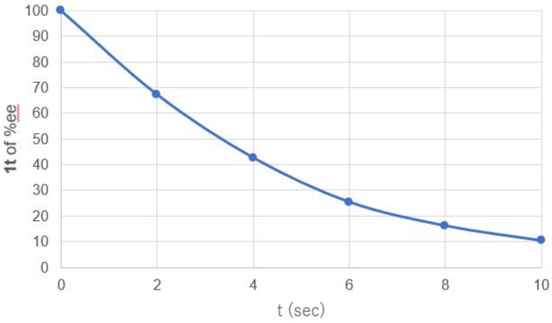

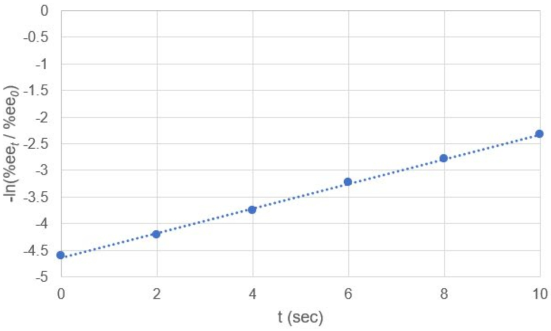

2. Electrochemical measurements

Electrochemical potentials were obtained with a standard set of conditions to main internal consistency. Cyclic voltammograms were collected with a computer-controlled potentiostat ECstat-301 (EC Frontier Co., Ltd). Samples were prepared with 0.021 mmol of substrate in 7 mL of 0.1 M tetra-*n*-butylammonium perchlorate in dry, degassed acetonitrile. Measurements employed a glassy carbon working electrode, platinum wire counterelectrode, 0.1 M $AgNO_3$, 0.1M tetra-*n*-butylammonium perchlorate-silver nitrate reference electrode, and a scan rate of 25 mV/s. Reductions were measured by scanning potentials in the negative direction and oxidations in the positive direction; the glassy carbon electrode was polished between each scan. The ferrocene/ferrocenium couple (Fc/Fc^+) was also measured in the same electrochemical system, and the electrode potential was reported as values referred to the standard potential of the system. The reference value was converted to SCE by adding 0.447 V (in CH_3CN).

3. Cyclic voltammograms of 1a–w, 4–6.

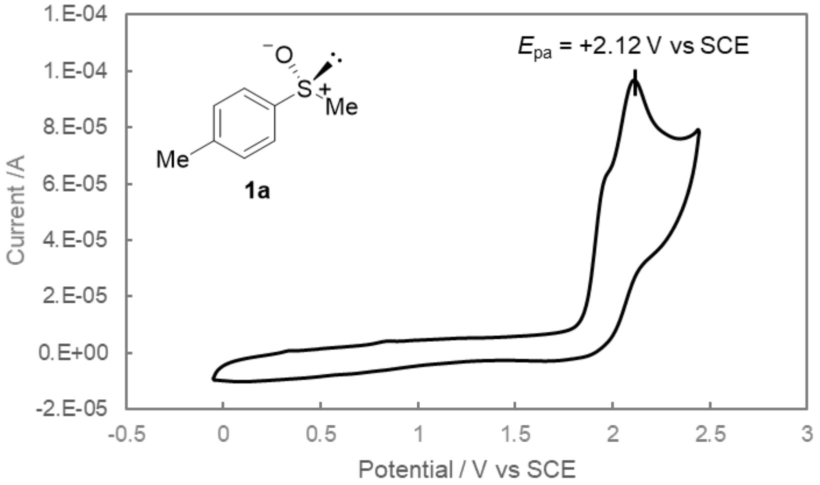

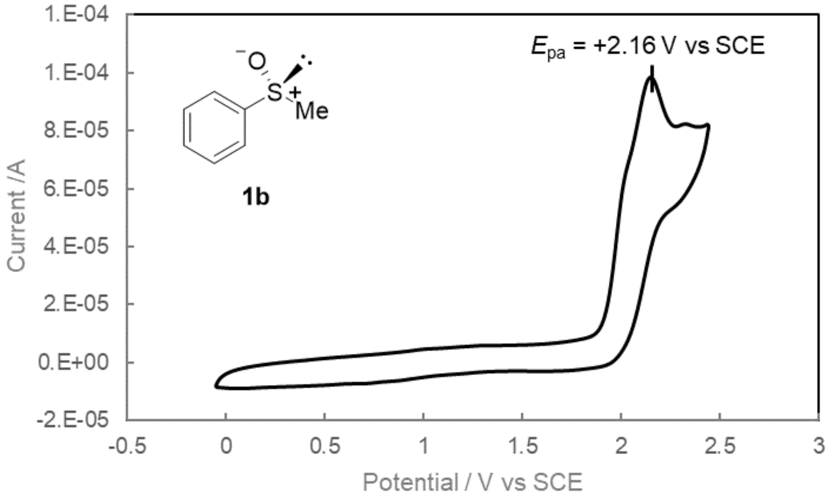

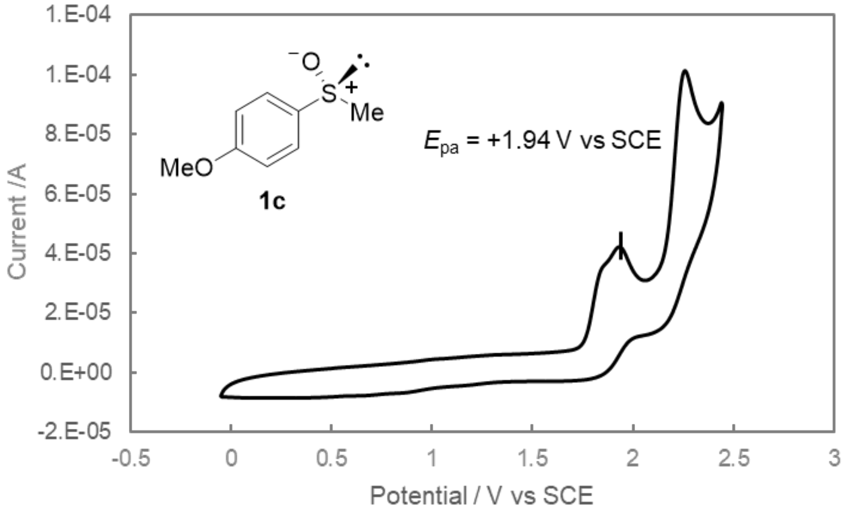

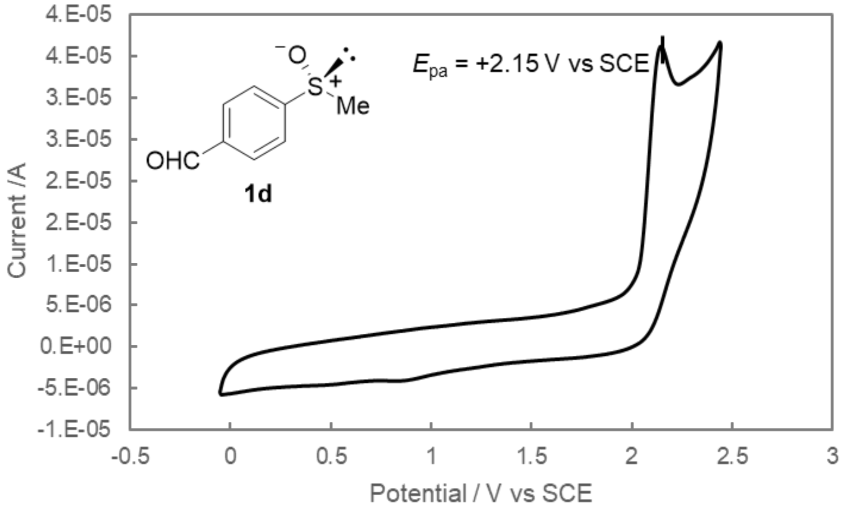
4.E-05
4.E-05
3.E-05
3.E-05
2.E-05
2.E-05
1.E-05
5.E-06
0.E+00
-5.E-06
-1.E-05
-0.5
0
0.5
1
1.5
2
2.5
3
Current /A
Potential / V vs SCE
E_{pa} = +2.15 V vs SCE
OHC
O
S
Me
1d

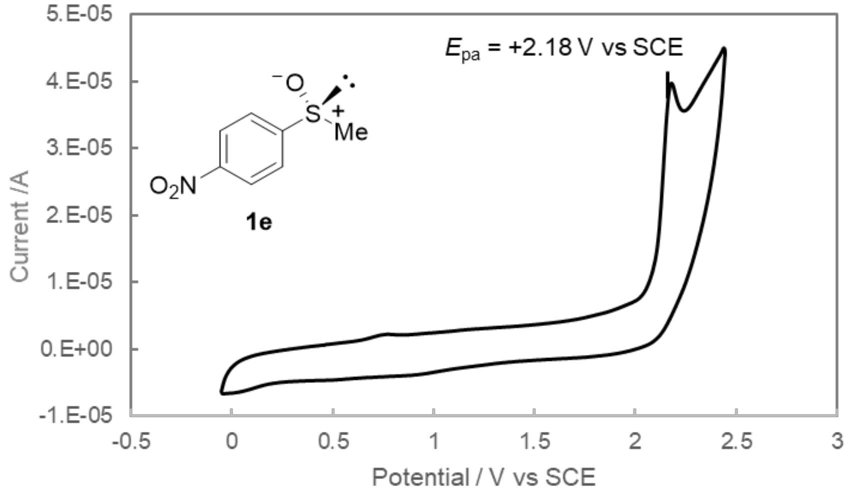
5.E-05
4.E-05
3.E-05
2.E-05
1.E-05
0.E+00
-1.E-05
-0.5
0
0.5
1
1.5
2
2.5
3
Current /A
Potential / V vs SCE
E_{pa} = +2.18 V vs SCE
O_2N
O
S
Me
1e

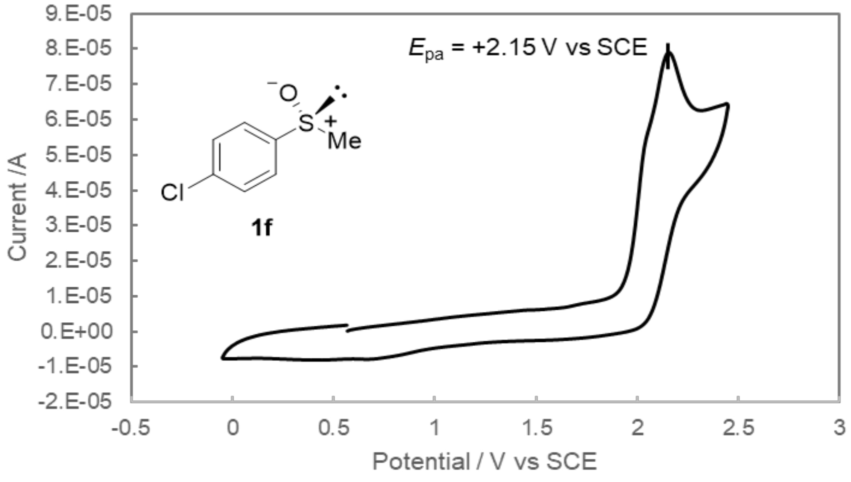
9.E-05
8.E-05
7.E-05
6.E-05
5.E-05
4.E-05
3.E-05
2.E-05
1.E-05
0.E+00
-1.E-05
-2.E-05
-0.5
0
0.5
1
1.5
2
2.5
3
Current /A
Potential / V vs SCE
E_{pa} = +2.15 V vs SCE
Cl
O
S
Me
1f

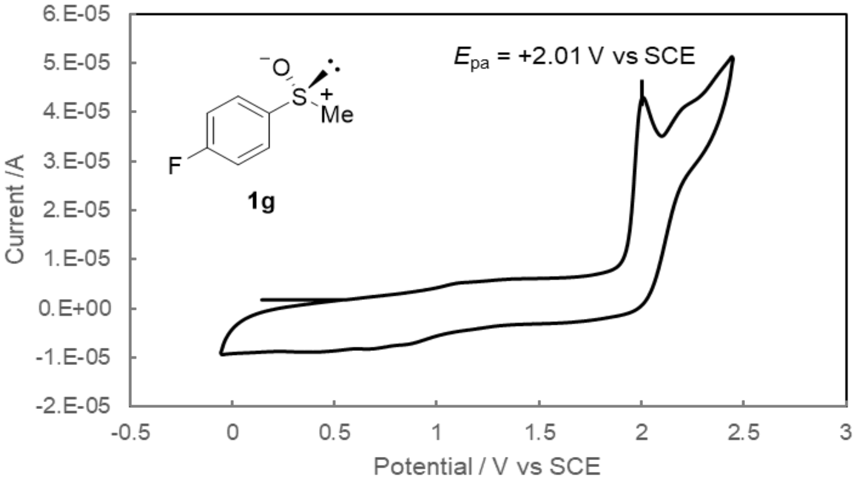

E_{pa} = +2.01 V vs SCE
1g
Current /A
Potential / V vs SCE

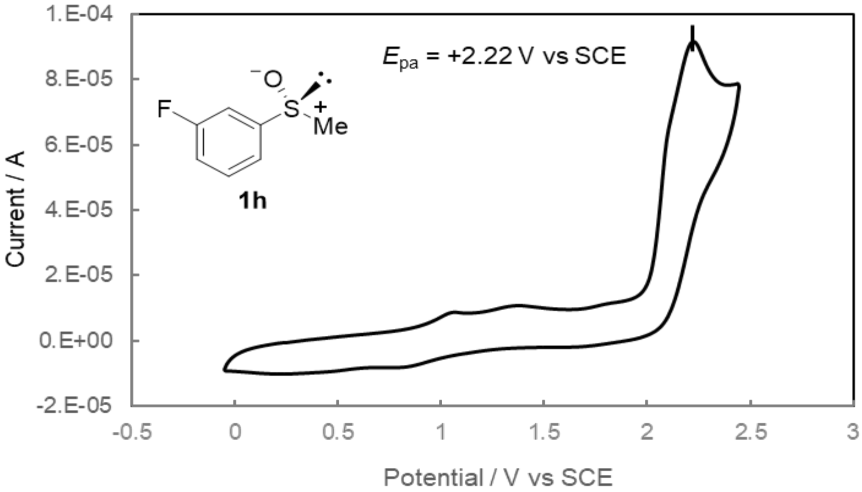

E_{pa} = +2.22 V vs SCE
1h
Current / A
Potential / V vs SCE

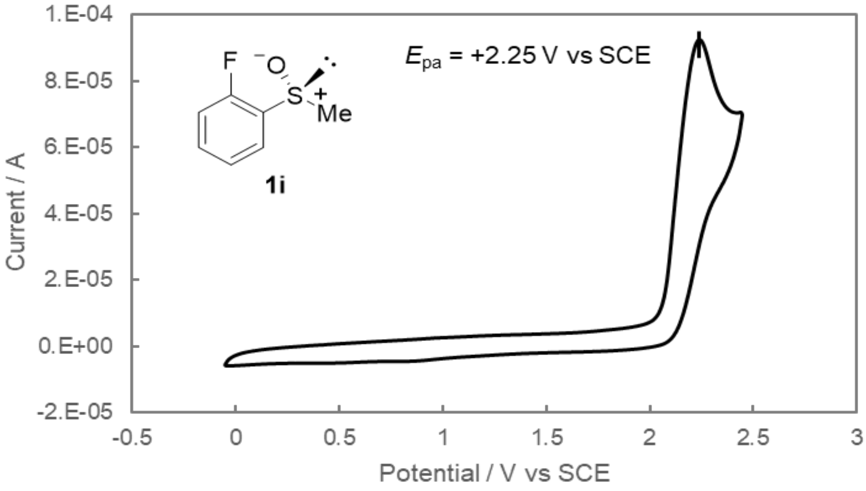

E_{pa} = +2.25 V vs SCE
1i
Current / A
Potential / V vs SCE

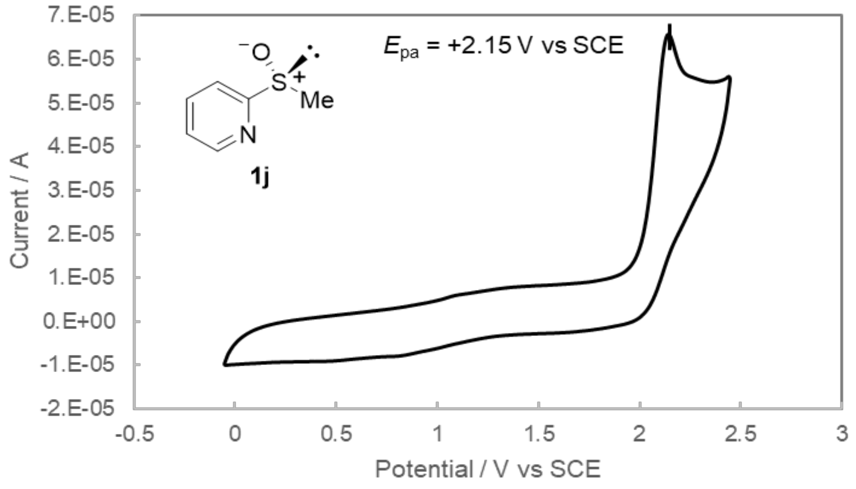

E_{pa} = +2.15 V vs SCE
Me
N
1j
Current / A
Potential / V vs SCE

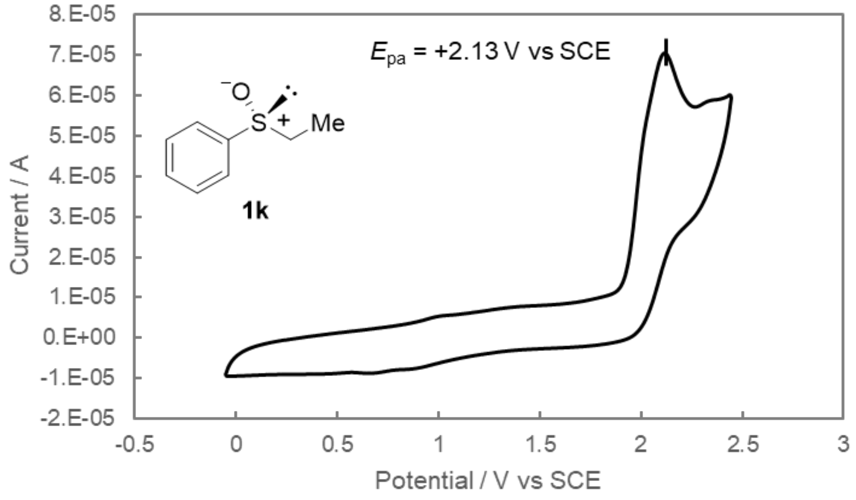

E_{pa} = +2.13 V vs SCE
Me
1k
Current / A
Potential / V vs SCE

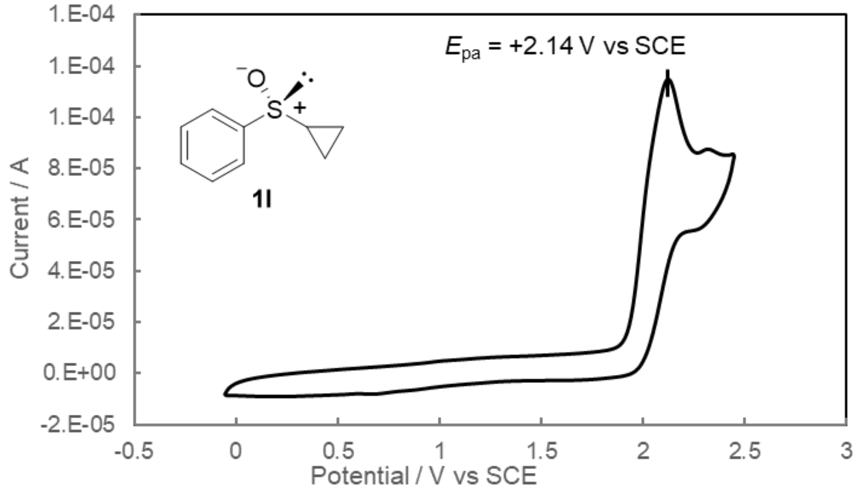

E_{pa} = +2.14 V vs SCE
1l
Current / A
Potential / V vs SCE

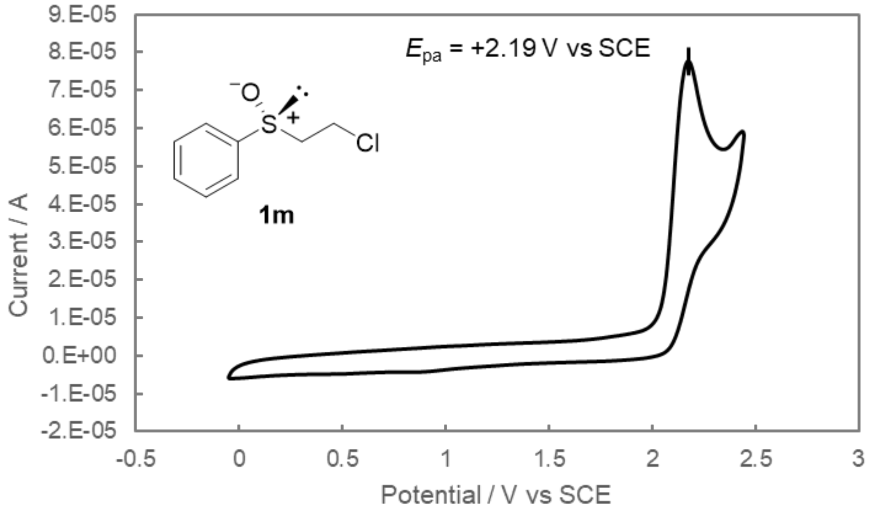
E_{pa} = +2.19 V vs SCE
O
S
+
Cl
1m
Current / A
Potential / V vs SCE
9.E-05
8.E-05
7.E-05
6.E-05
5.E-05
4.E-05
3.E-05
2.E-05
1.E-05
0.E+00
-1.E-05
-2.E-05
-0.5
0
0.5
1
1.5
2
2.5
3

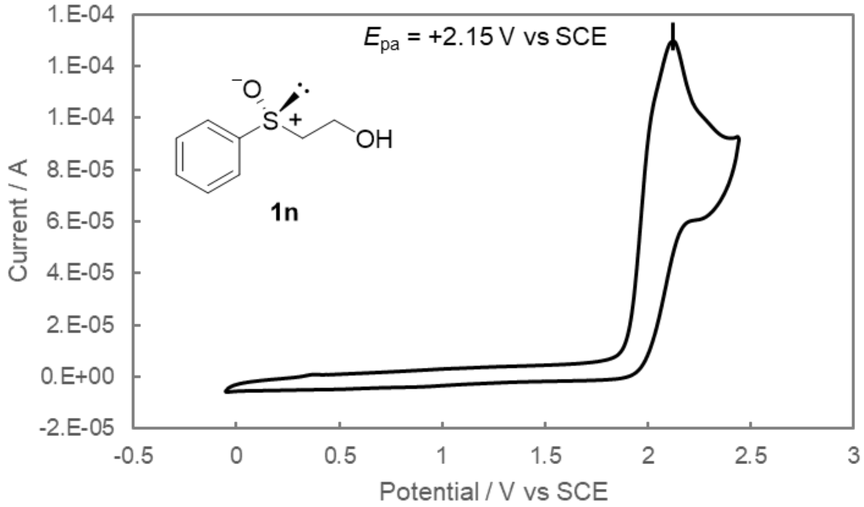
E_{pa} = +2.15 V vs SCE
O
S
+
OH
1n
Current / A
Potential / V vs SCE
1.E-04
1.E-04
1.E-04
8.E-05
6.E-05
4.E-05
2.E-05
0.E+00
-2.E-05
-0.5
0
0.5
1
1.5
2
2.5
3

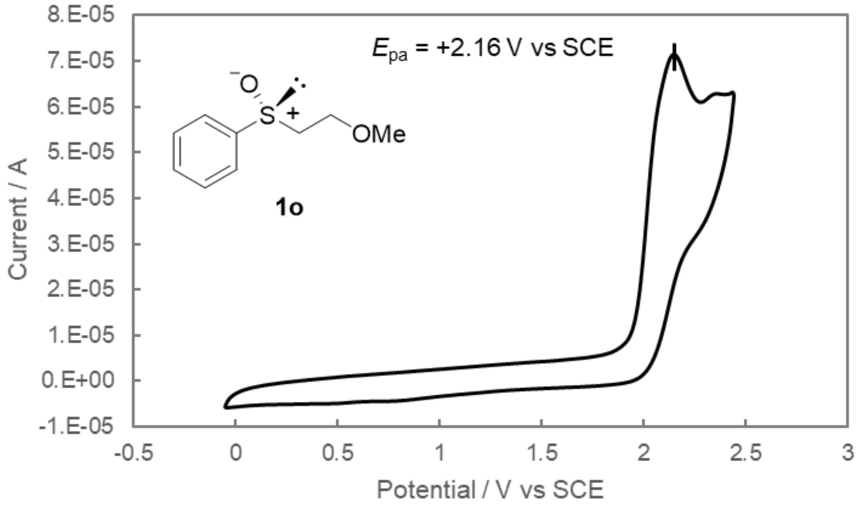
E_{pa} = +2.16 V vs SCE
O
S
+
OMe
1o
Current / A
Potential / V vs SCE
8.E-05
7.E-05
6.E-05
5.E-05
4.E-05
3.E-05
2.E-05
1.E-05
0.E+00
-1.E-05
-0.5
0
0.5
1
1.5
2
2.5
3

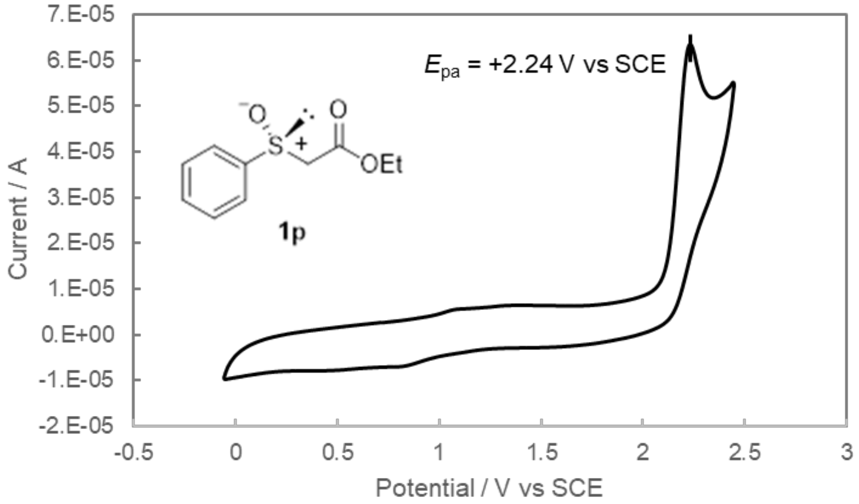

E_{pa} = +2.24 V vs SCE
OEt
1p
Current / A
Potential / V vs SCE

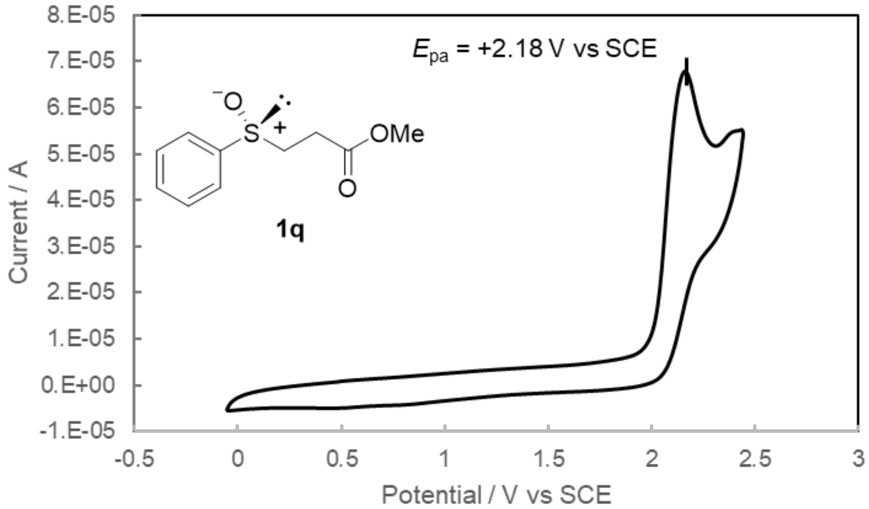

E_{pa} = +2.18 V vs SCE
OMe
1q
Current / A
Potential / V vs SCE

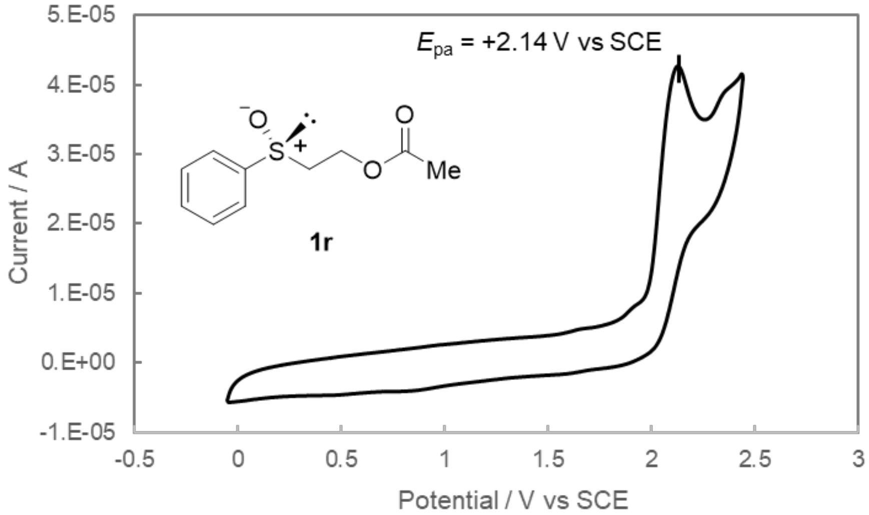

E_{pa} = +2.14 V vs SCE
Me
1r
Current / A
Potential / V vs SCE

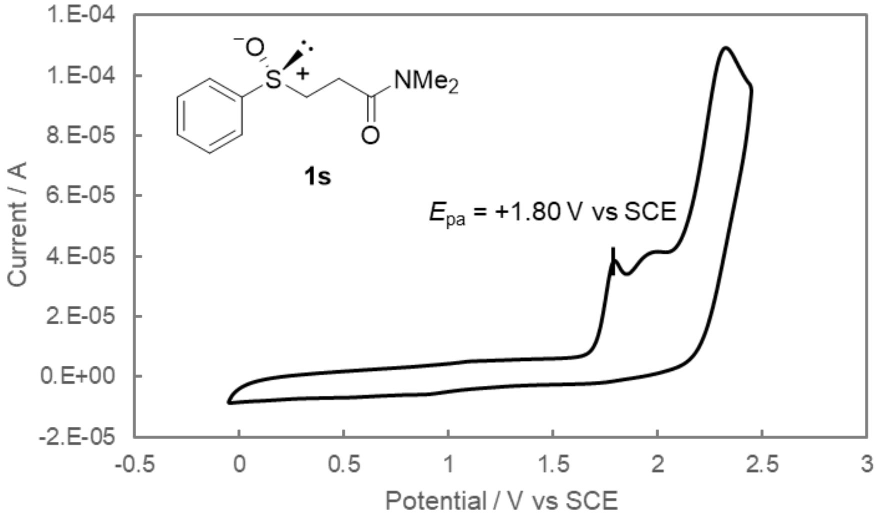
1.E-04
1.E-04
8.E-05
6.E-05
4.E-05
2.E-05
0.E+00
-2.E-05
Current / A
NMe2
1s
Epa = +1.80 V vs SCE
-0.5
0
0.5
1
1.5
2
2.5
3
Potential / V vs SCE

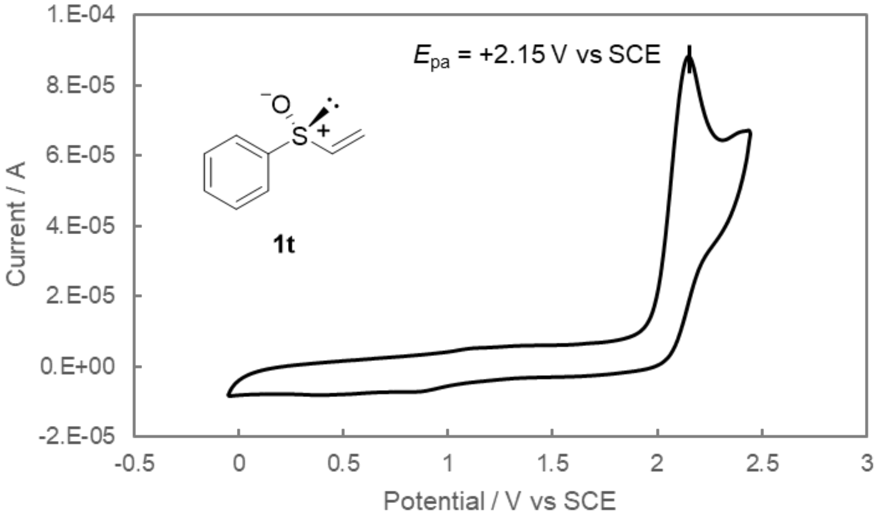
1.E-04
8.E-05
6.E-05
4.E-05
2.E-05
0.E+00
-2.E-05
Current / A
Epa = +2.15 V vs SCE
1t
-0.5
0
0.5
1
1.5
2
2.5
3
Potential / V vs SCE

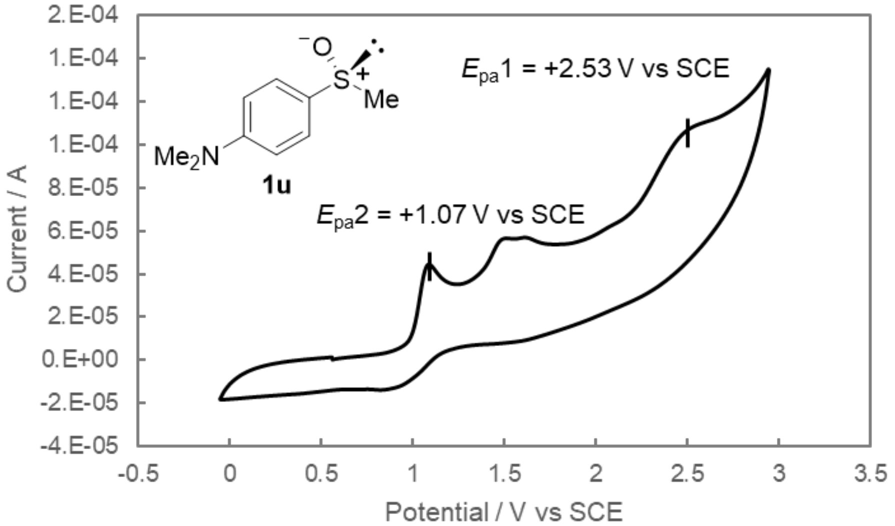
2.E-04
1.E-04
1.E-04
1.E-04
8.E-05
6.E-05
4.E-05
2.E-05
0.E+00
-2.E-05
-4.E-05
Current / A
Epa1 = +2.53 V vs SCE
Me
Me2N
1u
Epa2 = +1.07 V vs SCE
-0.5
0
0.5
1
1.5
2
2.5
3
3.5
Potential / V vs SCE

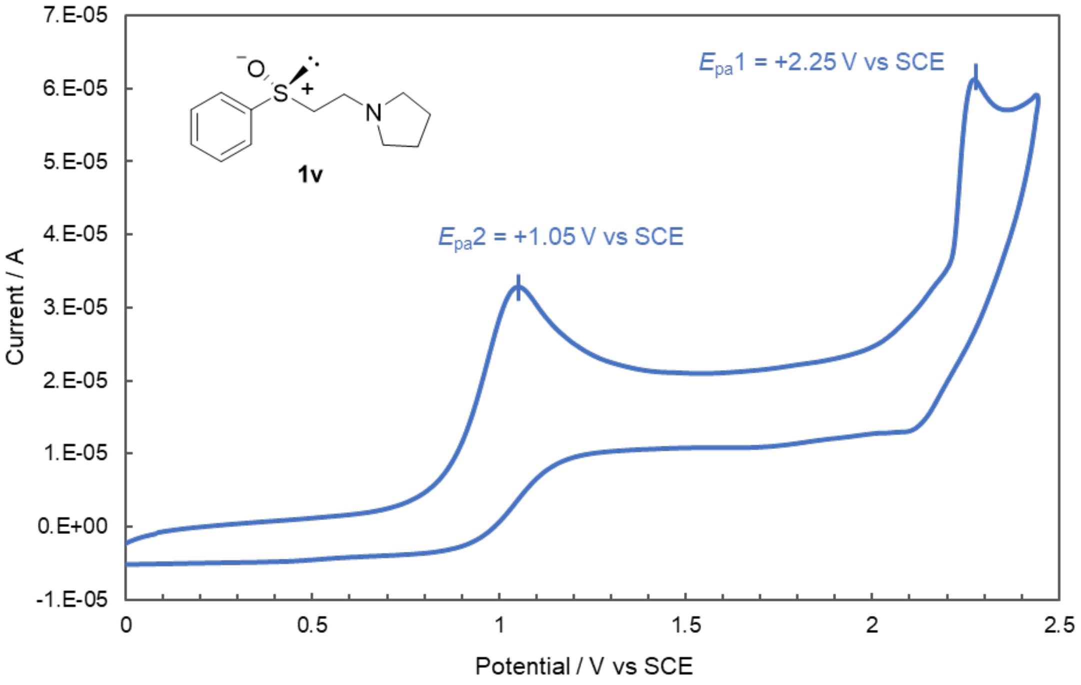

7.E-05
6.E-05
5.E-05
4.E-05
3.E-05
2.E-05
1.E-05
0.E+00
-1.E-05
Current / A
0
0.5
1
1.5
2
2.5
Potential / V vs SCE
1v
Epa1 = +2.25 V vs SCE
Epa2 = +1.05 V vs SCE

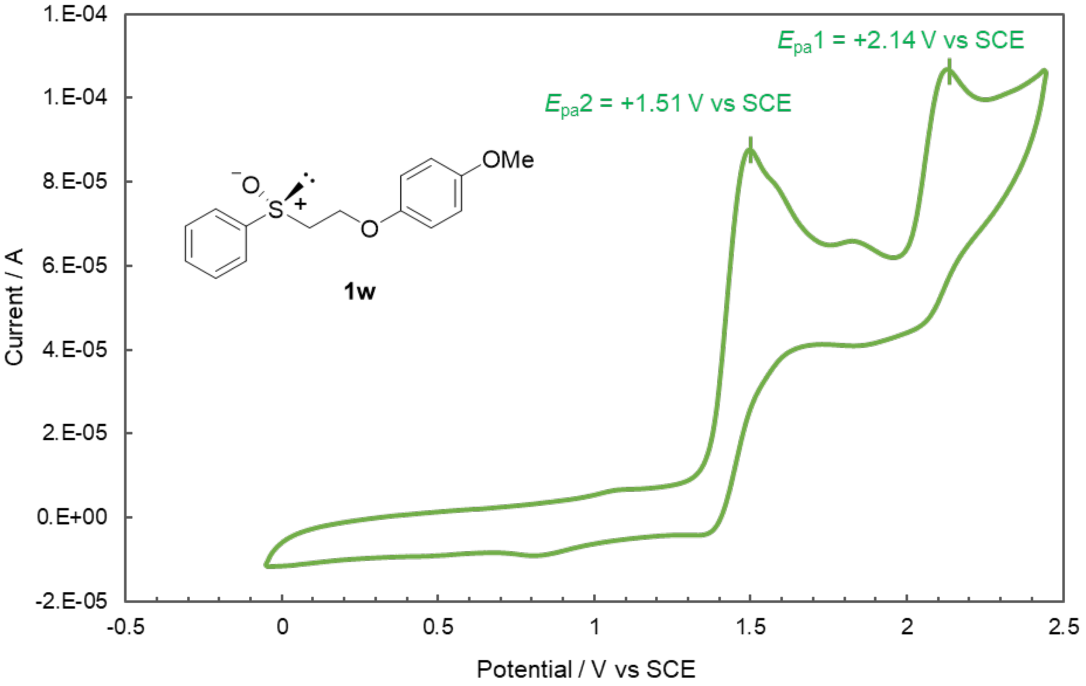

1.E-04
1.E-04
8.E-05
6.E-05
4.E-05
2.E-05
0.E+00
-2.E-05
Current / A
-0.5
0
0.5
1
1.5
2
2.5
Potential / V vs SCE
OMe
1w
Epa1 = +2.14 V vs SCE
Epa2 = +1.51 V vs SCE

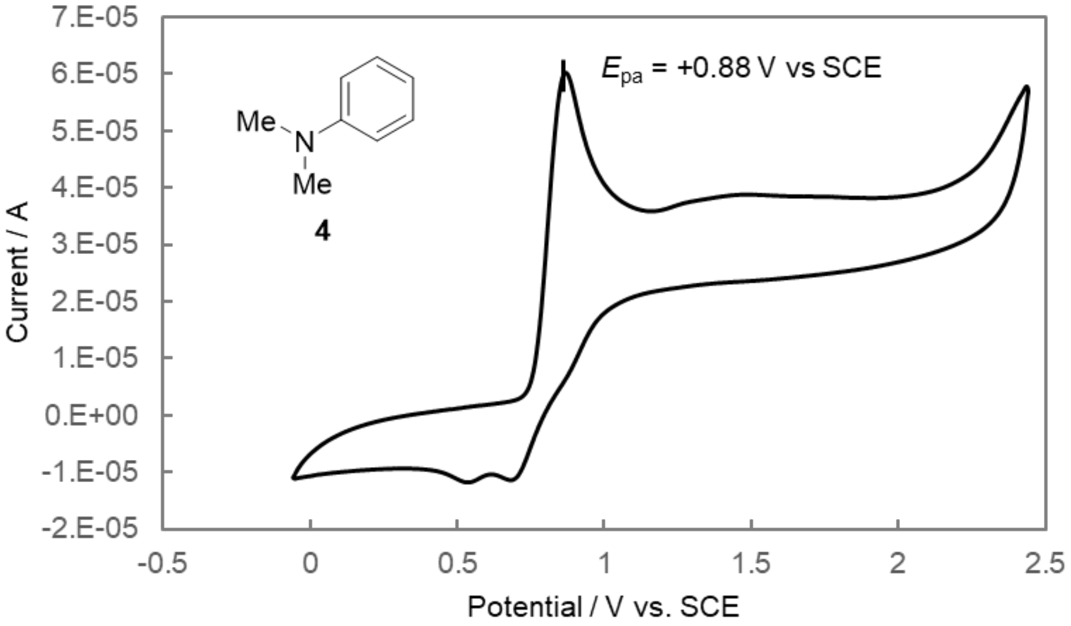

7.E-05
6.E-05
5.E-05
4.E-05
3.E-05
2.E-05
1.E-05
0.E+00
-1.E-05
-2.E-05
Current / A
-0.5
0
0.5
1
1.5
2
2.5
Potential / V vs. SCE
E_{pa} = +0.88 V vs SCE
Me
N
Me
4

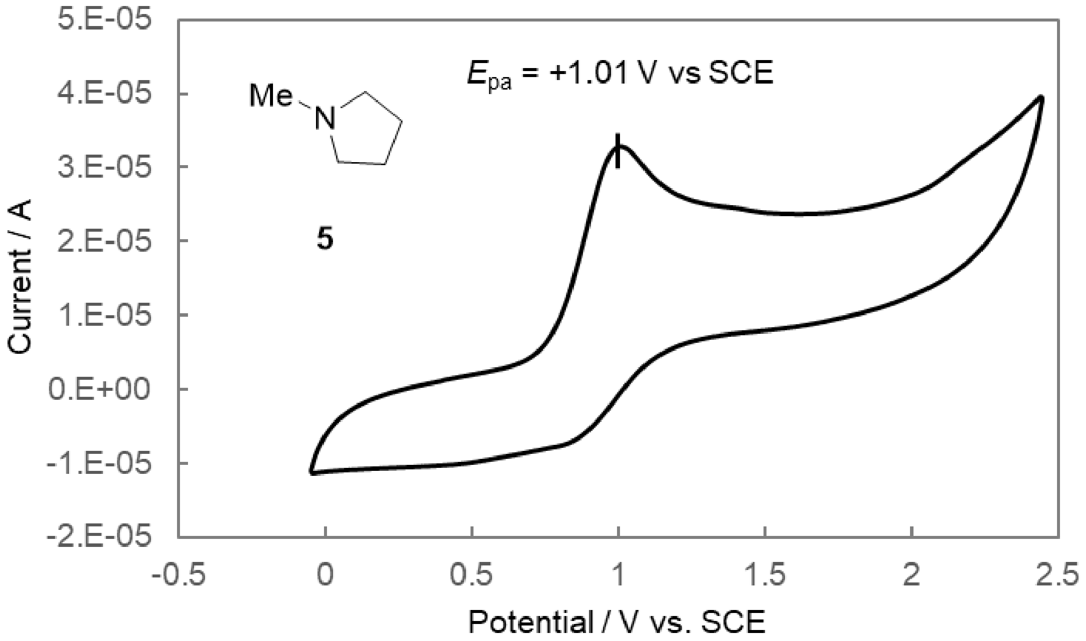

5.E-05
4.E-05
3.E-05
2.E-05
1.E-05
0.E+00
-1.E-05
-2.E-05
Current / A
-0.5
0
0.5
1
1.5
2
2.5
Potential / V vs. SCE
E_{pa} = +1.01 V vs SCE
Me
N
5

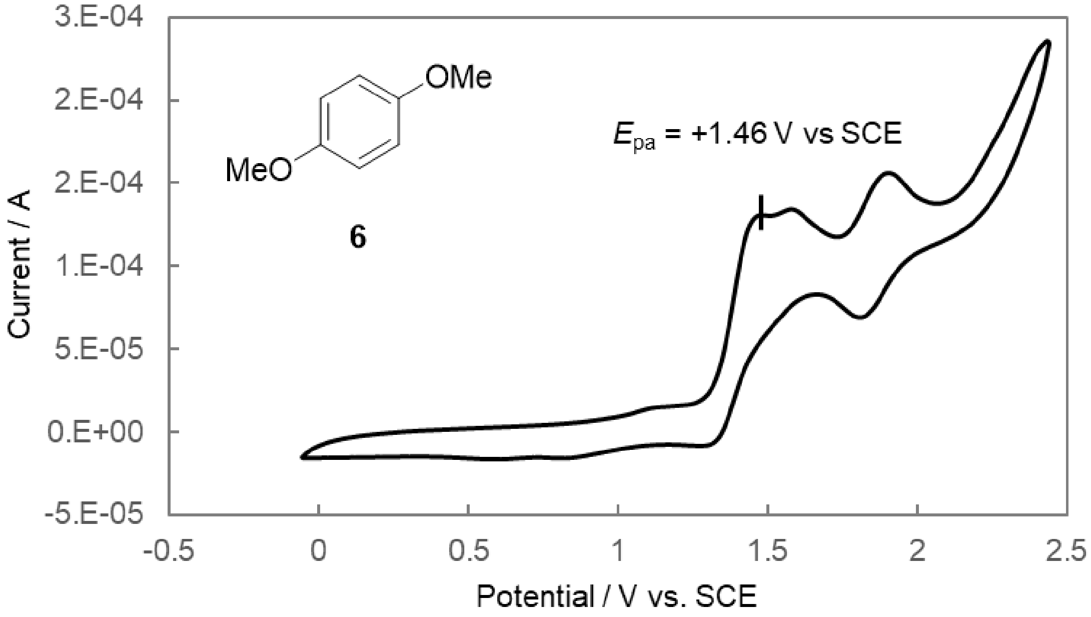
3.E-04
2.E-04
2.E-04
1.E-04
5.E-05
0.E+00
-5.E-05
Current / A
-0.5
0
0.5
1
1.5
2
2.5
Potential / V vs. SCE
OMe
MeO
6
E_{pa} = +1.46 V vs SCE

4. Computational details

DFT calculations were performed using the Gaussian-16 Revision A.03 quantum chemistry program package using M05-2X functional with the 6-311+G(3df,2p) basis set. The structures of (+)-**1a** and (+)-**1a**$^{•+}$ were optimized as a singlet for (+)-**1a** and doublet ground state for (+)-**1a**$^{•+}$.

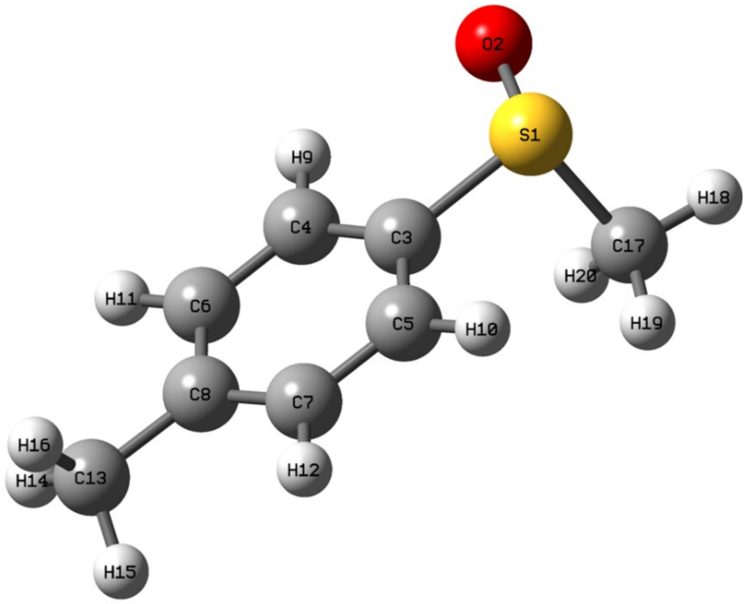

Figure S2. Optimized structure of (+)-**1a** (neutral).

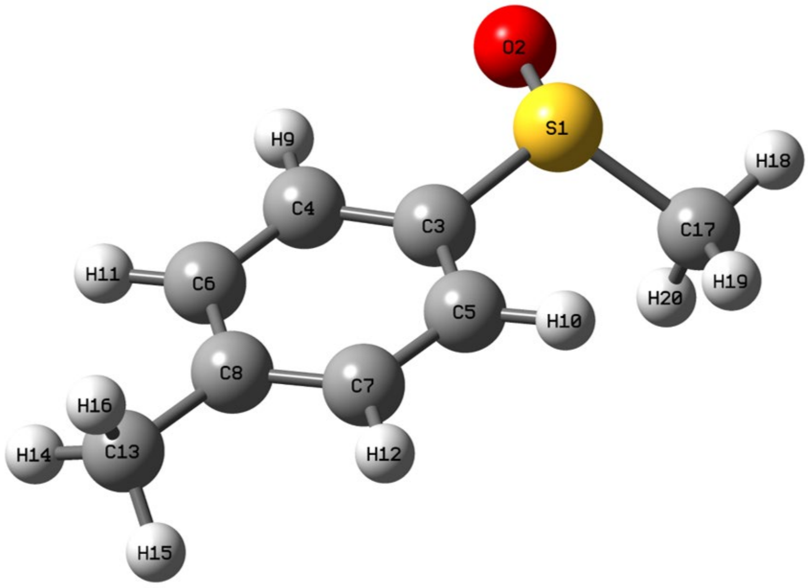

Figure S3. Optimized structure of (+)-**1a**$^{•+}$.

Table S2. XYZ coordinates of the optimized structure of (+)-**1a**.

E(RM052X) = -784.353978833 A.U

Item	Value	Threshold	Converged?
Maximum Force	0.000034	0.000450	YES
RMS Force	0.000009	0.000300	YES

Predicted change in Energy=-1.810524D-08

16	2.120779000	0.122897000	–0.430546000
8	2.609655000	–1.276711000	–0.304801000
6	0.344023000	0.073460000	–0.235236000
6	–0.250176000	–1.123416000	0.111855000
6	–0.413875000	1.212768000	–0.456749000
6	–1.628895000	–1.170597000	0.259419000
6	–1.786404000	1.150842000	–0.299266000
6	–2.412212000	–0.041049000	0.059662000
1	0.371712000	–1.994553000	0.252406000
1	0.061652000	2.137228000	–0.753459000
1	–2.103515000	–2.101765000	0.532508000
1	–2.384819000	2.035099000	–0.464331000
6	–3.907672000	–0.102531000	0.193580000
1	–4.213005000	–0.980382000	0.755499000
1	–4.289824000	0.780972000	0.698961000
1	–4.377237000	–0.151557000	–0.787711000
6	2.470887000	0.901029000	1.156743000
1	3.549242000	0.999582000	1.227006000
1	1.992197000	1.875153000	1.193296000
1	2.089846000	0.244527000	1.932932000

Table S3. XYZ coordinates of the optimized structure of (+)-**1a**$^{\bullet+}$.

E(UM052X) = -784.049198819 A.U.

Item	Value	Threshold	Converged?
Maximum Force	0.000021	0.000450	YES
RMS Force	0.000003	0.000300	YES

Predicted change in Energy=-8.333367D-09

16	–2.019738000	0.063083000	–0.326972000

8	–2.612252000	1.363771000	–0.061211000
6	–0.309982000	0.014933000	–0.133739000
6	0.358515000	1.220105000	0.090551000
6	0.367437000	–1.197550000	–0.314833000
6	1.729361000	1.185352000	0.200411000
6	1.735520000	–1.197164000	–0.200708000
6	2.438665000	–0.012489000	0.056259000
1	–0.194747000	2.139577000	0.198552000
1	–0.160291000	–2.111993000	–0.537948000
1	2.267833000	2.097436000	0.405828000
1	2.280436000	–2.121202000	–0.317277000
6	3.928078000	–0.032785000	0.144214000
1	4.310822000	0.858958000	0.628610000
1	4.272646000	–0.911165000	0.683383000
1	4.349319000	–0.080597000	–0.860722000
6	–2.773243000	–1.221576000	0.664155000
1	–3.834409000	–1.172804000	0.442295000
1	–2.352146000	–2.176452000	0.369912000
1	–2.562121000	–0.982102000	1.702194000

5. Chiral HPLC charts of 1a–y and their optical properties.

For **1a**

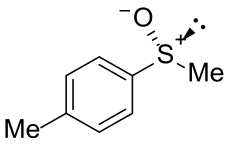

1a

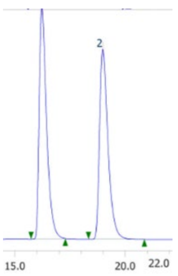

Conditions

Column: CHIRALPAK IG (4.6 mmϕ × 250 mm)

Eluent: 100% acetonitrile

Flow rate: 0.5 mL/min

Detection: 254 nm

Temperature: 23 ºC

Former peak (*R*): tR = 16.2 min; $[\alpha]_D^{20}$ +184.2. as 99% ee, (*c* 0.110, MeCN)

Latter peak (*S*): tR = 19.0 min; $[\alpha]_D^{20}$ –161.9 as 93% ee (*c* 0.150, MeCN)

For **1b**

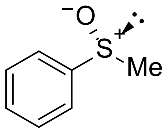

1b

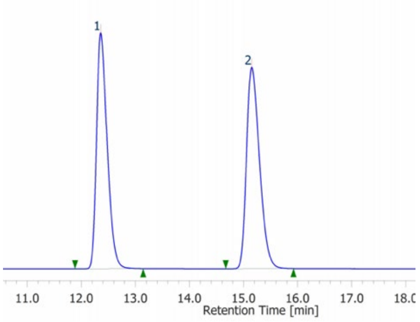

Conditions

Column: CHIRALPAK IH (4.6 mmϕ × 250 mm)

Eluent: 100% acetonitrile

Flow rate: 0.5 mL/min

Detection: 254 nm

Temperature: 23 ºC

Former peak (*R*): tR = 12.4 min, $[\alpha]_D^{20}$ +134.2 as 99% ee (*c* 0.200, MeCN)

Latter peak (*S*): tR = 15.1 min, $[\alpha]_D^{20}$ –135.3 as 98% ee (*c* 0.210, MeCN)

For **1c**

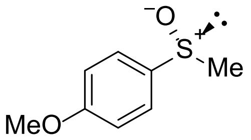

1c

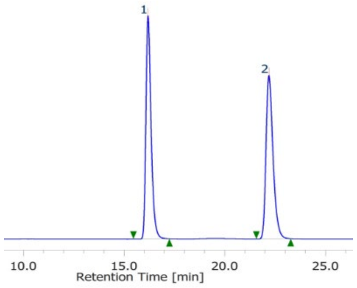

<u>Conditions</u>

Column: CHIRALPAK IH (4.6 mmϕ × 250 mm)

Eluent: 100% acetonitrile

Flow rate: 0.5 mL/min

Detection: 254 nm

Temperature: 23 ºC

Former peak (*R*): tR = 16.6 min; $[\alpha]_D^{20}$ +124.7 as 99% ee (*c* 0.480, MeCN)

Latter peak (*S*): tR = 23.1 min; $[\alpha]_D^{20}$ –124.9 as 97% ee (*c* 0.490, MeCN)

For **1d**

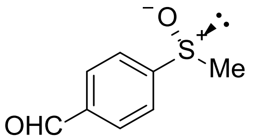

1d

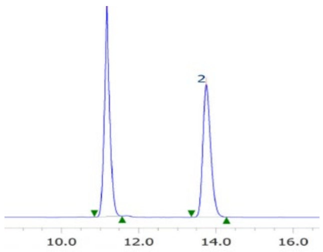

<u>Conditions</u>

Column: CHIRALPAK IH (4.6 mmϕ × 250 mm)

Eluent: 100% acetonitrile

Flow rate: 0.5 mL/min

Detection: 254 nm

Temperature: 23 ºC

Former peak (*R*): tR = 11.2 min; $[\alpha]_D^{20}$ +41.1 as 94% ee, (*c* 0.310, MeCN)

Latter peak (*S*): tR = 13.8 min; $[\alpha]_D^{20}$ –41.6 as 98% ee (*c* 0.290, MeCN)

For **1e**

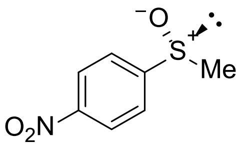

1e

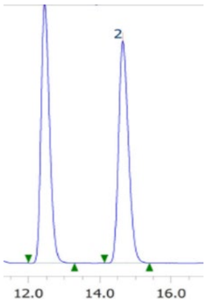

<u>Conditions</u>

Column: CHIRALPAK IH (4.6 mmϕ × 250 mm)

Eluent: 100% acetonitrile

Flow rate: 0.5 mL/min

Detection: 254 nm

Temperature: 23 ºC

Former peak (*R*): tR = 12.5 min; $[\alpha]_D^{20}$ –107.4 as 99% ee, (*c* 0.260, MeCN)

Latter peak (*S*): tR = 14.6 min; $[\alpha]_D^{20}$ +106.7 as 96% ee (*c* 0.350, MeCN)

For **1f**

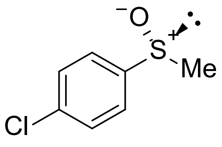

1f

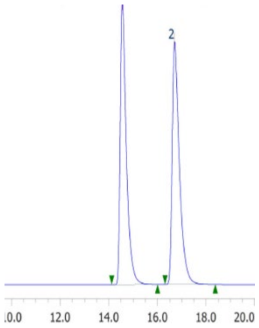

<u>Conditions</u>

Column: CHIRALPAK IH (4.6 mmϕ × 250 mm)

Eluent: 100% acetonitrile

Flow rate: 0.5 mL/min

Detection: 254 nm

Temperature: 23 ºC

Former peak (*R*): tR = 14.6 min; $[\alpha]_D^{20}$ +98.2 as 99% ee, (*c* 0.320, MeCN)

Latter peak (*S*): tR = 16.7 min; $[\alpha]_D^{20}$ –95.7 as 99% ee (*c* 0.300, MeCN)

For **1g**

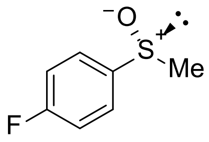

1g

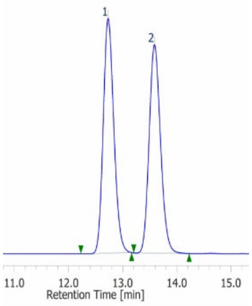

Conditions

Column: CHIRALPAK IH (4.6 mmϕ × 250 mm)

Eluent: 100% acetonitrile

Flow rate: 0.5 mL/min

Detection: 254 nm

Temperature: 23 ºC

Former peak (*R*): tR = 12.7 min; $[\alpha]_D^{20}$ +121.7 as 99% ee, (*c* 0.080, MeCN)

Latter peak (*S*): tR = 13.6 min; $[\alpha]_D^{20}$ –105.2 as 96% ee (*c* 0.165, MeCN)

For **1h**

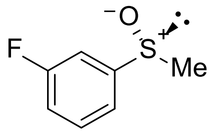

1h

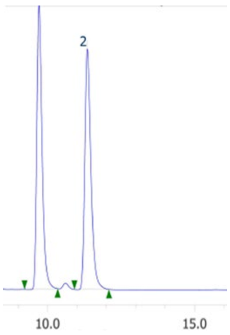

Conditions

Column: CHIRALPAK IG (4.6 mmϕ × 250 mm)

Eluent: 100% acetonitrile

Flow rate: 0.5 mL/min

Detection: 254 nm

Temperature: 23 ºC

Former peak (*R*): tR = 9.7 min; $[\alpha]_D^{20}$ +110.8 as 99% ee, (*c* 0.240, MeCN)

Latter peak (*S*): tR = 11.4 min; $[\alpha]_D^{20}$ –111.7 as 98% ee (*c* 0.200, MeCN)

For **1i**

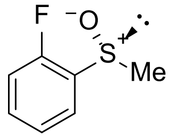

1i

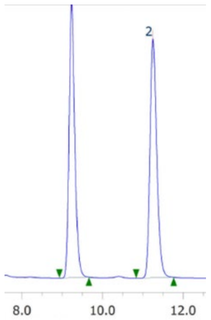

<u>Conditions</u>

Column: CHIRALPAK IH (4.6 mmϕ × 250 mm)

Eluent: 100% acetonitrile

Flow rate: 0.5 mL/min

Detection: 254 nm

Temperature: 23 ºC

Former peak (*R*): tR = 9.2 min; $[\alpha]_D^{20}$ +162.2 as 99% ee (*c* 0.210, MeCN)

Latter peak (*S*): tR = 11.2 min; $[\alpha]_D^{20}$ –162.5 as 98% ee (*c* 0.130, MeCN)

For **1j**

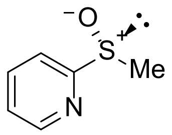

1j

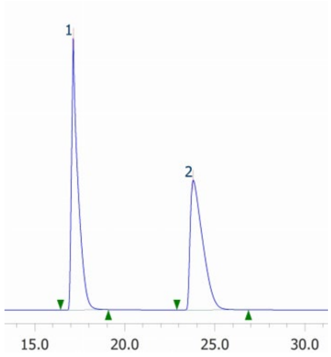

<u>Conditions</u>

Column: CHIRALPAK IH (4.6 mmϕ × 250 mm)

Eluent: 40% *i*-PrOH in hexane,

Flow rate: 0.5 mL/min

Detection: 254 nm

Temperature: 23 ºC

Former peak (*R*): tR = 17.1 min; $[\alpha]_D^{20}$ +59.3 as 99% ee (*c* 0.110, MeCN)

Latter peak (*S*): tR = 23.8 min; $[\alpha]_D^{20}$ –58.9 as 99% ee (*c* 1.320, MeCN)

For **1k**

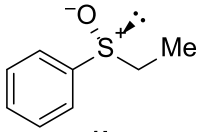

1k

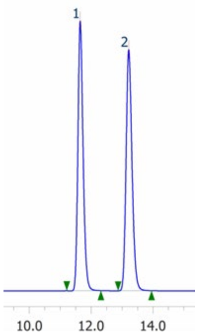

Conditions

Column: CHIRALPAK IH (4.6 mmϕ × 250 mm)

Eluent: 100% acetonitrile

Flow rate: 0.5 mL/min

Detection: 254 nm

Temperature: 23 ºC,

Former peak (*R*): tR = 11.6 min $[\alpha]_D^{20}$ +142.1 as 99% ee (*c* 0.150, MeCN)

Latter peak (*S*): tR = 13.2 min $[\alpha]_D^{20}$ –142.9 as 96% ee (*c* 0.220, MeCN)

For **1l**

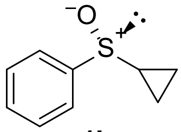

1l

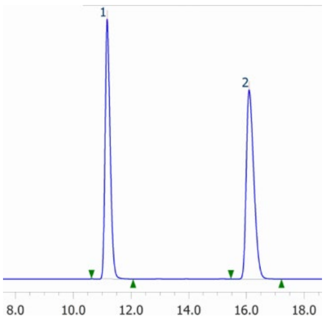

Conditions

Column: CHIRALPAK IH (4.6 mmϕ × 250 mm)

Eluent: 100% acetonitrile

Flow rate: 0.5 mL/min

Detection: 254 nm

Temperature: 23 ºC

Former peak (*R*): tR = 11.2 min; $[\alpha]_D^{20}$ +194.7 as 97% ee (*c* 0.160, MeCN)

Latter peak (*S*): tR = 16.1 min; $[\alpha]_D^{20}$ –195.6 as 99% ee (*c* 0.160, MeCN)

For **1m**

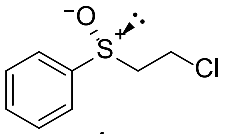

1m

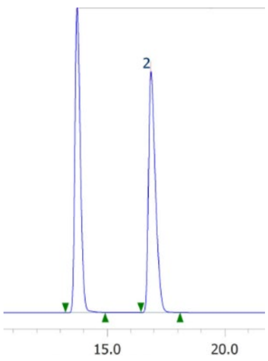

<u>Conditions</u>

Column: CHIRALPAK IG (4.6 mmϕ × 250 mm)

Eluent: 100% acetonitrile

Flow rate: 0.5 mL/min

Detection: 254 nm

Temperature: 23 ºC

Former peak (*R*): tR = 14.8 min; $[\alpha]_D^{20}$ +102.7 as 99% ee (*c* 0.310, MeCN)

Latter peak (*S*): tR = 18.3 min; $[\alpha]_D^{20}$ –104.5 as 99% ee (*c* 0.520, MeCN)

For **1n**

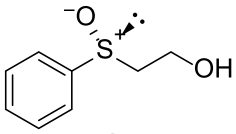

1n

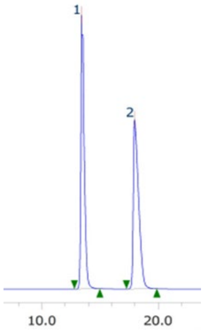

<u>Conditions</u>

Column: CHIRALPAK IF (4.6 mmϕ × 250 mm)

Eluent: 100% acetonitrile

Flow rate: 0.5 mL/min

Detection: 254 nm

Temperature: 23 ºC

Former peak (*R*): tR = 13.4 min; $[\alpha]_D^{20}$ +225.6 as 98% ee (*c* 0.410, MeCN)

Latter peak (*S*): tR = 18.0 min; $[\alpha]_D^{20}$ –227.1 as 97% ee (*c* 0.310, MeCN)

For **1o**

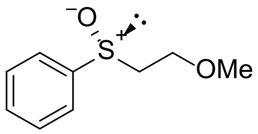

1o

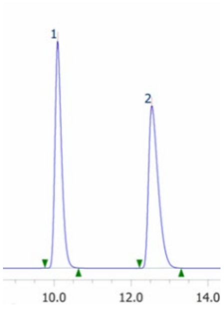

<u>Conditions</u>

Column: CHIRALPAK ID (4.6 mmϕ × 250 mm)

Eluent: 100% acetonitrile

Flow rate: 0.5 mL/min

Detection: 254 nm

Temperature: 23 ºC

Former peak (*R*): tR = 10.1 min; $[\alpha]_D^{20}$ +209.0 as 99% ee (*c* 0.130, MeCN)

Latter peak (*S*): tR = 12.5 min; $[\alpha]_D^{20}$ –207.2 as 99% ee (*c* 0.150, MeCN)

For **1p**

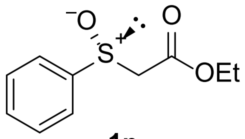

1p

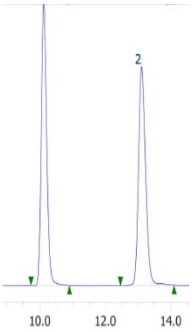

<u>Conditions</u>

Column: CHIRALPAK IG (4.6 mmϕ × 250 mm)

Eluent: 100% acetonitrile

Flow rate: 0.5 mL/min

Detection: 254 nm

Temperature: 23 ºC

Former peak (*R*): tR = 10.1 min; $[\alpha]_D^{20}$ –204.4 as 99% ee (*c* 0.360, MeCN).

Latter peak (*S*): tR = 13.1 min; $[\alpha]_D^{20}$ +183.8 as 99% ee (*c* 0.290, MeCN)

For **1q**

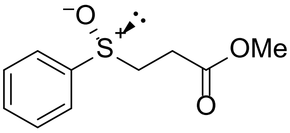

1q

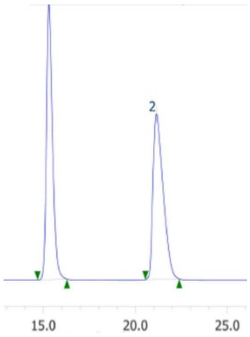

<u>Conditions</u>

Column: CHIRALPAK IF (4.6 mmϕ × 250 mm)

Eluent: 100% acetonitrile

Flow rate: 0.5 mL/min

Detection: 254 nm

Temperature: 23 ºC

Former peak (*R*): tR = 15.3 min; $[\alpha]_D^{20}$ +121.1 as 99% ee (*c* 0.410, MeCN)

Latter peak (*S*): tR = 21.1 min; $[\alpha]_D^{20}$ –124.6 as 94% ee (*c* 0.230, MeCN)

For **1r**

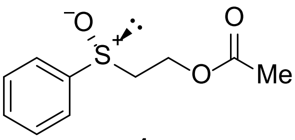

1r

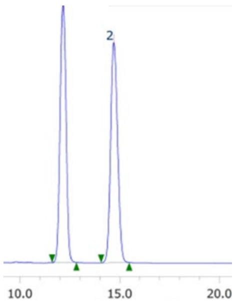

<u>Conditions</u>

Column: CHIRALPAK IF (4.6 mmϕ × 250 mm)

Eluent: 100% acetonitrile,

Flow rate: 0.5 mL/min

Detection: 254 nm

Temperature: 23 ºC

Former peak (*R*): tR = 12.1 min; $[\alpha]_D^{20}$ –123.3 as 99% ee (*c* 0.110, MeCN)

Latter peak (*S*): tR = 14.7 min; $[\alpha]_D^{20}$ +121.7 as 98% ee (*c* 0.170, MeCN)

For **1s**

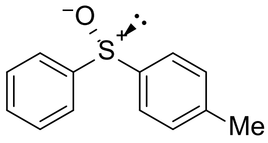

1s

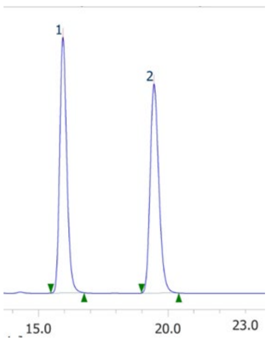

<u>Conditions</u>

Column: CHIRALPAK IG (4.6 mmϕ × 250 mm)

Eluent: 100% acetonitrile

Flow rate: 0.5 mL/min

Detection: 254 nm

Temperature: 23 ºC

Former peak (*S*): tR = 15.9 min; $[\alpha]_D^{20}$ –13.8 as 99% ee (*c* 0.250, MeCN)

Latter peak (*R*): tR = 19.5 min; $[\alpha]_D^{20}$ +18.8 as 99% ee (*c* 0.250, MeCN)

For **1t**

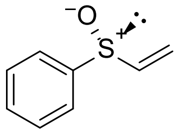

1t

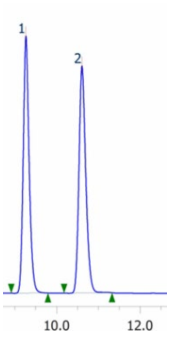

<u>Conditions</u>

Column: CHIRALPAK IH (4.6 mmϕ × 250 mm)

Eluent: 100% acetonitrile

Flow rate: 0.5 mL/min

Detection: 254 nm

Temperature: 23 ºC

Former peak (*R*): tR = 9.3 min; $[\alpha]_D^{20}$ +421.0 as 99% ee (*c* 0.310, MeCN)

Latter peak (*S*): tR = 10.6 min; $[\alpha]_D^{20}$ –418.5 as 99% ee (*c* 0.190, MeCN)

For **1u**

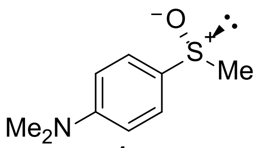

1u

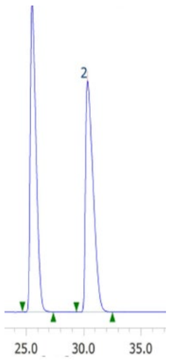

<u>Conditions</u>

Column: CHIRALPAK IA (4.6 mmϕ × 250 mm)

Eluent: 100% acetonitrile

Flow rate: 0.5 mL/min

Detection: 254 nm

Temperature: 23 ºC

Former peak (*R*): tR = 25.5 min; $[\alpha]_D^{20}$ -91.6 as 99% ee (*c* 0.130, MeCN)

Latter peak (*S*): tR = 30.3 min; $[\alpha]_D^{20}$ –102.9 as 99% ee (*c* 0.060, MeCN)

For **1v**

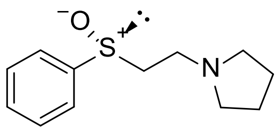

1v

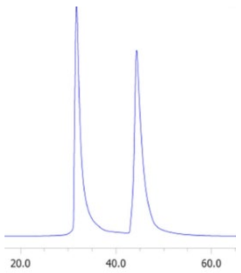

<u>Conditions</u>

Column: CHIRALPAK IG (4.6 mmϕ × 250 mm)

Eluent: 100% acetonitrile

Flow rate: 0.5 mL/min

Detection: 254 nm

Temperature: 23 ºC

Former peak: tR = 31.7 min; $[\alpha]_D^{20}$ +133.8 as 99% ee (*c* 0.090, MeCN).

Latter peak: tR = 43.3 min; $[\alpha]_D^{20}$ –128.8 as 99% ee (*c* 0.030, MeCN)

For **1w**

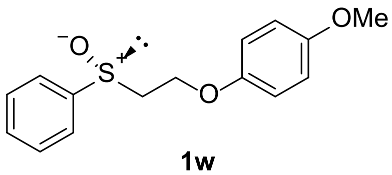

1w

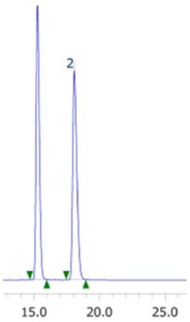

<u>Conditions</u>

Column: CHIRALPAK IA (4.6 mmϕ × 250 mm)

Eluent: 100% acetonitrile

Flow rate: 0.5 mL/min

Detection: 254 nm

Temperature: 23 ºC

Former peak: tR = 15,2 min; $[\alpha]_D^{20}$ +91.5 as 99% ee (*c* 0.180, MeCN).

Latter peak: tR = 17.9 min; $[\alpha]_D^{20}$ –91.0 as 95% ee (*c* 0.250, MeCN)

6. Optical purity of (+)-1a-w.

For **1a**

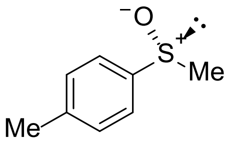

(+)-**1a**

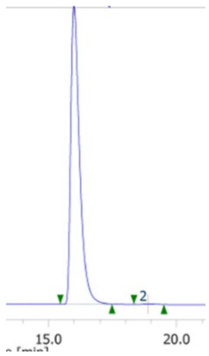

HPLC (CHIRALPAK IG, acetonitrile, flow rate = 0.5 mL/min, l = 254 nm) tR = 16.0 min, 99.8% (major), 18.9 min, 0.2% (minor), 99.6% ee

For **1b**

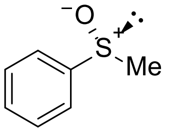

(+)-**1b**

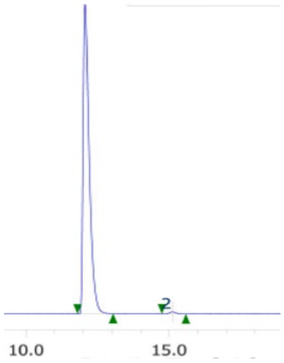

HPLC (CHIRALPAK IH, acetonitrile, flow rate = 0.5 mL/min, l = 254 nm) tR = 12.1 min, 99.3% (major), 15.1 min, 0.7% (minor), 98.6 %ee

For **1c**

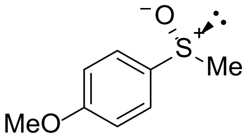

(+)-**1c**

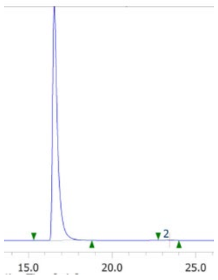

HPLC (CHIRALPAK IH, acetonitrile, flow rate = 0.5 mL/min, l = 254 nm) tR = 16.5 min, 99.8% (major), 23.4 min, 0.2% (minor), 99.6 %ee

For **1d**

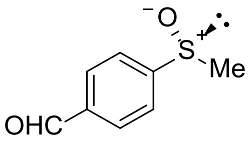

(+)-**1d**

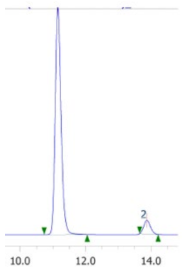

HPLC (CHIRALPAK IH, acetonitrile, flow rate = 0.5 mL/min, l = 254 nm) tR = 11.2 min, 93.5% (major), 13.9 min, 6.5% (minor), 87.0% ee

For **1e**

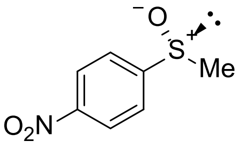

(+)-**1e**

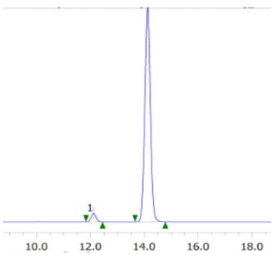

HPLC (CHIRALPAK IH, acetonitrile, flow rate = 0.5 mL/min, l = 254 nm) tR = 12.1 min, 3.2% (minor), 14.1 min, 96.8% (major), 93.6% ee

For **1f**

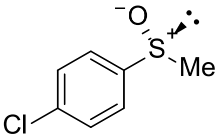

(+)-**1f**

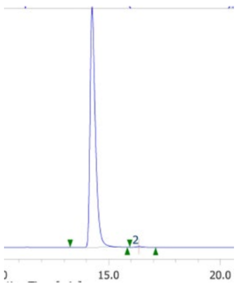

HPLC (CHIRALPAK IH, acetonitrile, flow rate = 0.5 mL/min, l = 254 nm) tR = 14.3 min, 99.6% (major), 16.4 min, 0.4% (minor), 99.2% ee

For **1g**

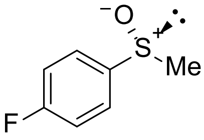

(+)-**1g**

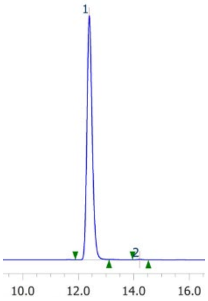

HPLC (CHIRALPAK IH, acetonitrile, flow rate = 0.5 mL/min, l = 254 nm) tR = 12.4 min, 99.8% (major), 14.2 min, 0.2% (minor), 99.6% ee

For **1h**

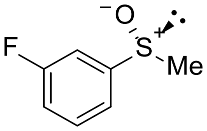

(+)-**1h**

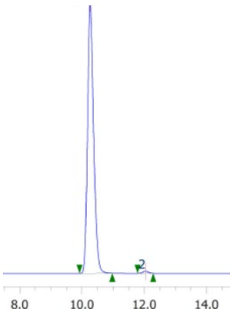

HPLC (CHIRALPAK IG, acetonitrile, flow rate = 0.5 mL/min, l = 254 nm) tR = 10.3 min, 99.3% (major), 12.0 min, 0.7% (minor), 98.6% ee

For **1i**

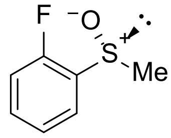

(+)-**1i**

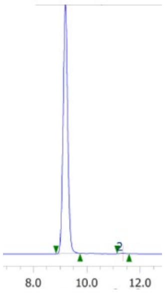

HPLC (CHIRALPAK IH, acetonitrile, flow rate = 0.5 mL/min, l = 254 nm) tR = 9.2 min, 99.8% (major), 11.4 min, 0.2% (minor), 99.6% ee

For **1j**

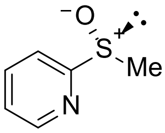

(+)-**1j**

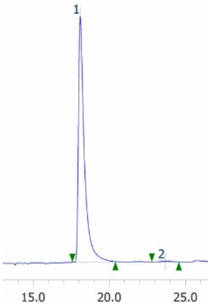

HPLC (CHIRALPAK IH, acetonitrile, flow rate = 0.5 mL/min, l = 254 nm) tR = 18.1 min, 99.5% (major), 23.7 min, 0.5% (minor), 99.0% ee

For **1k**

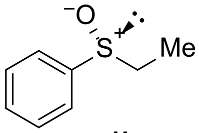

(+)-**1k**

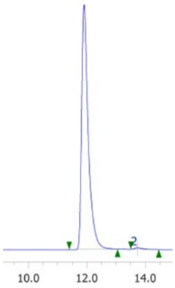

HPLC (CHIRALPAK IH, acetonitrile, flow rate = 0.5 mL/min, l = 254 nm) tR = 11.9 min, 99.3% (major), 13.7 min, 0.7% (minor), 98.6% ee

For **1l**

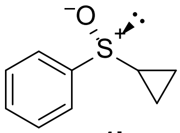

(+)-**1l**

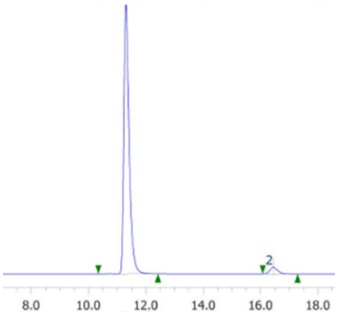

HPLC (CHIRALPAK IH, acetonitrile, flow rate = 0.5 mL/min, l = 254 nm) tR = 11.3 min, 96.6% (major), 16.4 min, 3.4% (minor), 93.2% ee

For **1m**

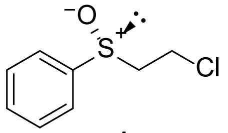

(+)-**1m**

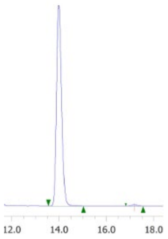

HPLC (CHIRALPAK IG, acetonitrile, flow rate = 0.5 mL/min, l = 254 nm) tR = 14.0 min, 99.3% (major), 17.2 min, 0.7% (minor), 98.6% ee

For **1n**

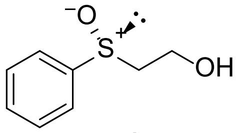

(+)-**1n**

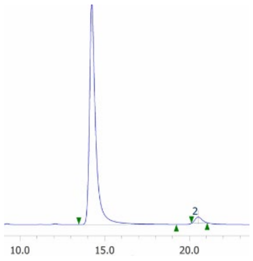

HPLC (CHIRALPAK IF, acetonitrile, flow rate = 0.5 mL/min, l = 254 nm) tR = 14.2 min, 97.1% (major), 20.5 min, 2.8% (minor), 94.3% ee

For **1o**

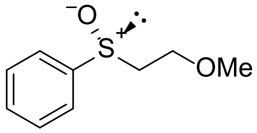

(+)-**1o**

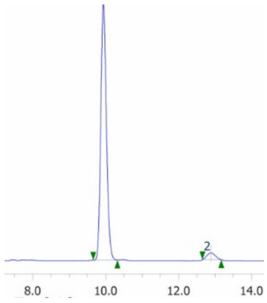

HPLC (CHIRALPAK ID, acetonitrile, flow rate = 0.5 mL/min, l = 254 nm) tR = 9.9 min, 96.1% (major), 12.8 min, 3.8% (minor), 92.3% ee

For **1p**

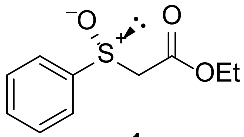

(+)-**1p**

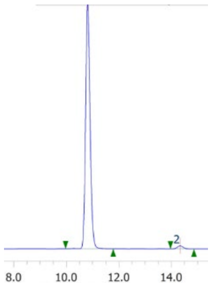

HPLC (CHIRALPAK IG, acetonitrile, flow rate = 0.5 mL/min, l = 254 nm) tR = 10.8 min, 98.8% (major), 14.3 min, 1.2% (minor), 97.6% ee

For **1q**

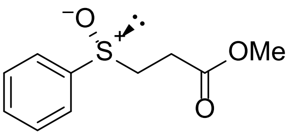

(+)-**1q**

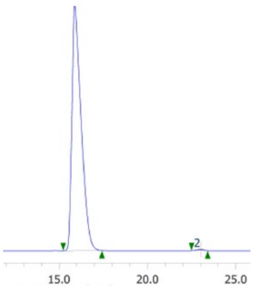

HPLC (CHIRALPAK IF, acetonitrile, flow rate = 0.5 mL/min, l = 254 nm) tR = 15.9 min, 99.6% (major), 23.0 min, 0.4% (minor), 99.2% ee

For **1r**

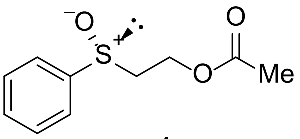

(+)-**1r**

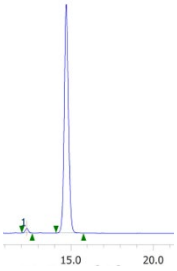

HPLC (CHIRALPAK IF, acetonitrile, flow rate = 0.5 mL/min, l = 254 nm) tR = 12.3 min, 1.6% (minor), 14.7 min, 98.4% (major), 96.8% ee

For **1s**

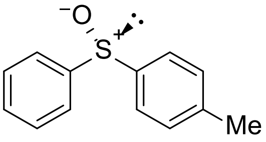

(+)-**1s**

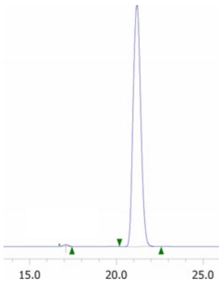

HPLC (CHIRALPAK IG, acetonitrile, flow rate = 0.5 mL/min, l = 254 nm) tR = 17.1 min, 0.5% (minor), 21.2 min, 99.5% (major), 99.0% ee

For **1t**

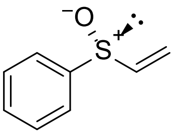

1t

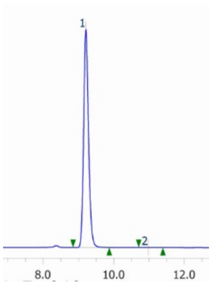

HPLC (CHIRALPAK IG, acetonitrile, flow rate = 0.5 mL/min, l = 254 nm) tR = 9.2 min, 99.9% (major minor), 10.9 min, 0.1% (minor), 99.8% ee

For **1u**

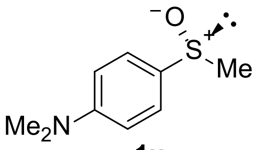

(+)-**1u**

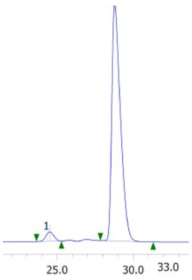

HPLC (CHIRALPAK IA, acetonitrile, flow rate = 0.5 mL/min, l = 254 nm) tR = 24.5 min, 3.4% (minor), 28.8 min, 96.6% (major), 93.2% ee

For **1v**

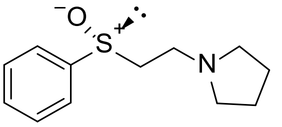

(+)-**1v**

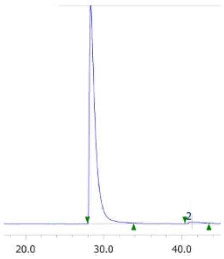

HPLC (CHIRALPAK IG, acetonitrile, flow rate = 0.5 mL/min, l = 254 nm) tR = 28.3 min, 98.7% (major), 41.3 min, 1.3% (minor), 97.4% ee

For **1w**

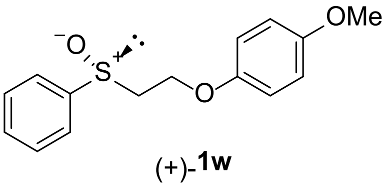

(+)-**1w**

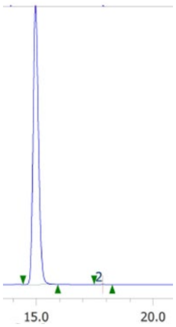

HPLC (CHIRALPAK IA, acetonitrile, flow rate = 0.5 mL/min, l = 254 nm) tR = 15.0 min, 99.9% (major), 17.8 min, 0.1% (minor), 99.8% ee

7. ^{1}H-, ^{13}C-, and 2D-NMR spectra of 1v, 2, 1w.

^{1}H-NMR (400 MHz, $CDCl_3$) of **1v**

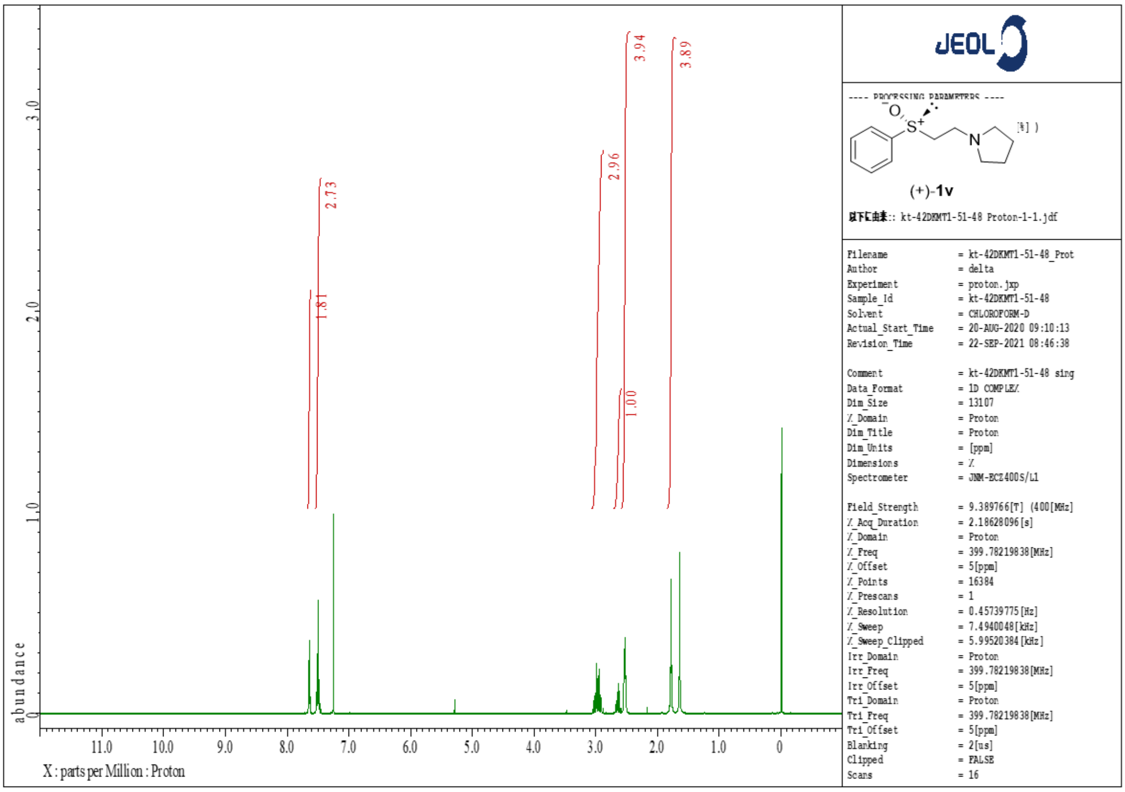

^{13}C {^{1}H}-NMR (100 MHz, $CDCl_3$) of **1v**

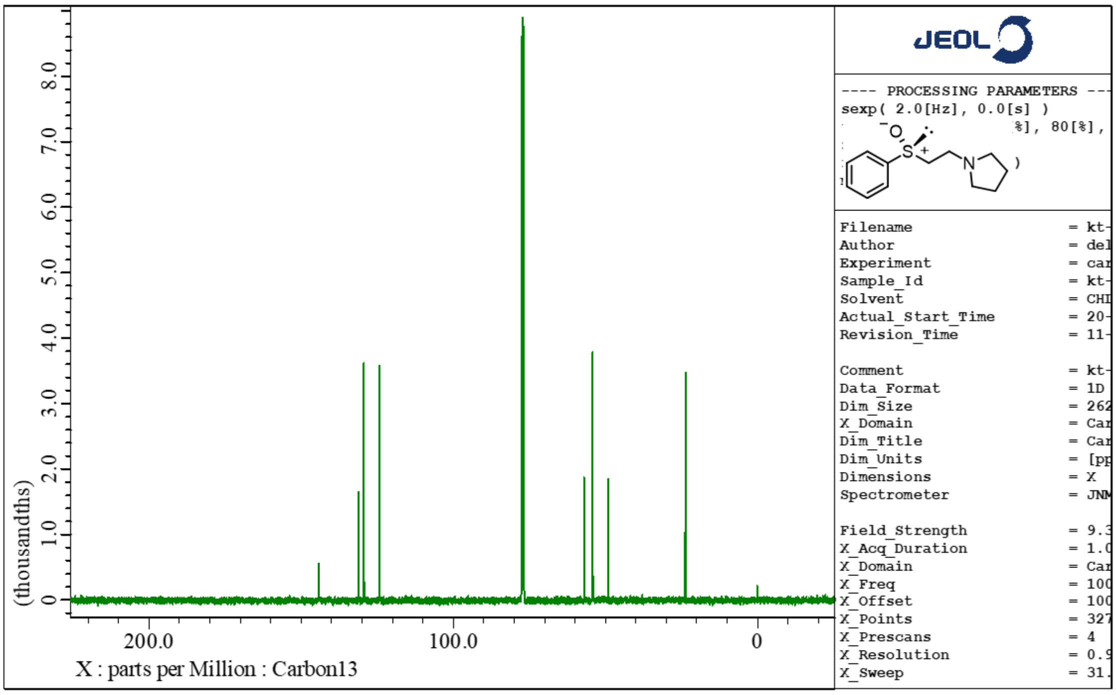

H-H COSY-NMR (400 MHz, $CDCl_3$) of **1v**

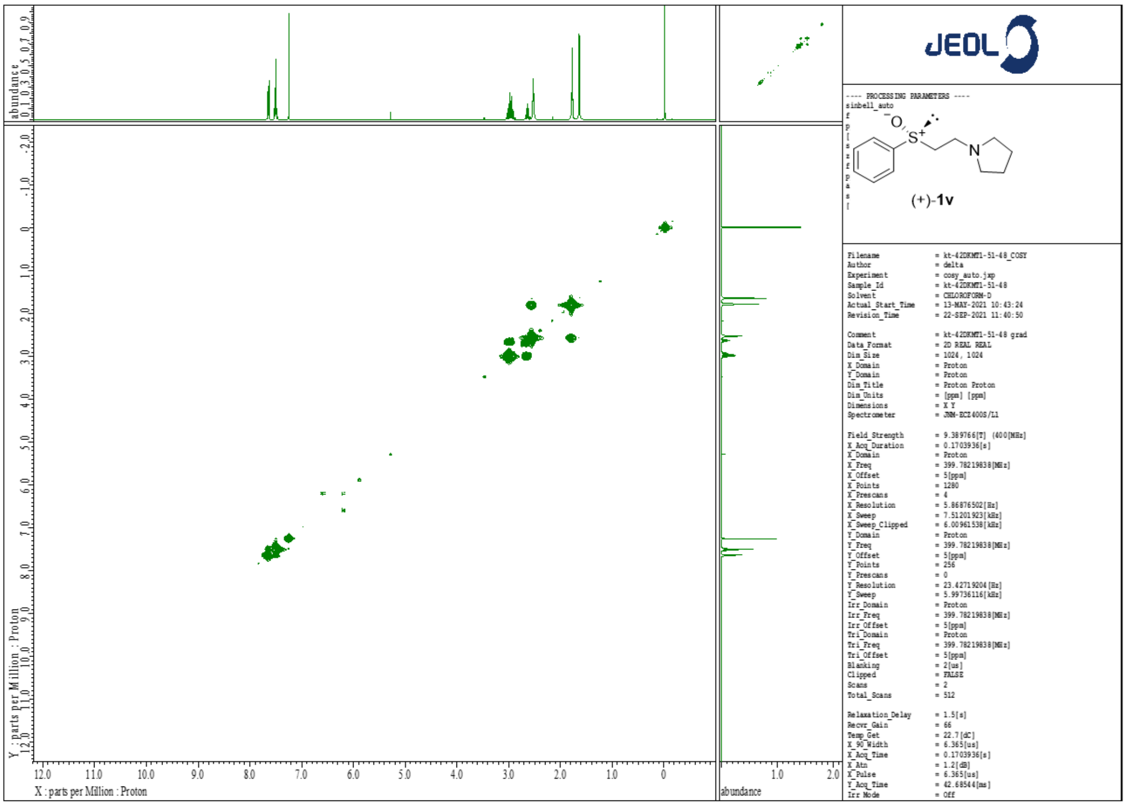

HMQC-NMR ($CDCl_3$) of **1v**

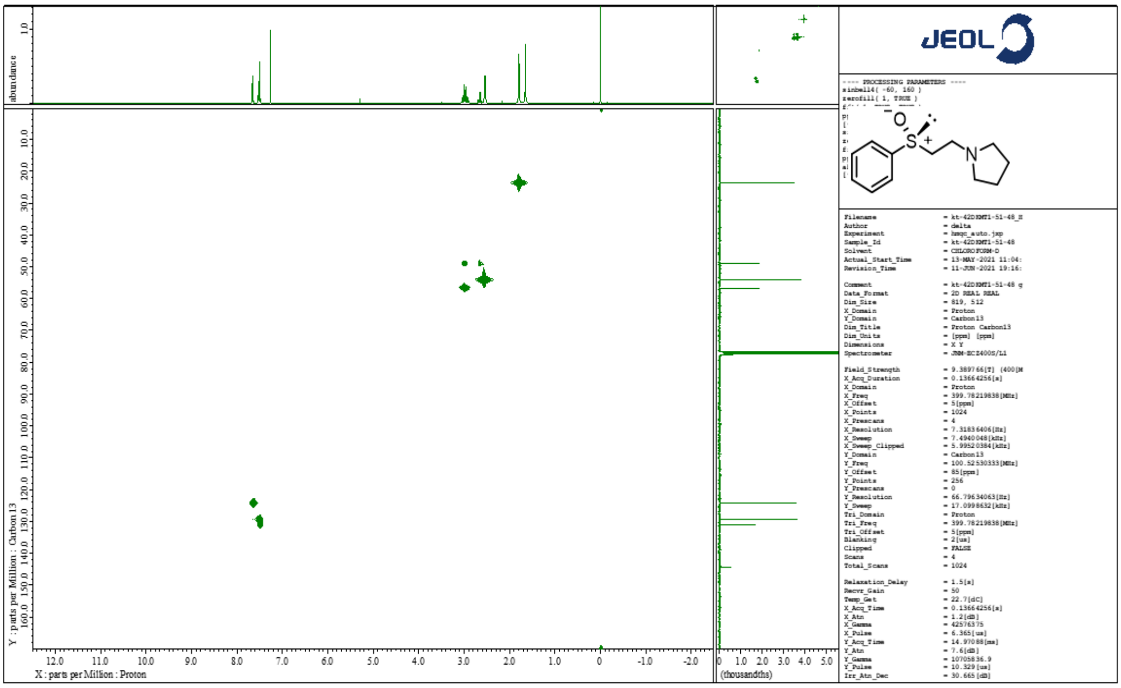

^{1}H-NMR (400 MHz, $CDCl_3$) of **2**

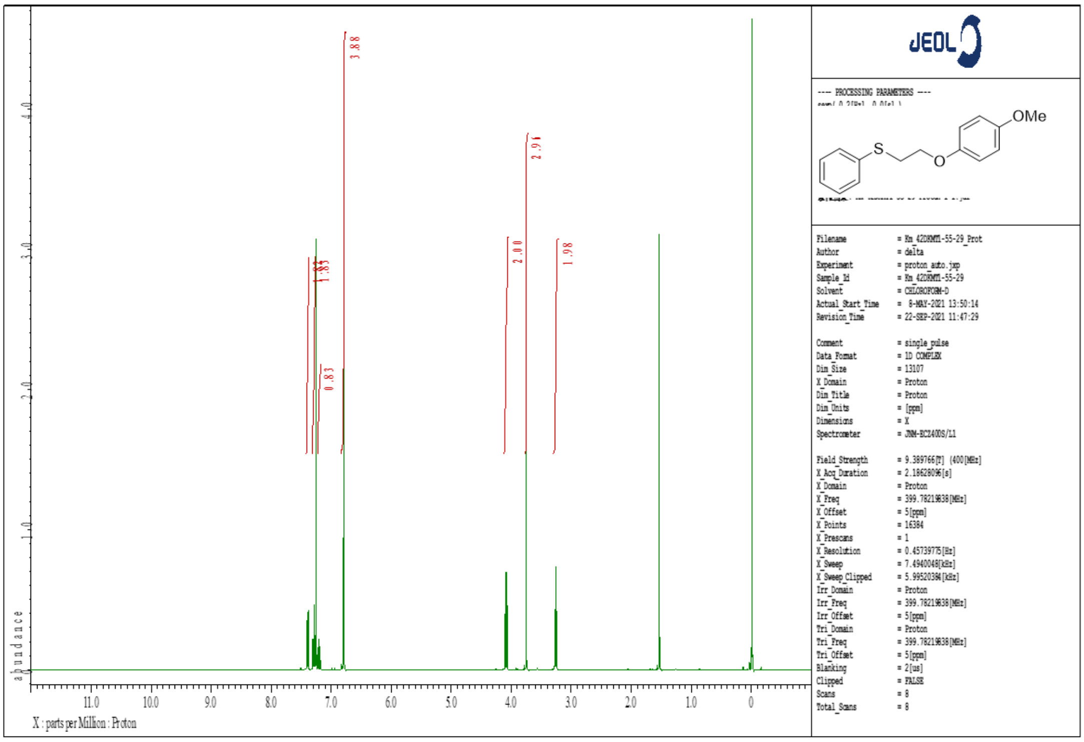

^{13}C {^{1}H}-NMR (100 MHz, $CDCl_3$) of **2**

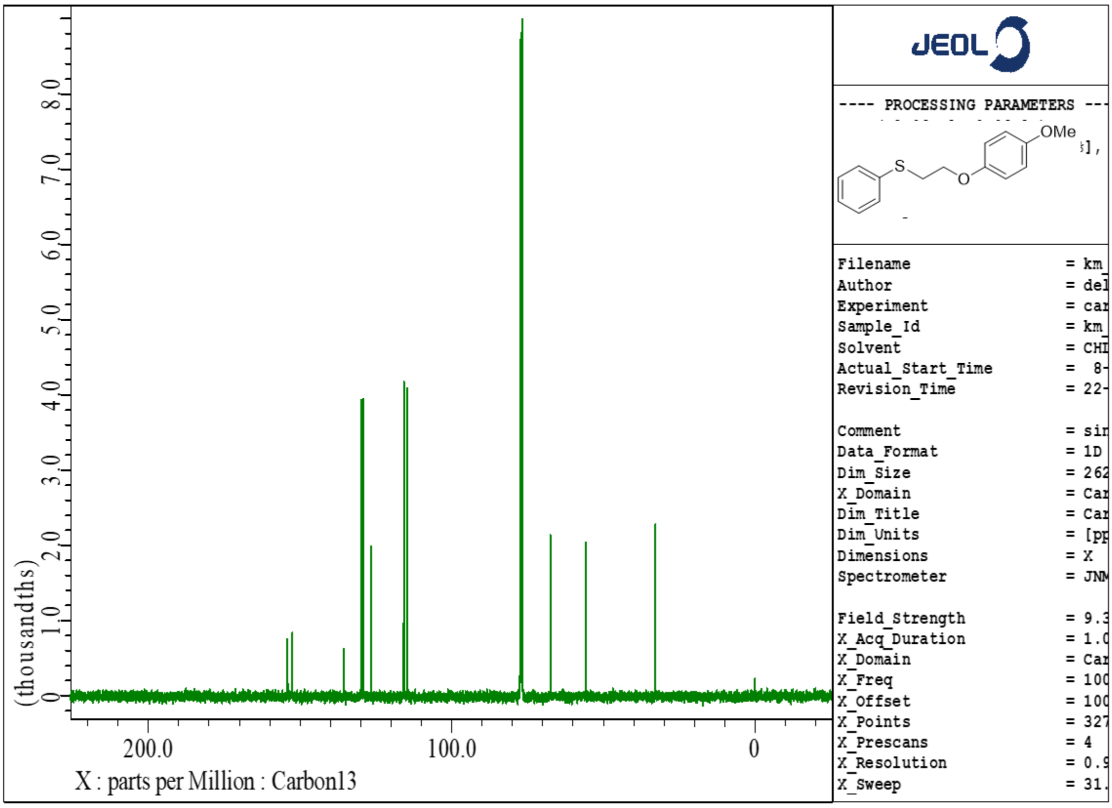

H-H COSY-NMR (400 MHz, $CDCl_3$) of **2**

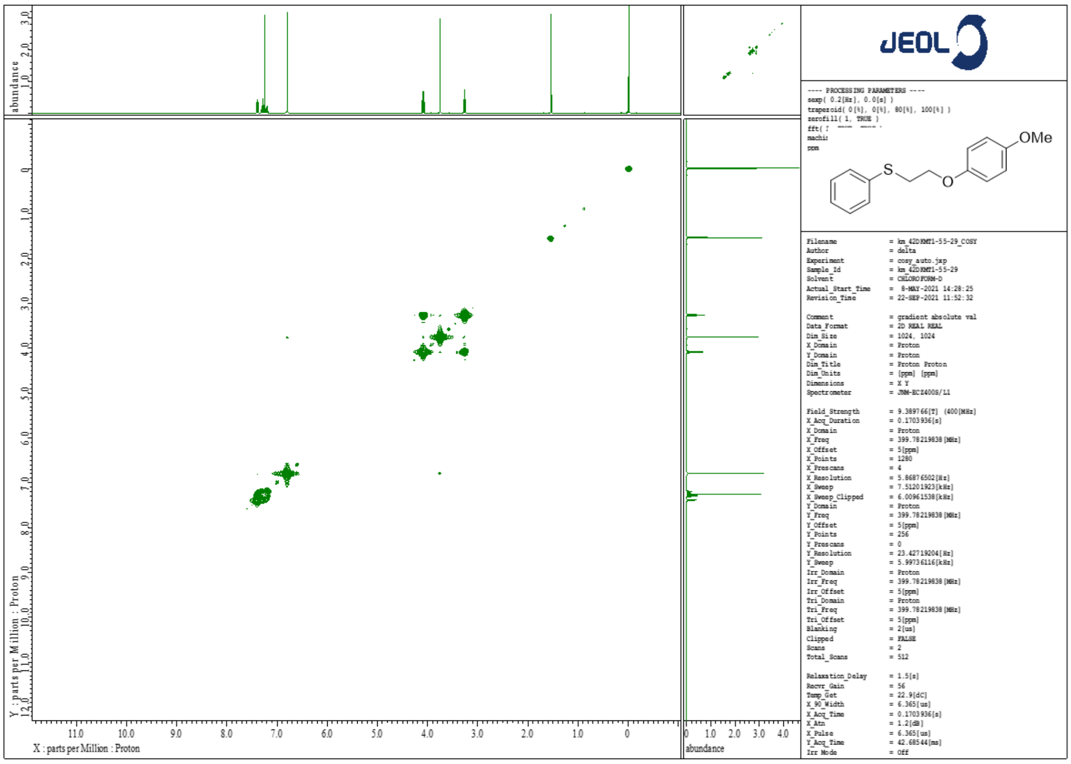

HMQC-NMR ($CDCl_3$) of **2**

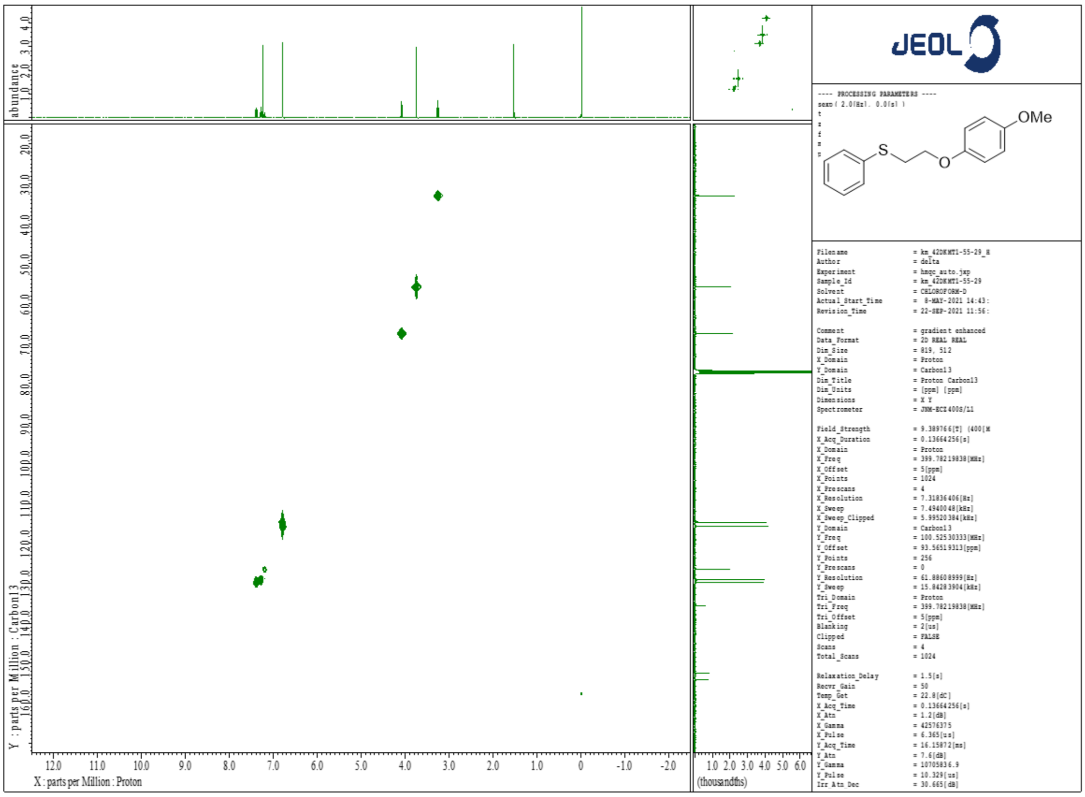

^{1}H-NMR (400 MHz, $CDCl_3$) of **1w**

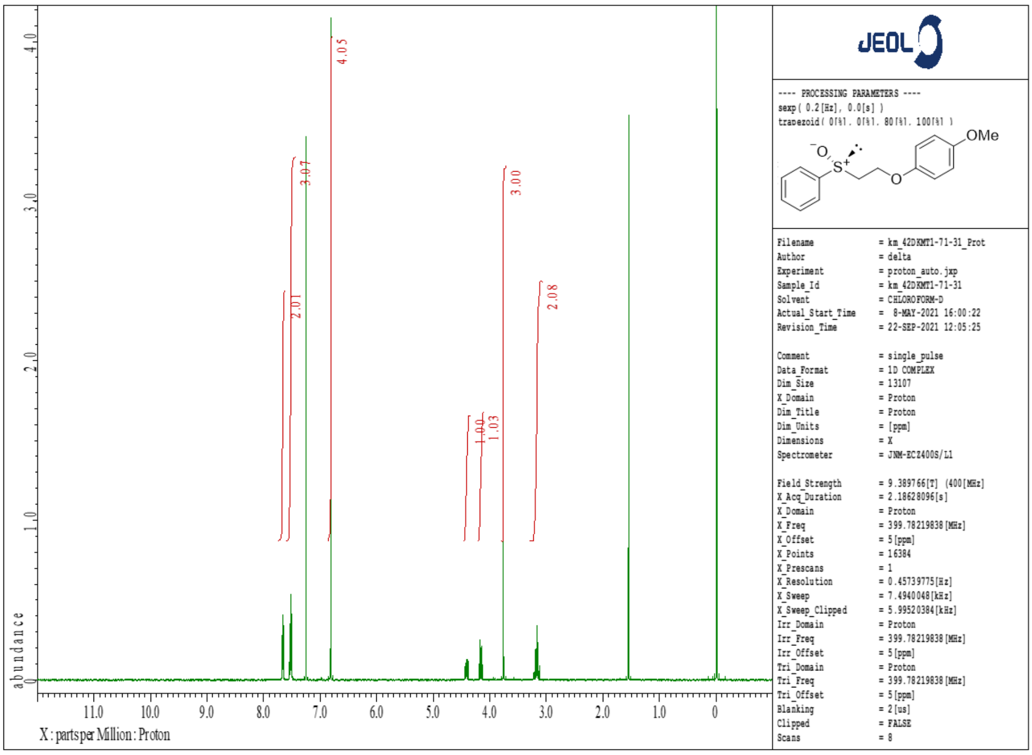

^{13}C {^{1}H}-NMR (100 MHz, $CDCl_3$) of **1w**

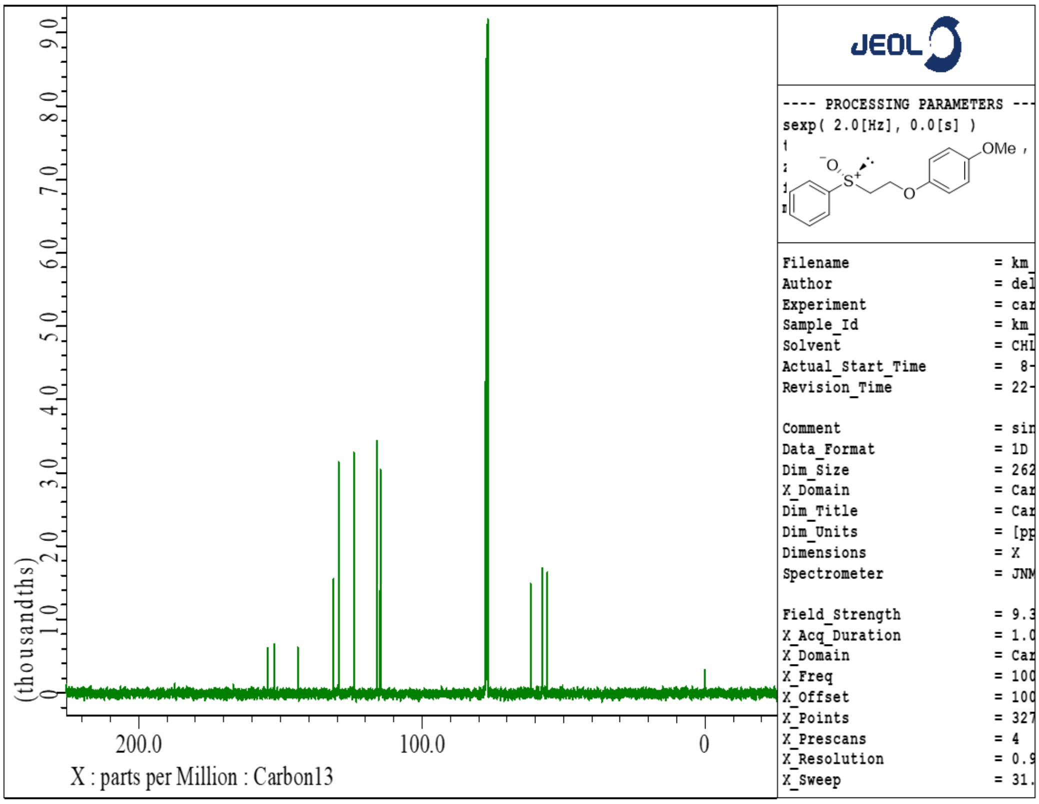

H-H COSY-NMR (400 MHz, $CDCl_3$) of **1w**

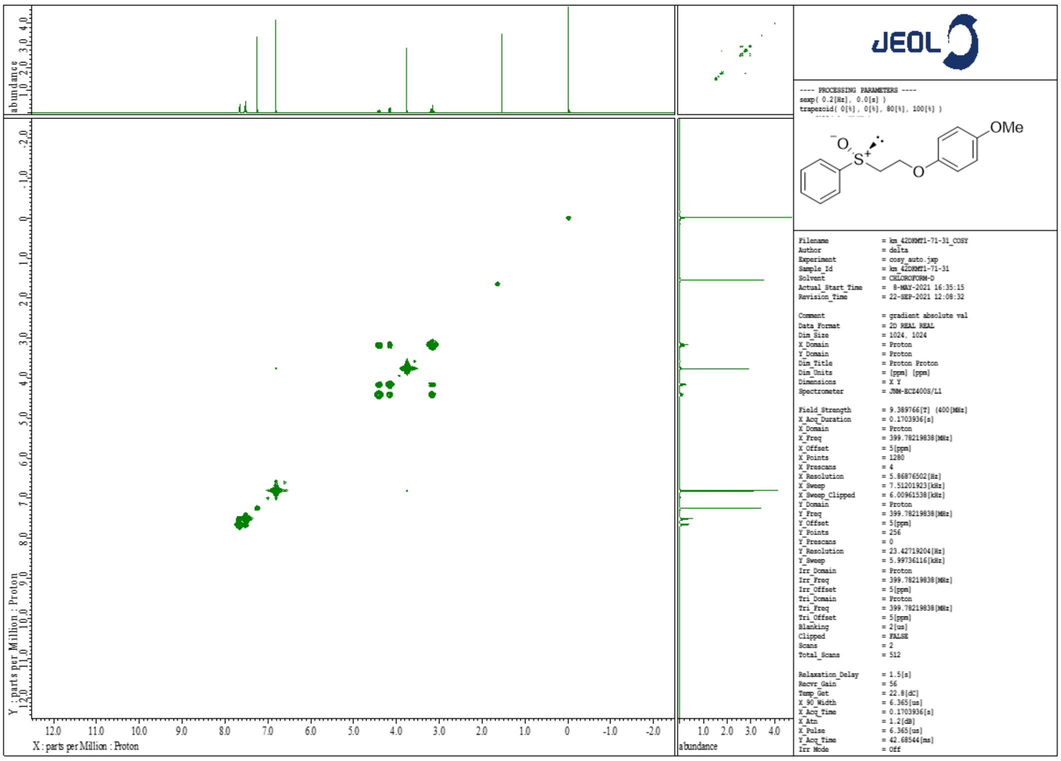

HMQC-NMR ($CDCl_3$) of **1w**

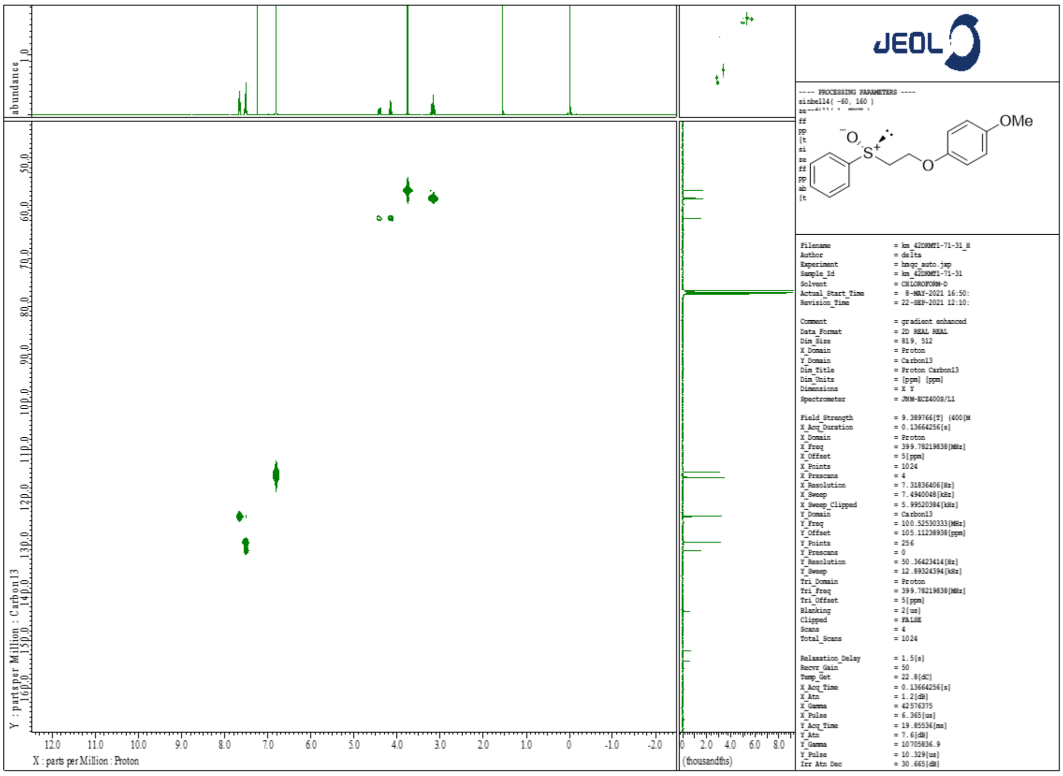

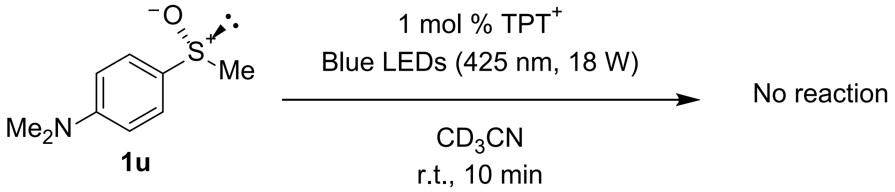
1u
1 mol % TPT+
Blue LEDs (425 nm, 18 W)
CD3CN
r.t., 10 min
No reaction

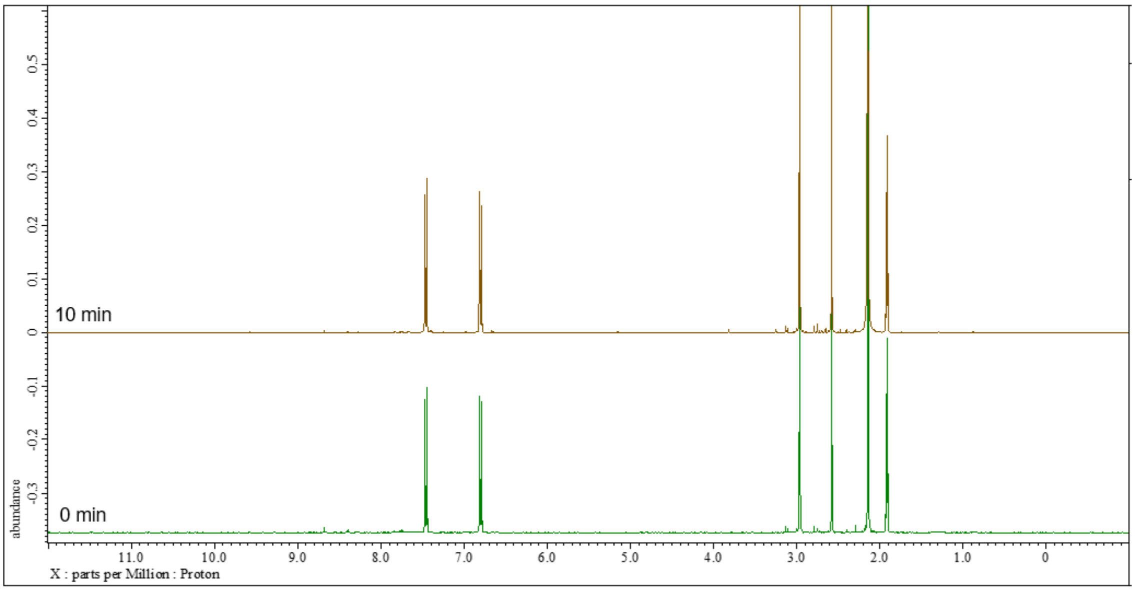
10 min
0 min
abundance
X : parts per Million : Proton

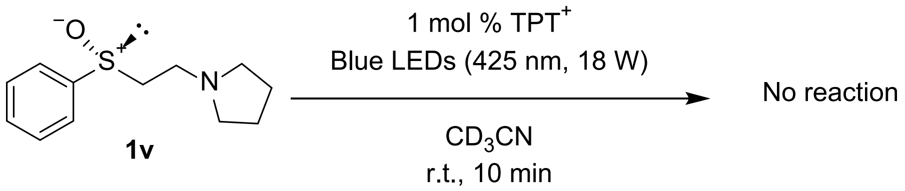

1v
1 mol % TPT+
Blue LEDs (425 nm, 18 W)
CD3CN
r.t., 10 min
No reaction

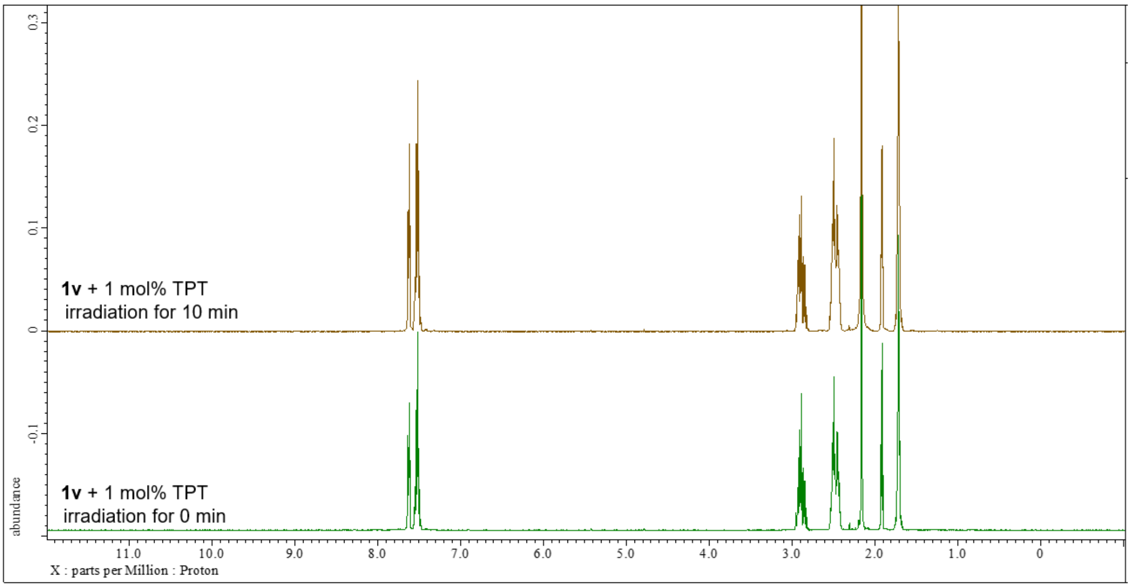

1v + 1 mol% TPT
irradiation for 10 min
1v + 1 mol% TPT
irradiation for 0 min
abundance
X : parts per Million : Proton

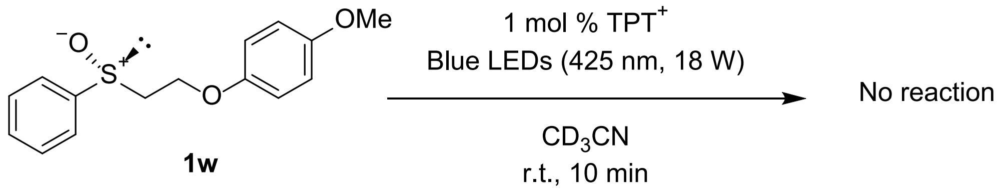
OMe
1w
1 mol % TPT+
Blue LEDs (425 nm, 18 W)
CD3CN
r.t., 10 min
No reaction

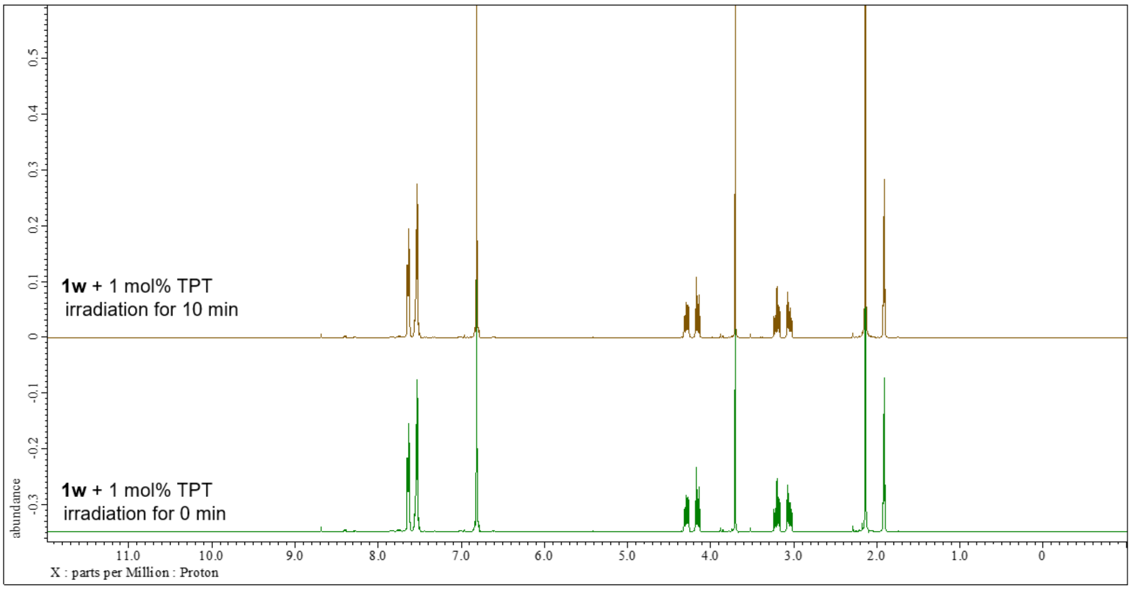
1w + 1 mol% TPT
irradiation for 10 min
1w + 1 mol% TPT
irradiation for 0 min
abundance
X : parts per Million : Proton
